# An Enamide‐Based Domino Reaction for a Highly Stereoselective Synthesis of Tetrahydropyrans

**DOI:** 10.1002/anie.201907565

**Published:** 2019-08-07

**Authors:** Philipp Kramer, Jennifer Grimmer, Michael Bolte, Georg Manolikakes

**Affiliations:** ^1^ Department of Organic Chemistry Technical University Kaiserslautern Erwin-Schrödinger-Strasse Geb. 54 67663 Kaiserslautern Germany; ^2^ Department of Inorganic and Analytical Chemistry Goethe University Frankfurt am Main Max-von-Laue-Strasse 7 60438 Frankfurt am Main Germany

**Keywords:** domino reactions, enamides, Lewis acids, stereoselective synthesis, tetrahydropyrans

## Abstract

A novel method for the highly stereoselective synthesis of tetrahydropyrans is reported. This domino reaction is based on a twofold addition of enamides to aldehydes followed by a subsequent cyclization and furnishes fully substituted tetrahydropyrans in high yields. Three new σ‐bonds and five continuous stereogenic centers are formed in this one‐pot process with a remarkable degree of diastereoselectivity. In most cases, the formation of only one out of 16 possible diastereomers is observed. Two different stereoisomers can be accessed in a controlled fashion starting either from an *E*‐ or a *Z*‐configured enamide.

The tetrahydropyran ring is an abundant structural motif in natural products and medicinally relevant molecules.^1^ Hence, various approaches for the synthesis of this scaffold have been developed, with a particular focus on the stereoselective construction of highly substituted tetrahydropyrans.^2^ Although these strategies enable a rapid assembly of the tetrahydropyran core, the preparation of pentasubstituted tetrahydropyrans with precise control over all five stereocenters remains a significant synthetic challenge.^3^ Herein, we report a novel approach based on a twofold addition of enamides to aldehydes. This conceptually new strategy provides a versatile platform for the highly diastereoselective synthesis of fully substituted tetrahydropyrans with five continuous stereocenters.

Recently, we have reported the stereoselective synthesis of 1,3‐diamines^4^ and dihydropyrimido[2,1‐a]isoindole‐6(2*H*)‐ones.^5^ Both transformations are based on the initial addition of an enamide or enimide to an in situ generated *N*‐acylimine. In general, the nucleophilic addition of enamides and enecarbamates to reactive electrophiles offers an attractive opportunity for the rapid construction of molecular complexity.^6^ Although reactions with highly electrophilic glyoxylic acid derivatives^7^ or activated ketones^8^ have been described (Scheme 1 a), the addition of enamides to simple, non‐activated aldehydes has, to the best of our knowledge, not been reported so far. We envisioned that such a direct addition could open an attractive synthetic route to 1,3‐aminoalcohols (Scheme 1 b). However, an initial reaction between enamide **2 a** and benzaldehyde (**1 a**) in the presence 1.1 equivalents of BF_3_⋅OEt_2_ as Lewis acid in dichloromethane did not afford the expected 1,3‐aminoacohol. Instead tetrahydropyran **3 a** was isolated in 44 % yield. During this unexpected reaction, three new bonds (2×C−C and 1× C−O bond) and five new stereocenters are formed in a simple one‐pot process with a remarkable degree of stereoselectivity.^9^ Out of 16 possible diastereomers, only the tetrahydropyran **3 a** could be detected in the crude reaction mixture. Since the rapid construction of any organic molecule containing three or more continuous stereocenters is still a tremendous synthetic challenge,^10^, ^11^ we decided to further investigate this novel transformation.

**Scheme 1:**
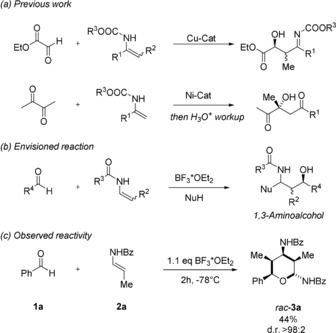
Previous work, envisioned reaction, and observed reactivity. Bz=benzoyl.

Since two molecules of the enamide are incorporated into the final product, we started our optimization studies by increasing the amount of **2 a** (Table 1, entry 1). With 2.5 equivalents of the enamide (**2 a**), tetrahydropyran **3 a** was obtained in almost quantitative yield with a slightly decreased diastereoselectivity (d.r.=92:8). A decreased amount of BF_3_⋅OEt_2_ of 50 and 25 mol % led to the desired product without changes in the yield and an improved stereoselectivity (entries 2 and 3). Indeed, only diastereomer **3 a** could be observed in the crude reaction mixture by ^1^H NMR spectroscopy. Decreasing the amount of BF_3_⋅OEt_2_ to 5 mol % led to a significant decrease in the yield and a lower degree of stereoselctivity (entry 4).

**Table 1:** Optimization of the reaction conditions.^[a]^ 
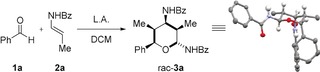

Entry	Lewis acid	Cat. [mol %]	Yield [%]^[b]^	d.r.^[c]^
1	BF_3_⋅OEt_2_	110	97	92:8
2	BF_3_⋅OEt_2_	50	97	>98:2
3	BF_3_⋅OEt_2_	25	97	>98:2
4	BF_3_⋅OEt_2_	5	57	87:13
5	TiCl_4_, SnCl_4_, TMSOTf, HBF_4_	25–100	–	–

[a] Reaction conditions: DCM, −78 °C to rt, 16 h. [b] Overall yield of isolated product after column chromatography. [c] The diastereomeric ratio (d.r.) was determined by ^1^H NMR spectroscopic analysis of the crude reaction mixture. Bz=Benzoyl, L.A.=Lewis acid. Structure of **3 a** in the solid state (methyl and aromatic H atoms are omitted for clarity).

Interestingly, all other tested Lewis or Brønsted acids, such as TiCl_4_, SnCl_4_, TMSOTf, or HBF_4_, did not afford the tetrahydropyran product **3 a** at all (entry 5). Complex mixtures and decomposition of the enamide **2 a** were observed in these cases. It seems that only BF_3_⋅OEt_2_ displays the required balanced reactivity necessary for mediating the transformation without concomitant decomposition of the starting materials and/or the product.

With the optimized conditions in hand, we investigated the scope of this transformation. Initially, reactions of enamide **2 a** with different aldehydes **1** were studied (Scheme 2).

**Scheme 2:**
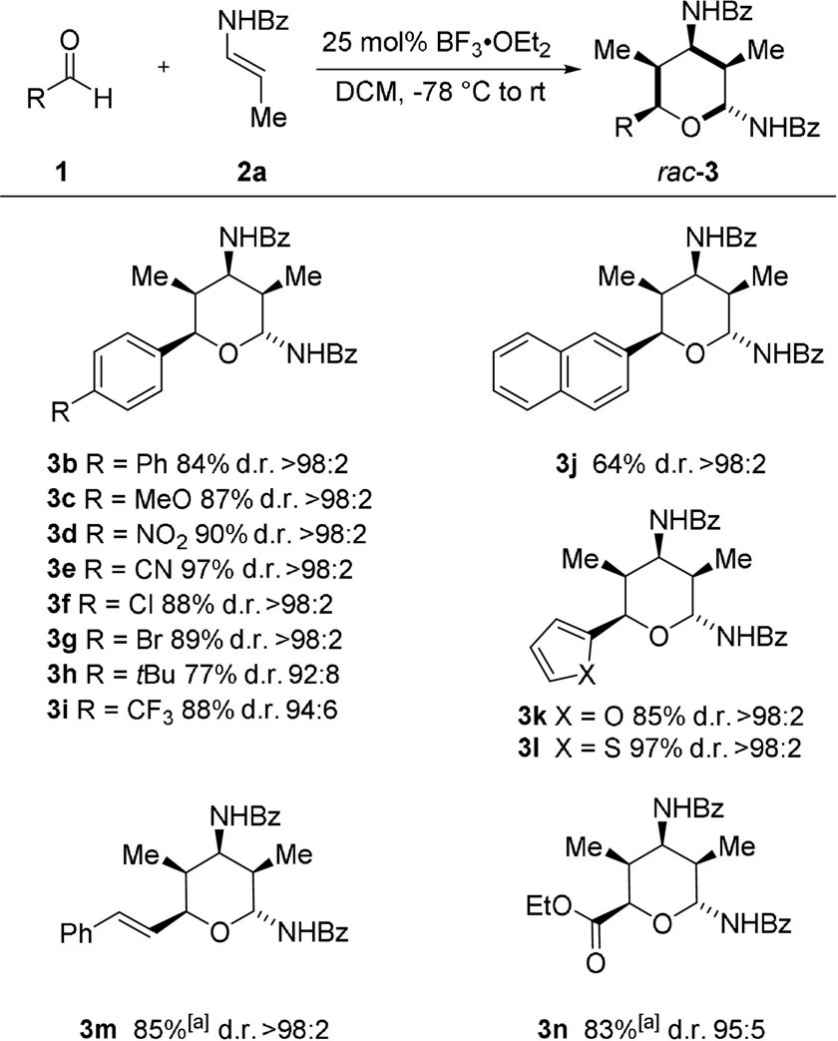
Substrate scope with aryl aldehydes. The yields are of isolated diastereochemically pure compound (d.r.>98:2) after column chromatography. The diastereomeric ratio (d.r.) was determined by ^1^H NMR spectroscopic analysis of the crude reaction mixture. [a] Prepared with 1.1 equiv BF_3_⋅OEt_2_. Bz=benzoyl.

A broad range of aromatic aldehydes bearing electron‐donating or ‐withdrawing groups furnished the desired tetrahydropyrans **3 b**–**j** in a yield of 64–97 % and with excellent diastereoselectivities. In most cases, only one diastereomer could be detected in the crude reaction mixture. For some aldehydes, a slightly lower degree of stereoselectivity was observed (**3 h** and **3 i**). Reactions with heteroaryl aldehydes, such as furfural or thiophene‐2‐carbaldehyde, proceeded with similar efficiency, thereby leading to the tetrahydropyrans **3 k** and **3 l** in yields of 85 % and 97 %, respectively, and excellent diasteromeric ratios. Alkyl aldehydes proved to be not suitable for this domino process, affording only various unidentified decomposition products. Reactions of enamide **2 a** with cinnamyl aldehyde **1 m** and ethyl glyoxalate **1 n** furnished the tetrahydropyrans **3 m** and **3 n**, both bearing an additional functional group handle for further transformations, in high yields and excellent diastereoselectivities (Scheme 2). Stoichiometric amounts of BF_3_⋅OEt_2_ proved to be necessary for an efficient conversion of these two aldehydes.

Next, we investigated the reaction of different *E*‐configured enamides **2** with benzaldehyde **1 a**. As shown in Scheme 3, a variety of different benzamide‐derived enamides proved to be suitable substrates for this domino transformation. The desired products **3 o**–**3 s** were obtained in uniformly high yields and diastereoselectivities. Unfortunately, this process proved to be somewhat sensitive towards modifications of the enamide structure. Reactions with enamides **2 i**–**k**, bearing either a different substitution pattern or no additional substituents at the double bond, or of the succinyl‐derived enamide **2 l**, did not afford the desired products. Only in the case of the ethyl‐substituted enamide could the desired tetrahydropyran **3 u** be isolated in 77 % yield and excellent diastereoselectivity.

**Scheme 3:**
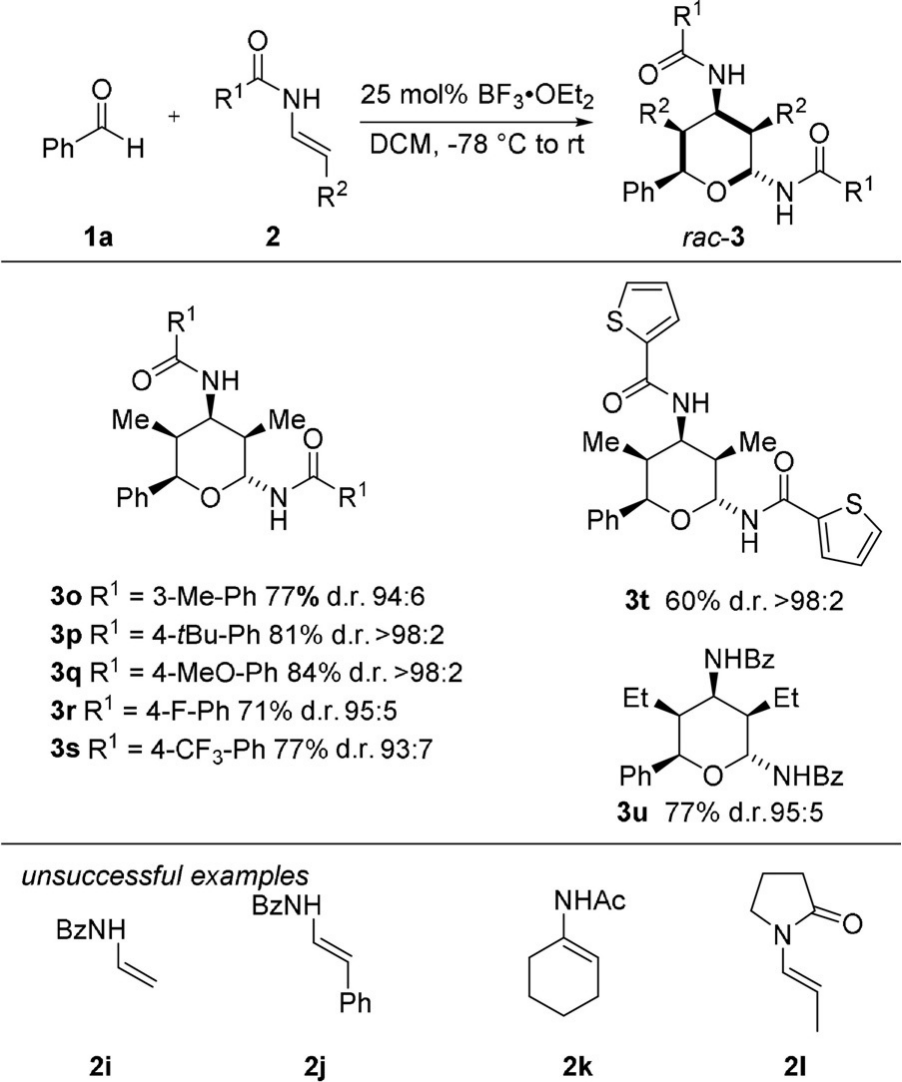
Substrate scope with different (*E*)‐enamides. The yields are of isolated diastereochemically pure compound (d.r.>98:2) after column chromatography. The diastereomeric ratio (d.r.) was determined by ^1^H NMR spectroscopic analysis of the crude reaction mixture. Bz=Benzoyl.

To enable a more facile subsequent modification of the obtained tetrahydropyran scaffold, the reactions of the two enecarbamates **4 a** and **4 b** with benzaldehyde **1 a** were investigated (Scheme 4). Stoichiometric amounts of BF_3_⋅OEt_2_ were necessary for an efficient conversion, furnishing the Cbz‐ and Boc‐protected 2,4‐diaminotetrahydropyrans **5 a** and **5 b** in 89 % and 30 % yield, respectively, as single diastereomers (Scheme 4).

**Scheme 4:**
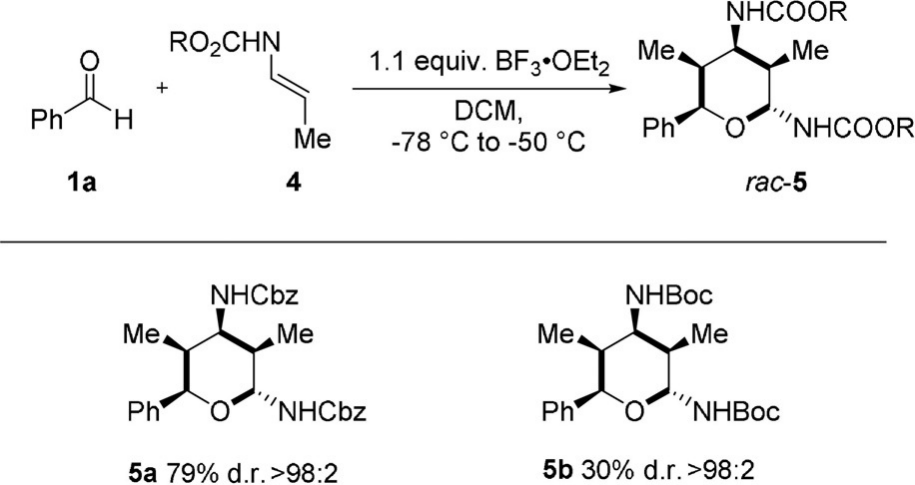
Reaction of enecarbamates. Overall yield of isolated product after column chromatography; The diastereomeric ratio (d.r.) was determined by ^1^H NMR spectroscopic analysis of the crude reaction mixture.

Next, we studied the reactivity of (*Z*)‐configured enamide **2 a** towards different (hetero)arylaldehydes **1** (Scheme 5). In general, these transformations proved to be more sluggish and required stoichiometric amounts of BF_3_⋅OEt_2_. The reaction of (*Z*)‐**2 a** with benzaldehyde did proceed in good overall yield and moderate stereoselectivity, affording tetrahydropyran **6 a** as the major diastereomer together with **3 a** as the only other detectable diastereomer. In the case of *p*‐anisaldehyde and 2‐thiophenecarbaldehyde, tetrahydropyrans **6 c** and **6 l** were obtained in similar yields and stereoselectivities. Taking into account that up to 16 diastereomers could be formed in this process, the selective formation of only two diastereomers is still quite remarkable.

**Scheme 5:**
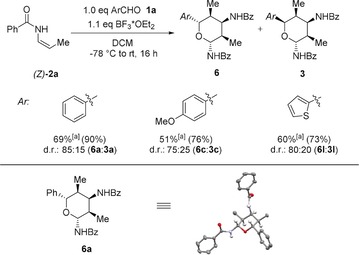
Reactions with (*Z*)‐enamide **2 a**. [a] Yield of isolated diastereochemically pure compound **6** (d.r.>98:2) after column chromatography. Values in parentheses represent the overall yield of all isolated diastereomers. The diastereomeric ratio (d.r.) was determined by ^1^H NMR spectroscopic analysis of the crude reaction mixture. Bz=Benzoyl. Structure of **6 a** in the solid state (methyl and aromatic H atoms are omitted for clarity).

Furthermore, we could assign the relative configuration of **6 a** by single‐crystal X‐ray diffraction, which revealed a 1,5‐*syn* relationship. Interestingly, the use of (*Z*)‐enamide **2 a** only impacts the configuration of C5, which presumably arises from the initial addition of the enamide to the aldehyde. The configuration of all other stereocenters is not affected. Currently, we assume the following reaction mechanism (Scheme 6). After activation of the aldehyde with BF_3_⋅OEt_2_, a carbonyl‐ene type reaction with the first enamide molecule occurs, leading to *N*‐acyliminium ion **II**. Addition of a second enamide molecule in an aza‐ene‐type reaction affords a second *N*‐acyliminium ion intermediate **III**. Intramolecular addition of the alcohol moiety terminates the domino process and furnishes the tetrahydropyran product **3 a**. So far, this simplified model cannot explain the observed stereochemical course of the reaction and, in particular, the role of the enamide configuration. However, it seems, that the initially formed stereocenter at C4 exerts a dominant influence on all subsequently formed stereocenters.

**Scheme 6:**
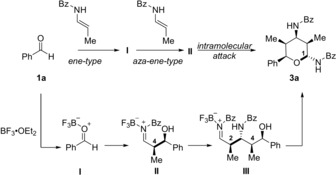
Preliminary reaction mechansim. Bz=Benzoyl.

In summary, we have developed a novel method for the highly stereoselective synthesis of pentasubstituted tetrahydropyrans. This BF_3_‐catalyzed domino transformation offers a versatile and highly modular approach for the generation of structural complexity from simple building blocks. Based on the twofold addition of an enamide to an aldehyde and a subsequent cyclization, three new σ‐bonds and five continuous stereocenters are formed in a simple one‐pot operation. The whole process proceeds with an outstanding degree of stereocontrol and delivers in most cases only one out of 16 possible diastereomers. By starting from either the (*E*)‐ or the (*Z*)‐configured enamides, two different diastereomers of a tetrahydropyran scaffold can be prepared in a controlled manner. Further investigations on the reaction mechanism, the use of a chiral catalyst, as well as applications in the synthesis of other heterocyclic structures are currently ongoing in our laboratory.

## Conflict of interest

The authors declare no conflict of interest.

## Supporting information

As a service to our authors and readers, this journal provides supporting information supplied by the authors. Such materials are peer reviewed and may be re‐organized for online delivery, but are not copy‐edited or typeset. Technical support issues arising from supporting information (other than missing files) should be addressed to the authors.

Supplementary
