# Supplementary material for: An Enamide‐Based Domino Reaction for a Highly Stereoselective Synthesis of Tetrahydropyrans
Source: Angew Chem Int Ed Engl. 2019 Aug 7;58(37):13056–9. doi: 10.1002/anie.201907565 (PMC6772187; doi:10.1002/anie.201907565)
Supplement: Supplementary file 1 — Supplementary [file ANIE-58-13056-s001.pdf]

Angewandte Chemie
Eine Zeitschrift der Gesellschaft Deutscher Chemiker

Supporting Information

An Enamide-Based Domino Reaction for a Highly Stereoselective Synthesis of Tetrahydropyrans

*Philipp Kramer, Jennifer Grimmer, Michael Bolte, and Georg Manolikakes**

anie_201907565_sm_miscellaneous_information.pdf

Supporting Information

1 General Information 2

2 Preparation and Analytical Data 4

2.1 Enamides 4

2.2 Synthesis of tetrahydro-2H-pyranes 4

3 NMR Data 31

4 X-ray Data 59

5 References 65

1 General Information

Experimental methods

Reactions. Unless otherwise mentioned, all reactions were carried out under an argon or nitrogen atmosphere in flame dried glassware applying standard Schlenk techniques. All yields refer to isolated yields of compounds estimated to be > 95% pure as determined by ^{1}H-NMR.

Chromatography Column chromatography was performed with Silica 60 (0.04-0.063 mm, 230-400 mesh) and the specified solvent mixture. Thin layer chromatography was performed on aluminum sheets coated with SiO_2 (TLC silica gel 60 F_{254}). The spots were visualized by ultraviolet light, iodine, cerium ammonium molybdate (CAM) or vanillin.

Solvents Solvents for reactions and column chromatography were obtained from different commercial suppliers in >97% purity and used as received. Solvents for column chromatography were technical standard.

Materials. All starting materials obtained from commercial sources were used without further purification. Anhydrous $BF_3{\cdot}OEt_2$ was obtained from different providers and used directly. Prolonged storage times were avoided.

Analytical Data and Instrumentation

NMR spectroscopy Proton nuclear magnetic resonance spectra (^{1}H NMR) and carbon spectra (^{13}C NMR) were recorded at 300, 400 or 500 MHz (^{1}H), 75, 101 or 126 MHz (^{13}C) and 376 MHz (^{19}F), respectively. Chemical shifts are reported as δ - values relative to the residual $CDCl_3$ (δ = 7.26 ppm for ^{1}H and δ = 77.16 ppm for ^{13}C), DMSO-d_6 (δ = 2.50 ppm for ^{1}H and δ = 39.51 ppm for ^{13}C). Coupling constants (*J*) are given in Hz and multiplicities of the signals are abbreviated as follows: s = singlet; d = doublet; t = triplet; q = quartet; sp = septet; m = multiplet; dd = doublet of doublets and dt = doublet of triplets dqd = doublet of quartets of doublets.

Melting points. Melting points are reported uncorrected.

Mass spectrometry. Mass spectra (MS) were measured using electrospray ionization (ESI) techniques. High resolution mass spectra (HRMS) were measured using matrix-assisted laser desorption/ionization (MALDI) techniques or electron ionization mass spectroscopy (EI-MS-TOF).

Infrared spectroscopy. Infrared spectra (IR) of neat substances were recorded on a FT-IR (Fourier transform infrared spectroscopy) spectrometer equipped with a diamond universal ATR sampling technique (attenuated total reflectance). The absorption bands are reported in wave numbers (cm^{-1}).

Diastereomeric ratio. The diastereomeric ratios (d.r.) were determined via ^{1}H-NMR analysis of the unpurified product after aqueous workup and after isolation via column chromatography.

A diastereomeric ratio of d.r.> 98:2 indicates that no other isomer was observed by ^{1}H NMR. Yields refer to isolated yields of the analytically pure tetrahydropyran (d.r.> 98:2). If other diastereomers could be isolated, a combined yield is given in addition. Minor diastereomers were not fully characterized. In some cases no minor isomers could be isolated after column chromatography, although their formation was observed via NMR analysis of the crude reaction mixture. Presumably, small amounts of the side products were lost during column chromatography.

The relative configuration of the pyranes **3a**, **e**, **g**, **m** and **6a** had been determined by X-Ray crystallography. All other tetrahydropyrans were assigned in analogy via ^{3}J coupling constants and NOE experiments.

Crystal structure determinations. Data for all structures were collected on a STOE IPDS II two-circle diffractometer with a Genix Microfocus tube with mirror optics using MoK_{α} radiation (λ = 0.71073 Å). The data were scaled using the frame scaling procedure in the *X-AREA* program system (Stoe & Cie, 2002). The structures were solved by direct methods using the program *SHELXS* (Sheldrick, 2008) and refined against F^2 with full-matrix least-squares techniques using the program *SHELXL* (Sheldrick, 2008).

.

2 Preparation and analytical data

2.1 Enamides

Enamides **2a-2h** and Enecarbamates **4a-b** were synthesized from the corresponding *N*-Allylamides or *N*-Allylcarbamates via the isomerization-protocol of Halli *et al.*[1]

2.2 Synthesis of tetrahydro-2H-pyranes

Typical procedure 1

A flame dried and argon flushed Schlenk tube, equipped with a septum and a magnetic stirrer, was charged with aldehyde **1** (0.25 mmol, 1.0 equiv.), 1.25 mL DCM and cooled to -78 °C. $BF_3 \cdot OEt_2$ was added dropwise and the reaction was stirred for 30 min. Then the enamide derivative (0.63 mmol, 2.5 equiv.) in 1.25 mL dichloromethane was added dropwise. The reaction was allowed to warm to -50 ° or rt until TLC showed complete consumption of the aldehyde. Saturated aqueous $NaHCO_3$ (5 mL) was added and The organic layer was separated and the aqueous phase was extracted with dichloromethane (3x 10 mL). The combined organic layers were dried over Na_2SO_4, filtered and the solvents were evaporated under reduced pressure. Purification of the crude residue by flash column chromatography afforded the analytically pure product.

2.2.1 Tetrahydro-2H-pyrane **3a**

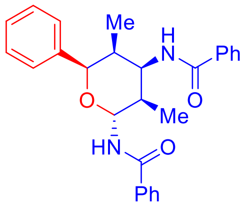

3a

Prepared according to TP1 from benzaldehyde **1a** (25 µL, 0.25 mmol, 1.0 equiv.), $BF_3{\cdot}OEt_2$ (1 M in DCM, 0.13 mL, 63 µmol, 0.25 equiv.), (*E*)-enamide **2a** (101 mg, 0.63 mmol, 2.5 equiv.) in a total of 2.5 mL dichloromethane. The reaction was allowed to warm to rt overnight. Column chromatography (*n*-hexane:EtOAc = 9:1→4:1→7:3→1:1) afforded the desired tetrahydropyran **3a** as a colorless foam (102 mg, 97%, isolated d.r. >98: 2; d.r. of the crude mixture >98: 2 as determined by ^{1}H NMR analysis of the unpurified product after aqueous workup).

R_f (*n*-hexane:EtOAc = 6:4) 0.2.

m.p. 195-210 °C.

^{1}H NMR (*400 MHz, $CDCl_3$*) δ 7.82 (d, *J* = 7.4 Hz, 2H), 7.61 (d, *J* = 7.3 Hz, 2H), 7.55 – 7.49 (m, 2H), 7.43 (t, *J* = 7.8 Hz, 6H), 7.36 (t, *J* = 7.6 Hz, 2H), 7.30 – 7.26 (m, *J* = 7.5 Hz, 1H), 7.07 (d, *J* = 8.3 Hz, 1H), 6.36 (d, *J* = 9.4 Hz, 1H), 5.88 (dd, *J* = 8.1, 6.1 Hz, 1H), 5.20 (d, *J* = 4.9 Hz, 1H), 4.78 (dt, *J* = 9.4, 4.6 Hz, 1H), 2.75 – 2.48 (m, 1H), 2.30 – 2.15 (m, 1H), 1.23 (d, *J* = 7.0 Hz, 3H), 0.82 (d, *J* = 7.3 Hz, 3H).

^{13}C NMR (*126 MHz, $CDCl_3$*) δ 167.9, 167.8, 139.8, 134.3, 134.0, 132.2, 131.9, 128.9, 128.8, 128.6, 127.4, 127.2, 126.9, 126.0, 79.4, 73.6, 50.4, 38.4, 37.7, 14.9, 12.5.

IR (ATR, ν in cm^{-1}): 3332 (w), 2964 (w), 1665 (m),1630 (s), 1580 (m), 1520 (s), 1487 (s), 1467 (w), 1377 (w), 1318 (m), 1271 (m), 1183 (w), 1074(w), 1017 (s), 847 (w), 746 (m), 684 (s).

HRMS (MALDI) m/z calcd for $C_{27}H_{29}N_2O_3$ 429.2173 $[M+H]^+$, found 429.2169 $[M+H]^+$.

2.2.2 Tetrahydro-2H-pyran **3b**

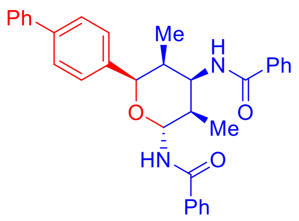

3b

Prepared according to TP1 from 1,1'-biphenyl]-4-carbaldehyde **1b** (46 mg, 0.25 mmol, 1.0 equiv.), $BF_3{\cdot}OEt_2$ (1 M in DCM, 0.13 mL, 63 µmol, 0.25 equiv.), (*E*)-enamide **2a** (101 mg, 0.63 mmol, 2.5 equiv.) in a total of 2.5 mL dichloromethane. The reaction was allowed to warm to rt overnight. Column chromatography (*n*-hexane:EtOAc = 9:1→4:1→7:3→1:1) afforded the desired tetrahydropyran **3b** as a colorless foam (106 mg, 84%, isolated d.r. >98:2; d.r. of the crude mixture >98:2 as determined by ^{1}H NMR analysis of the unpurified product after aqueous workup).

R$_f$ (*n*-hexane:EtOAc = 6:4) 0.34.

m.p. 63-74 °C.

^{1}H NMR (400 MHz, *CDCl$_3$*) δ 7.84 (d, J = 7.4 Hz, 1H), 7.60 (t, J = 7.3 Hz, 2H), 7.56 – 7.34 (m, 5H), 6.38 (d, J = 9.5 Hz, 1H), 5.95 – 5.89 (m, 1H), 5.26 (d, J = 4.8 Hz, 1H), 4.80 (dt, J = 9.3, 4.5 Hz, 1H), 2.64 – 2.54 (m, 1H), 2.27 (dq, J = 13.3, 6.8 Hz, 1H), 1.22 (d, J = 7.0 Hz, 1H), 0.90 (d, J = 7.3 Hz, 1H).

^{13}C NMR (*126 MHz, CDCl$_3$*) δ 168.0, 167.8, 140.8, 140.1, 138.9, 134.1, 134.0, 132.2, 132.0, 130.8, 128.9, 128.9, 128.8, 127.5, 127.2, 127.1, 126.9, 126.4, 79.3, 73.6, 50.4, 38.4, 37.7, 14.9, 12.7.

IR (ATR, ν in cm^{-1}): 2392 (m), 1636 (s), 1602 (m), 1580 (m), 1505 (s), 1487 (s), 1344 (s), 1272 (m), 1109 (m), 1100 (m), 1041 (m), 1013 (m), 994 (m), 853 (m), 840 (m), 690 (s), 670 (s).

HRMS (EI) m/z calcd for $C_{33}H_{32}N_2O_3$ 504.2413 $[M]^+$, found 504.2394 $[M]^+$.

2.2.3 Tetrahydro-2H-pyran **3c**

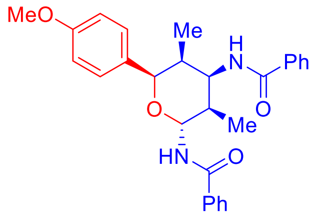

3c

Prepared according to TP1 from 4-methoxybenzaldehyde **1c** (30 µL, 0.25 mmol, 1.0 equiv.), $BF_3 \cdot OEt_2$ (1 M in DCM, 0.13 mL, 63 µmol, 0.25 equiv.), (*E*)-enamide **2a** (101 mg, 0.63 mmol, 2.5 equiv.) in a total of 2.5 mL dichloromethane. The reaction was allowed to warm to rt overnight. Column chromatography (*n*-hexane:EtOAc = 9:1→4:1→7:3→6:4) afforded the desired tetrahydropyran **3c** as a colorless foam (95 mg, 87%, isolated d.r. >98: 2; d.r. of the crude mixture >98:2 as determined by ^1H NMR analysis of the unpurified product after aqueous workup).

$\mathbf{R_f}$ (*n*-hexane:EtOAc = 6:4) 0.29.

m.p. 64-67 °C.

$\mathbf{^1H}$ **NMR** (500 MHz, *$CDCl_3$*-d) δ 7.84 – 7.81 (m, 2H), 7.60 – 7.56 (m, 2H), 7.53 – 7.47 (m, 2H), 7.41 (ddd, *J* = 14.5, 10.9, 7.8 Hz, 7H), 7.18 (d, *J* = 8.4 Hz, 1H), 6.91 (d, *J* = 8.7 Hz, 2H), 6.36 (d, *J* = 9.5 Hz, 1H), 5.87 (dd, *J* = 8.0, 6.4 Hz, 1H), 5.16 (d, *J* = 4.8 Hz, 1H), 4.76 (dt, *J* = 9.3, 4.5 Hz, 1H), 3.80 (s, *J* = 5.2 Hz, 3H), 2.56 – 2.47 (m, 1H), 2.27 – 2.18 (m, 1H), 1.20 (d, *J* = 7.0 Hz, 3H), 0.86 (d, *J* = 7.3 Hz, 3H).

$\mathbf{^{13}C}$ **NMR** (*126 MHz, $CDCl_3$*) δ 168.0, 167.8, 158.8, 134.2, 134.1, 132.1, 131.9, 131.9, 128.8, 128.7, 127.4, 127.2, 126.9, 114.0, 79.1, 73.6, 55.4, 50.5, 38.6, 37.8, 14.9, 12.7.

IR (ATR, ν in cm^{-1}): 3313 (m), 1640 (s), 1603 (w), 1579 (m), 1513 (s), 1486 (s), 1361 (w), 1272 (m), 1247 (s), 1177 (m), 1119 (m), 1100 (m), 1028 (s), 975 (m). 928 (w), 838 (m), 800 (m), 709 (s), 698 (s), 669 (m).

HRMS (EI) m/z calcd for $C_{28}H_{30}N_2O_4$ 458.2206 $[M]^+$, found 458.2226 $[M]^+$.

2.2.4 Tetrahydro-2H-pyran **3d**

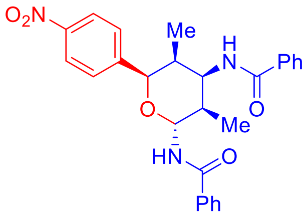

3d

Prepared according to TP1 from 4-nitrobenzaldehyde **1d** (38 mg, 0.25 mmol, 1.0 equiv.), $BF_3{\cdot}OEt_2$ (1 M in DCM, 0.13 mL, 63 µmol, 0.25 equiv.), (*E*)-enamide **2a** (101 mg, 0.63 mmol, 2.5 equiv.) in a total of 2.5 mL dichloromethane. The reaction was allowed to warm to rt overnight. Column chromatography (*n*-hexane:EtOAc = 9:1→4:1→7:3→6:4) afforded the desired tetrahydropyran **3d** as a colorless foam (106 mg, 90%, isolated d.r. >98: 2; d.r. of the crude mixture >98:2 as determined by ^{1}H NMR analysis of the unpurified product after aqueous workup).

R_f (*n*-hexane:EtOAc = 6:4) 0.13.

m.p. 72-76 °C.

^{1}H NMR (500 MHz, *CDCl$_3$*-d) δ 8.08 (d, J = 8.8 Hz, 2H), 7.78 (d, J = 7.3 Hz, 2H), 7.66 (dd, J = 11.7, 4.5 Hz, 3H), 7.51 – 7.36 (m, 6H), 7.34 – 7.18 (m, 4H), 6.39 (d, J = 9.2 Hz, 1H), 5.81 (dd, J = 7.5, 3.6 Hz, 1H), 5.21 (d, J = 4.0 Hz, 1H), 4.88 – 4.81 (m, 1H), 2.48 – 2.39 (m, 1H), 2.25 (dt, J = 14.8, 5.6 Hz, 1H), 1.21 (d, J = 7.2 Hz, 3H), 0.62 (d, J = 7.3 Hz, 3H).

^{13}C NMR (*126 MHz, CDCl$_3$*) δ 168.1, 167.9, 147.5, 147.0, 134.8, 133.8, 132.2, 129.0, 128.7, 128.6, 127.5, 126.9, 126.7, 123.5, 80.4, 72.1, 49.2, 37.5, 36.2, 15.2, 11.1.

IR (ATR, ν in cm^{-1}): 3314 (m), 1633 (s), 1603 (m), 1580 (m), 1515 (s), 1488 (s), 1344 (s), 1272 (m), 1182 (w), 1110 (m), 1100 (m), 1035 (m), 1014 (m), 993 (m), 853 (m), 841 (m), 694 (s), 659 (m).

HRMS (EI) m/z calcd for $C_{27}H_{27}N_3O_5$ 473.1951 [M]$^+$, found 473.1971 [M]$^+$.

2.2.5 Tetrahydro-2H-pyran **3e**

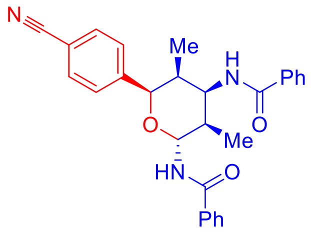

3e

Prepared according to TP1 from 4-cyanobenzaldehyde **1e** (33 mg, 0.25 mmol, 1.0 equiv.), $BF_3 \cdot OEt_2$ (1 M in DCM, 0.13 mL, 63 µmol, 0.25 equiv.), (*E*)-enamide **2a** (101 mg, 0.63 mmol, 2.5 equiv.) in a total of 2.5 mL dichloromethane. The reaction was allowed to warm to rt overnight. Column chromatography (*n*-hexane:EtOAc = 9:1→4:1→7:3→1:1) afforded the desired tetrahydropyran **3e** as a colorless solid (110 mg, 97%, isolated d.r. >98: 2; d.r. of the crude mixture >98: 2 as determined by ^{1}H NMR analysis of the unpurified product after aqueous workup). For a pure sample tetrahydropyran **3e** was further purified via preparative TLC **(***n*-hexane:EtOAc = 6:4).

R_f (*n*-hexane:EtOAc = 6:4) 0.11.

m.p. 93.8-102.3°C.

^{1}H NMR (*400 MHz, CDCl₃*) δ 7.90 – 7.80 (m, 2H), 7.81 – 7.75 (m, 1H), 7.75 – 7.67 (m, 2H), 7.60 (d, J = 8.4 Hz, 2H), 7.55 – 7.52 (m, 1H), 7.47 (ddd, J = 10.1, 7.3, 3.0 Hz, 5H), 7.40 (dd, J = 8.4, 6.9 Hz, 2H), 6.40 (d, J = 9.1 Hz, 1H), 5.87 (dd, J = 7.8, 4.0 Hz, 1H), 5.23 (d, J = 4.2 Hz, 1H), 4.90 (dt, J = 9.6, 4.9 Hz, 1H), 2.53 (dt, J = 7.5, 5.0 Hz, 1H), 2.29 (dt, J = 7.9, 4.1 Hz, 1H), 1.28 (d, J = 7.1 Hz, 3H), 0.71 (d, J = 7.3 Hz, 3H).

^{13}C NMR (*101 MHz, $CDCl_3$*) δ 167.9, 167.8, 145.4, 134.1, 133.8, 132.2, 132.2, 132.2, 129.0, 128.7, 128.7, 127.5, 126.9, 126.7, 118.9, 111.0, 80.2, 72.3, 49.3, 37.6, 36.5, 15.2, 11.3.

IR (ATR, ν in cm^{-1}): 3313 (m), 2970 (m), 2929 (m), 2230 (m), 1640 (s), 1605 (m), 1579 (m), 1520 (s), 1488 (s), 1363 (w), 1274 (m), 1183 (w), 1123 (m), 1100 (m), 1029 (s), 1001 (s), 832 (m), 802 (w) 765 (w), 711 (s), 690 (s).

HRMS (EI) m/z calcd for $C_{28}H_{27}N_3O_3$ 453.2052 $[M]^+$, found 453.2056 $[M+H]^+$.

2.2.6 Tetrahydro-2H-pyran **3f**

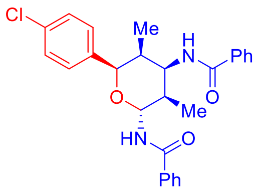

3f

Prepared according to TP1 from 4-chlorobenzaldehyde **1f** (35 mg, 0.25 mmol, 1.0 equiv.), $BF_3 \cdot OEt_2$ (1 M in DCM, 0.13 mL, 63 µmol, 0.25 equiv.), (*E*)-enamide **2a** (101 mg, 0.63 mmol, 2.5 equiv.) in a total of 2.5 mL dichloromethane. The reaction was allowed to warm to rt overnight. Column chromatography (*n*-hexane:EtOAc = 9:1→4:1→7:3→6:4) afforded the desired tetrahydropyran **3f** as a colorless foam (103 mg, 88%, isolated d.r. >98: 2; d.r. of the crude mixture >98:2 as determined by ^{1}H NMR analysis of the unpurified product after aqueous workup).

$\mathbf{R_f}$ (*n*-hexane:EtOAc = 6:4) 0.30.

m.p. 74-78 °C.

^{1}H NMR (400 MHz, CDCl3) δ 7.80 (d, J = 7.3 Hz, 2H), 7.63 – 7.60 (m, 2H), 7.53 – 7.38 (m, 5H), 7.36 – 7.24 (m, 6H), 6.37 (d, J = 9.4 Hz, 1H), 5.81 (dd, J = 7.9, 5.3 Hz, 1H), 5.15 (d, J = 4.7 Hz, 1H), 4.78 (dt, J = 9.4, 4.8 Hz, 1H), 2.48 – 2.37 (m, 1H), 2.30 – 2.21 (m, 1H), 1.19 (d, J = 7.1 Hz, 3H), 0.71 (d, J = 7.3 Hz, 3H).

^{13}C NMR (*126 MHz, $CDCl_3$*) δ 168.1, 167.8, 138.4, 134.1, 133.9, 132.8, 132.1, 132.0, 128.9, 128.6, 128.5, 127.5, 127.3, 126.9, 79.8, 72.6, 49.9, 38.0, 36.8, 15.0, 11.9.

IR (ATR, ν in cm^{-1}): 3313 (m), 1636 (s), 1603 (m), 1580 (m), 1517 (s), 1486 (s), 1303 (m), 1274 (m), 1114 (m), 1088 (m), 1041 (m), 1013 (m), 993 (m), 837 (m), 801 (m), 710 (s), 695 (s), 668 (s).

HRMS (EI) m/z calcd for $C_{27}H_{27}ClN_2O_3$ 462.1710 $[M]^+$, found 462.1691 $[M]^+$.

2.2.7 Tetrahydro-2H-pyran **3g**

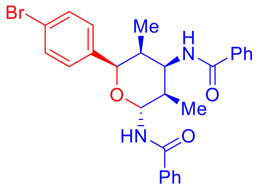

3g

Prepared according to TP1 from 4-bromobenzaldehyde **1g** (46 mg, 0.25 mmol, 1.0 equiv.), $BF_3 \cdot OEt_2$ (1 M in DCM, 0.13 mL, 63 µmol, 0.25 equiv.), (*E*)-enamide **2a** (101 mg, 0.63 mmol, 2.5 equiv.) in a total of 2.5 mL dichloromethane. The reaction was allowed to warm to rt overnight. Column chromatography (*n*-hexane:EtOAc = 9:1→4:1→7:3→1:1) afforded the desired tetrahydropyran **3g** as a colorless foam (113 mg, 89%, isolated d.r. >98: 2, d.r. of the crude mixture >98:2 as determined by ^{1}H NMR analysis of the unpurified product after aqueous workup)..

R_f (*n*-hexane:EtOAc = 6:4) 0.14.

m.p. 179 - 188 °C.

^{1}H NMR (*400 MHz, CDCl$_3$*) δ 7.85 – 7.78 (m, 2H), 7.69 – 7.60 (m, 2H), 7.57 – 7.49 (m, 2H), 7.49 – 7.35 (m, 6H), 7.31 (d, J = 8.2 Hz, 1H), 7.27 (d, J = 8.2 Hz, 2H), 6.35 (d, J = 9.4 Hz, 1H), 5.84 (dd, J = 8.1, 5.1 Hz, 1H), 5.14 (d, J = 4.6 Hz, 1H), 4.80 (dt, J = 9.5, 4.7 Hz, 1H), 2.49 (dt, J = 7.5, 5.0 Hz, 1H), 2.25 (dtd, J = 11.7, 7.0, 4.6 Hz, 1H), 1.22 (d, J = 7.1 Hz, 3H), 0.77 (d, J = 7.3 Hz, 3H).

^{13}C NMR (*101 MHz, CDCl$_3$*) δ 168.0, 167.8, 138.9, 134.1, 133.9, 132.2, 132.0, 131.5, 128.9, 128.7, 127.7, 127.4, 126.9, 121.0, 79.7, 72.8, 49.9, 38.0, 37.1, 15.0, 12.0.

IR (ATR, ν in cm^{-1}): 3312 (w), 2970 (w), 1646 (s), 1602 (m), 1580 (m), 1516 (s), 1486 (s), 1360 (m), 1271 (m), 1181 (w),1120 (m) 1097 (m), 1072 (m), 1039 (m), 991 (s), 836 (m) 799 (m), 780 (w), 707 (s), 690 (s), 669 (s).

HRMS (MALDI) m/z calcd for $C_{27}H_{27}BrN_2O_3$ 506.1205 [M]$^+$, found 506.1210 [M]$^+$.

2.2.8 Tetrahydro-2H-pyran **3h**

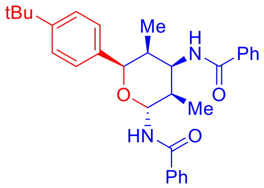

3h

Prepared according to TP1 from 4-(*tert*-butyl)benzaldehyde **3h** (42 µL, 0.25 mmol, 1.0 equiv.), BF_3·OEt_2 (1 M in DCM, 0.13 mL, 63 µmol, 0.25 equiv.), (*E*)-enamide **2a** (101 mg, 0.63 mmol, 2.5 equiv.) in a total of 2.5 mL dichloromethane. The reaction was allowed to warm to rt overnight. Column chromatography (*n*-hexane:EtOAc = 9:1→4:1→7:3→6:4) afforded the desired tetrahydropyran **3h** as a colorless foam (93 mg, 77%, isolated d.r. > 98:2; d.r.of the crude mixture 92:8 as determined by ^{1}H NMR analysis of the unpurified product after aqueous workup).

$\mathbf{R_f}$ (*n*-hexane:EtOAc = 6:4) 0.30.

m.p. 68-72 °C.

^{1}H NMR (400 MHz, *$CDCl_3$*) δ 7.82 (d, J = 7.4 Hz, 2H), 7.58 (d, J = 7.3 Hz, 2H), 7.49 (dd, J = 14.5, 7.3 Hz, 2H), 7.42 – 7.30 (m, 8H), 6.37 (d, J = 9.6 Hz, 2H), 5.90 – 5.84 (m, 1H), 5.19 (d, J = 5.1 Hz, 1H), 4.74 (dt, J = 9.2, 4.4 Hz, 1H), 2.59 – 2.49 (m, 1H), 2.29 – 2.19 (m, 1H), 1.31 (s, J = 7.5 Hz, 9H), 1.17 (d, J = 7.0 Hz, 3H), 0.87 (d, J = 7.3 Hz, 3H).

^{13}C NMR (*126 MHz, $CDCl_3$*) δ 168.0, 167.8, 150.1, 136.8, 134.2, 134.0, 132.0, 131.8, 128.8, 128.6, 128.6, 127.4, 126.9, 125.7, 125.4, 79.0, 73.8, 65.3, 50.8, 38.4, 37.8, 34.6, 31.5, 14.8, 13.0.

IR (ATR, ν in cm^{-1}): 3332 (m), 2963 (m), 1639 (s), 1603 (m), 1580 (m), 1515 (s), 1485 (s), 1362 (m), 1329 (m), 1270 (s), 1184 (m), 1100 (m), 1075 (m), 975 (m), 838 (m), 798 (m), 709 (s), 695 (s), 671 (m).

HRMS (EI) m/z calcd for $C_{31}H_{36}N_2O_3$ 484.2726 $[M]^+$, found 484.2744 $[M]^+$.

2.2.9 Tetrahydro-2H-pyran **3i**

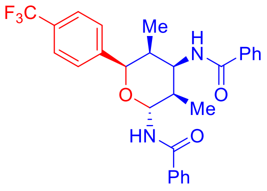

3i

Prepared according to TP1 from 4-(trifluoromethyl)benzaldehyde **1i** (34 µL, 0.25 mmol, 1.0 equiv.), BF_3·OEt_2 (1 M in DCM, 0.13 mL, 63 µmol, 0.25 equiv.), (*E*)-enamide **2a** (101 mg, 0.63 mmol, 2.5 equiv.) in a total of 2.5 mL dichloromethane. The reaction was allowed to warm to rt overnight. Column chromatography (*n*-hexane:EtOAc = 9:1→4:1→7:3→6:4) afforded the desired tetrahydropyran **3i** as a colorless foam (103 mg, 88%, isolated d.r. >98:2; d.r. of the crude mixture 94:6 as determined by ^{1}H NMR analysis of the unpurified product after aqueous workup).

R_f (*n*-hexane:EtOAc = 6:4) 0.34.

m.p. 162-197 °C.

^{1}H NMR (*400 MHz, $CDCl_3$*) δ 7.83 (d, *J* = 7.5 Hz, 2H), 7.66 (d, *J* = 7.3 Hz, 2H), 7.61 – 7.54 (m, 3H), 7.52 (d, *J* = 7.2 Hz, 1H), 7.50 – 7.34 (m, 8H), 6.39 (d, *J* = 9.3 Hz, 1H), 5.87 (dd, *J* = 7.6, 4.7 Hz, 1H), 5.25 (d, *J* = 4.1 Hz, 1H), 4.87 (dt, *J* = 9.4, 4.7 Hz, 1H), 2.56 – 2.47 (m, 1H), 2.36 – 2.25 (m, 1H), 1.24 (d, *J* = 7.1 Hz, 3H), 0.73 (d, *J* = 7.3 Hz, 3H).

^{13}C NMR (*101 MHz, Chloroform-d*) δ 168.1, 167.9, 144.0, 134.2, 133.9, 132.1, 132.1, 130.0 – 128.7 (m), 128.9, 128.7, 127.5, 126.9, 126.2, 125.3 (q, *J* = 3.6 Hz), 80.0, 72.7 , 49.7, 37.9, 36.8, 15.1, 11.7.

^{19}F NMR (*376 MHz, $CDCl_3$*) δ -62.42.

IR (ATR, ν in cm^{-1}): 3276.7 (m), 1734 (m), 1657 (m), 1629 (s), 1580 (m), 1528 (s), 1490 (m), 1323 (s), 1293 (m), 1240 (m), 1164 (m), 1121 (s), 1067 (m), 1045 (s), 1018 (s), 934 (m), 843 (m), 786 (m), 695 (s), 668 (m).

HRMS (EI) m/z calcd for $C_{28}H_{27}F_3N_2O_3$ 496.1974 $[M]^+$, found 496.1993 $[M]^+$.

2.2.10 Tetrahydro-2H-pyran **3j**

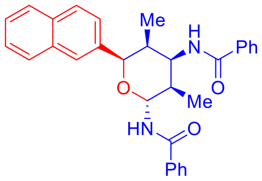

3j

Prepared according to TP1 from 2-naphthaldehyde **1j** (39 mg, 0.25 mmol, 1.0 equiv.), $BF_3 \cdot OEt_2$ (1 M in DCM, 0.13 mL, 63 µmol, 0.25 equiv.), (*E*)-enamide **2a** (101 mg, 0.63 mmol, 2.5 equiv.) in a total of 2.5 mL dichloromethane. The reaction was allowed to warm to rt overnight. Column chromatography (*n*-hexane:EtOAc = 9:1→4:1→7:3→6:4) afforded the desired tetrahydropyran **3j** as a colorless foam (76.8 mg, 64%, isolated d.r. >98:2; d.r. of the crude mixture >98:2 as determined by ^{1}H NMR analysis of the unpurified product after aqueous workup).

R$_f$ (*n*-hexane:EtOAc = 6:4) 0.29.

m.p. 67-71 °C.

^{1}H NMR (400 MHz, *CDCl$_3$*) δ 8.03 (s, 1H), 7.88 – 7.77 (m, 6H), 7.56 (d, *J* = 7.7 Hz, 2H), 7.52 – 7.37 (m, 10H), 7.32 (t, *J* = 7.7 Hz, 2H), 6.42 (d, *J* = 9.4 Hz, 1H), 5.97 (dd, *J* = 7.8, 5.9 Hz, 1H), 5.37 (d, *J* = 4.7 Hz, 1H), 4.84 (dt, *J* = 9.4, 4.8 Hz, 1H), 2.67 – 2.59 (m, 1H), 2.35 – 2.25 (m, 1H), 1.24 (d, *J* = 7.0 Hz, 3H), 0.83 (d, *J* = 7.3 Hz, 3H).

^{13}C NMR (*126 MHz, CDCl$_3$*) δ 168.1, 167.8, 137.3, 134.2, 134.1, 133.5, 132.6, 132.1, 131.9, 128.8, 128.7, 128.3, 128.0, 127.7, 127.5, 126.9, 126.3, 126.0, 124.6, 124.1, 79.6, 73.6, 50.4, 38.3, 37.4, 15.0, 12.5.

IR (ATR, ν in cm^{-1}): 2390 (m), 1639 (s), 1603 (m), 1579 (m), 1514 (s), 1485 (s), 1323 (m), 1272 (s), 1101 (m), 1041 (m), 904 (m), 853 (m), 806 (m), 709 (s), 697 (s), 670 (m), 655 (m).

HRMS (EI) m/z calcd for $C_{31}H_{30}N_2O_3$ 478.2256 [M]$^+$, found 478.2265 [M]$^+$.

2.2.11 Tetrahydro-2H-pyran **3k**

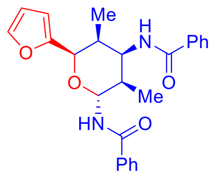

3k

Prepared according to TP1 from furan-2-carbaldehyde **1k** (21 µL, 0.25 mmol, 1.0 equiv.), $BF_3{\cdot}OEt_2$ (1 M in DCM, 0.13 mL, 63 µmol, 0.25 equiv.), (*E*)-enamide **2a** (101 mg, 0.63 mmol, 2.5 equiv.) in a total of 2.5 mL dichloromethane. The reaction was allowed to warm to rt overnight. Column chromatography (*n*-hexane:EtOAc = 9:1→4:1→7:3→1:1) afforded the desired tetrahydropyran **3k** as a colorless foam (89.3 mg, 85%, isolated d.r. >98:2; d.r. of the crude mixture >98:2 as determined by ^{1}H NMR analysis of the unpurified product after aqueous workup).

R_f (*n*-hexane:EtOAc = 6:4) 0.16.
m.p. 120-136 °C.
^{1}H NMR (400 MHz, *$CDCl_3$*) δ 7.83 – 7.78 (m, 2H), 7.67 – 7.63 (m, 2H), 7.53 – 7.45 (m, 3H), 7.44 – 7.36 (m, 4H), 7.17 (d, J = 10.5 Hz, 1H), 6.88 (d, J = 9.3 Hz, 1H), 6.66 (d, J = 3.3 Hz, 1H), 6.47 (dd, J = 3.2, 1.9 Hz, 1H), 5.92 (t, J = 9.9 Hz, 1H), 4.99 (d, J = 6.6 Hz, 1H), 4.69 (dt, J = 10.4, 4.1 Hz, 1H), 2.60 – 2.51 (m, 1H), 2.19 – 2.09 (m, 1H), 1.03 (d, J = 6.9 Hz, 3H), 0.93 (d, J = 7.2 Hz, 3H).
^{13}C NMR (*126 MHz, $CDCl_3$*) δ 168.2, 167.6, 153.2, 142.8, 134.5, 133.8, 132.1, 131.7, 128.7, 128.7, 127.3, 126.9, 111.2, 110.6, 76.0, 73.2, 51.0, 40.0, 36.9, 13.7, 13.4.
IR (ATR, ν in cm^{-1}): 3313 (m), 2961 (s), 1644 (s), 1603 (m), 1580 (m), 1514 (s), 1485 (s), 1361 (m), 1270 (m), 1186 (m), 1100 (m), 1041 (m), 974 (m), 841 (m), 805 (m), 709 (s), 699 (s), 669 (m).
HRMS (EI) m/z calcd for $C_{25}H_{26}N_2O_4$ 418.1893 $[M]^+$, found 418.1906 $[M]^+$.

2.2.12 Tetrahydro-2H-pyran **3l**

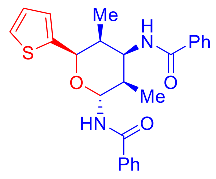

3l

Prepared according to TP1 from Aldehyde **1l** (56 µL, 0.5 mmol, 1.0 equiv.), $BF_3 \cdot OEt_2$ (1 M in DCM, 0.25 mL, 125 µmol, 0.25 equiv.), (*E*)-enamide **2a** (201 mg, 0.63 mmol, 2.5 equiv.) in a total of 5.0 mL dichloromethane. The reaction was allowed to warm to rt overnight. Column chromatography (*n*-hexane: EtOAc = 4:1→7:3→1:1) afforded the desired tetrahydropyran **3l** as a colorless foam (211 mg, 97%, 0.48 mmol isolated d.r. >98:2; d.r. of the crude mixture >98:2 as determined by ^{1}H NMR analysis of the unpurified product after aqueous workup).

R_f (*n*-hexane:EtOAc = 6:4) 0.05.

m.p. 117 – 126 °C.

^{1}H NMR (*400 MHz, CDCl$_3$*) δ 7.85 (d, *J* = 7.3 Hz, 2H), 7.74 (d, *J* = 3.4 Hz, 1H), 7.55 (t, *J* = 7.3 Hz, 1H), 7.50 – 7.42 (m, 3H), 7.38 (d, *J* = 5.0 Hz, 1H), 7.35 – 7.28 (m, 4H), 7.19 (dd, *J* = 4.9, 3.7 Hz, 1H), 6.68 (d, *J* = 9.3 Hz, 1H), 6.29 (d, *J* = 10.1 Hz, 1H), 5.97 (t, *J* = 9.5 Hz, 1H), 5.36 (d, *J* = 5.5 Hz, 1H), 4.67 (dt, *J* = 9.9, 3.5 Hz, 1H), 2.74 – 2.60 (m, 1H), 2.15 (ddd, *J* = 12.6, 6.5, 3.0 Hz, 1H), 1.32 (d, *J* = 7.4 Hz, 3H), 1.04 (d, *J* = 6.9 Hz, 3H).

^{13}C NMR (*101 MHz, CDCl$_3$*) δ 168.0, 167.5, 142.1, 133.8, 133.6, 132.3, 131.8, 128.9, 128.6, 127.6, 127.3, 126.8, 126.1, 125.8, 76.0, 75.8, 51.9, 40.2, 39.0, 14.3, 13.8.

^{13}C NMR (*101 MHz, DMSO*) δ 167.1, 167.0, 143.6, 134.6, 134.1, 131.6, 131.2, 128.3, 128.2, 127.7, 127.3, 126.8, 124.7, 124.0, 78.2, 71.8, 49.6, 37.9, 35.8, 14.6, 12.0.

IR (ATR, ν in cm^{-1}): 3445 (m), 3234 (m), 3067 (w), 2963 (m), 1656 (s), 1579 (s), 1528 (s), 1486 (s), 1307 (m), 1279 (m), 1248 (w), 1173 (w), 1103 (w), 1070 (m), 1028 (m), 1016 (m), 966 (m), 938 (w), 839 (w), 705 (s).

HRMS (EI) m/z calcd for $C_{25}H_{26}N_2O_3S$ 434.1664 [M]$^+$, found 434.1667 [M]$^+$.

2.2.13 Tetrahydro-2H-pyran **3m**

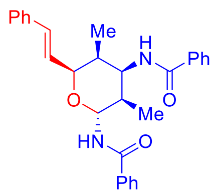

3m

Prepared according to TP1 from *trans*-cinnamaldehyde **1m** (32 µL, 0.25 mmol, 1.0 equiv.), BF_3·OEt_2 (1 M in DCM, 0.28 mL, 275 µmol, 1.1 equiv.), (*E*)-enamide **2a** (*E*)-enamide **2a** (101 mg, 0.63 mmol, 2.5 equiv.) in a total of 2.5 mL dichloromethane. The reaction was allowed to warm to -50 ° over a period of 3 h. Column chromatography (*n*-hexane:EtOAc = 4:1→7:3→1:1) afforded the desired tetrahydropyran **3m** as a colorless solid (99 mg, 85%, isolated d.r. >98: 2; d.r. of the crude mixture >98: 2 as determined by ^{1}H NMR analysis of the unpurified product after aqueous workup).

R_f (*n*-hexane:EtOAc = 6:4) 0.1.

m.p. 165-168 °C.

^{1}H NMR (*400 MHz, $CDCl_3$*) δ 7.90 – 7.84 (m, 2H), 7.84 – 7.78 (m, 1H), 7.66 – 7.61 (m, 2H), 7.55 (dq, J = 5.9, 1.6 Hz, 3H), 7.47 (dd, J = 8.2, 6.7 Hz, 2H), 7.42 – 7.30 (m, 4H), 7.06 (t, J = 7.8 Hz, 2H), 6.66 (d, J = 10.2 Hz, 1H), 6.61 (d, J = 9.5 Hz, 1H), 6.50 (dd, J = 16.5, 2.6 Hz, 1H), 5.78 (t, J = 10.0 Hz, 1H), 4.79 – 4.72 (m, 1H), 4.64 (dt, J = 10.5, 3.5 Hz, 1H), 2.52 (td, J = 6.7, 3.3 Hz, 1H), 2.09 (ddd, J = 10.5, 7.0, 3.7 Hz, 1H), 1.15 (d, J = 7.2 Hz, 3H), 0.99 (d, J = 6.8 Hz, 3H).

^{13}C NMR (*101 MHz, $CDCl_3$*) δ 168.3, 167.2, 136.4, 134.1, 133.3, 132.2, 131.8, 131.6, 129.0, 128.0, 128.7, 128.3, 127.3, 127.0, 126.8, 124.2, 76.3, 74.1, 52.4, 39.9, 38.7, 14.0, 13.7.

IR (ATR, ν in cm^{-1}): 3432 (w), 3277 (m), 1667 (s), 1652 (s), 1601 (w), 1519 (s), 1485 (s), 1448 (s), 1392 (m), 1364 (m), 1316 (m), 1303 (m), 1273 (m), 1204 (w), 1130 (m), 1071 (s), 1025 (w), 983 (m), 940 (w), 826 (w), 772 (m), 736 (m), 695 (s).

HRMS m/z calcd for $C_{29}H_{30}N_2O_3$ 454.2256 $[M]^+$, found 454.2262 $[M]^+$.

2.2.14 Tetrahydro-2H-pyran **3n**

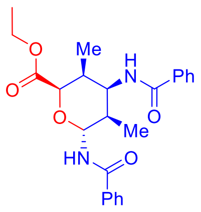

3n

Prepared according to TP1 from ethyl glyoxalate **1n** (51 µL, 0.25 mmol, 1.0 equiv., 50% in toluene), $BF_3{\cdot}OEt_2$ (1 M in DCM, 0.28 mL, 275 µmol, 1.1 equiv.), (*E)*-enamide **2a** (*E)*-enamide **2a** (101 mg, 0.63 mmol, 2.5 equiv.) in a total of 2.5 mL dichloromethane. The reaction was allowed to warm to -50 ° over a period of 2h. Column chromatography (*n*-hexane:EtOAc = 9:1→4:1→7:3→1:1) afforded the desired tetrahydropyran **3n** as a colorless foam (88 mg, 83%, isolated d.r. >98: 2; d.r. of the crude mixture 95:5 as determined by ^{1}H NMR analysis of the unpurified product after aqueous workup).

R_f (*n*-hexane:EtOAc = 6:4) 0.09.

m.p. 72-78 °C.

^{1}H NMR (*400 MHz, CDCl$_3$*) δ 8.07 (d, J = 10.3 Hz, 1H), 7.98 – 7.90 (m, 2H), 7.86 – 7.75 (m, 2H), 7.53 – 7.41 (m, 6H), 6.58 (d, J = 9.5 Hz, 1H), 6.08 (t, J = 10.0 Hz, 1H), 4.70 (dt, J = 10.4, 4.2 Hz, 1H), 4.46 (d, J = 6.8 Hz, 1H), 4.35 – 4.16 (m, 2H), 2.41 (td, J = 7.1, 4.5 Hz, 1H), 2.22 – 1.98 (m, 1H), 1.30 (t, J = 7.1 Hz, 3H), 1.03 (d, J = 6.8 Hz, 3H), 0.96 (d, J = 7.1 Hz, 3H).

^{13}C NMR (*101 MHz, CDCl$_3$*) δ 173.7, 167.7, 167.3, 133.8, 133.8, 132.0, 131.6, 128.6, 128.6, 127.4, 127.2, 127.1, 75.8, 61.9, 50.6, 39.7, 34.6, 14.1, 13.3, 12.6.

IR (ATR, ν in cm^{-1}): 3327 (w), 2970 (m), 2934 (m), 1717 (m), 1645 (s), 1604 (w), 1580 (m), 1526 (s), 1489 (s), 1449 (m), 1382 (m), 1297 (m), 1202 (m), 1157 (m), 1128 (w), 1084 (s), 1028 (m), 975 (m), 938 (w), 891 (w), 802 (m), 692 (s), 668 (s).

HRMS (MALDI) m/z calcd for $C_{24}H_{28}N_2O_5$ 424.2004 $[M]^+$, found 424.1998 $[M]^+$.

2.2.15 Tetrahydro-2H-pyran **3o**

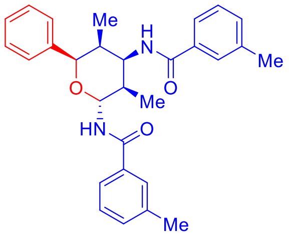

3o

Prepared according to TP1 from benzaldehyde **1a** (25 µL, 0.25 mmol, 1.0 equiv.), $BF_3 \cdot OEt_2$ (1 M in DCM, 0.13 mL, 63 µmol, 0.25 equiv.), (*E*)-enamide **2b** (110 mg, 0.63 mmol, 2.5 equiv.) in a total of 2.5 mL dichloromethane. The reaction was allowed to warm to rt overnight. Column chromatography (*n*-hexane:EtOAc = 9:1→4:1→7:3→1:1) afforded the desired tetrahydropyran **3o** as a colorless foam (104 mg, 77%, isolated d.r. >98: 2; d.r. of the crude mixture 94:6 as determined by ^{1}H NMR analysis of the unpurified product after aqueous workup).

R_f (*n*-hexane:EtOAc = 6:4) 0.32.

m.p. 116-120 °C.

^{1}H NMR (*400 MHz, $CDCl_3$*) δ 7.63 (s, 1H), 7.62 – 7.57 (m, 1H), 7.45 (d, J = 7.3 Hz, 2H), 7.40 – 7.27 (m, 9H), 7.11 (d, J = 8.4 Hz, 1H), 6.34 (d, J = 9.5 Hz, 1H), 5.87 (dd, J = 8.4, 6.0 Hz, 1H), 5.20 (d, J = 4.9 Hz, 1H), 4.78 (dt, J = 9.4, 4.6 Hz, 1H), 2.68 – 2.50 (m, 1H), 2.39 (s, 3H), 2.38 (s, 3H), 2.30 – 2.16 (m, 1H), 1.21 (d, J = 7.0 Hz, 3H), 0.83 (d, J = 7.3 Hz, 3H).

^{13}C NMR (*101 MHz, $CDCl_3$*) δ 168.1, 168.0, 139.9, 138.7, 138.6, 134.2, 134.0, 132.9, 132.6, 128.7, 128.6, 128.5, 128.1, 127.5, 127.2, 126.0, 124.4, 124.0, 79.3, 73.6, 50.3, 38.3, 37.7, 21.5, 21.5, 14.9, 12.6.

IR (ATR, ν in cm^{-1}): 3297 (w), 2967 (w), 2923 (w), 1636 (m), 1606 (w) 1586 (m), 1514 (s), 1483 (s), 1453 (m), 1378 (m), 1279 (m), 1212 (m), 1088 (m), 1039 (m), 996 (m), 918 (w), 807 (m), 740 8s), 693 (s).

HRMS (MALDI) m/z calcd for $C_{29}H_{32}N_2O_3$ 456.2413 [M+H]$^+$, found 456.2417 [M+H]$^+$.

2.2.16 Tetrahydro-2H-pyran **3p**

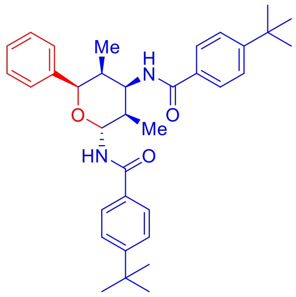

3p

Prepared according to TP1 from benzaldehyde **1a** (25 µL, 0.25 mmol, 1.0 equiv.), $BF_3·OEt_2$ (1 M in DCM, 0.13 mL, 63 µmol, 0.25 equiv.), (*E*)-enamide **2c** (136 mg, 0.63 mmol, 2.5 equiv.) in a total of 2.5 mL dichloromethane. The reaction was allowed to warm to rt overnight. Column chromatography (*n*-hexane:EtOAc = 9:1→4:1→7:3→1:1) afforded the desired tetrahydropyran **3p** as a colorless foam (109 mg, 81%, isolated d.r. = >98: 2; d.r. of the crude mixture >98: 2 as determined by ^1H NMR analysis of the unpurified product after aqueous workup).

R_f (*n*-hexane:EtOAc = 7:3) 0.22.

m.p. 101-107 °C.

^1H NMR (*500 MHz, $CDCl_3$*) δ 7.69 (d, J = 8.5 Hz, 2H), 7.50 (d, J = 8.5 Hz, 2H), 7.37 (d, J = 8.3 Hz, 5H), 7.28 (t, J = 7.7 Hz, 2H), 7.19 (s, 2H), 6.98 (d, J = 8.2 Hz, 1H), 6.29 (d, J = 9.6 Hz, 1H), 5.80 (dd, J = 8.3, 5.6 Hz, 1H), 5.12 (d, J = 4.8 Hz, 1H), 4.72 (dt, J = 9.5, 4.9 Hz, 1H), 2.61 – 2.41 (m, 1H), 2.23 – 2.10 (m, 1H), 1.27 (s, 9H), 1.25 (s, 9H), 1.15 (d, J = 7.0 Hz, 3H), 0.73 (d, J = 7.3 Hz, 3H).

^{13}C NMR (*126 MHz, $CDCl_3$*) δ 167.6, 167.6, 155.6, 155.3, 139.8, 131.2, 131.0, 128.4, 127.1, 127.0, 126.9, 126.7, 125.9, 125.6, 79.3, 73.3, 50.0, 38.2, 37.5, 35.0, 34.9, 31.2, 31.1, 14.8, 12.2.

IR (ATR, ν in cm^{-1}): 3313 (w), 2961 (s), 2922 (s), 1645 (s), 1612 (s), 1530 (s), 1498 (s), 1460 (m), 1364 (m), 1314 (w), 1270 (m), 1117 (m), 1016 (s), 850 (s), 772 (m), 746 (w), 700 (s), 673 (m).

HRMS (MALDI) m/z calcd for $C_{35}H_{44}N_2O_3$ 540.3352 $[M]^+$, found 540.3363 $[M]^+$.

2.2.17 Tetrahydro-2H-pyran **3q**

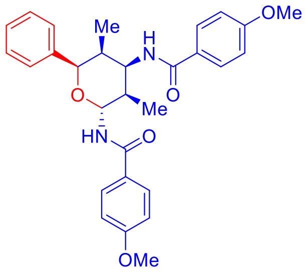

3q

Prepared according to TP1 from benzaldehyde **1a** (25 µL, 0.25 mmol, 1.0 equiv.), $BF_3 \cdot OEt_2$ (1 M in DCM, 0.13 mL, 63 µmol, 0.25 equiv.), (*E*)-enamide **2d** (120 mg, 0.63 mmol, 2.5 equiv.) in a total of 2.5 mL dichloromethane. The reaction was allowed to warm to rt overnight. Column chromatography (*n*-hexane:EtOAc = 9:1→4:1→7:3→1:1) afforded the desired tetrahydropyran **3q** as a colorless foam (102 mg, 84%, isolated d.r. >98: 2; d.r. of the crude mixture >98: 2 as determined by ^{1}H NMR analysis of the unpurified product after aqueous workup).

R_f (*n*-hexane:EtOAc = 1:1) 0.28.

m.p. 151-153 °C.

^{1}H NMR (*400 MHz, $CDCl_3$*) δ 7.79 (d, *J* = 8.8 Hz, 2H), 7.56 (d, *J* = 8.8 Hz, 2H), 7.45 (d, *J* = 7.4 Hz, 2H), 7.40 – 7.27 (m, 3H), 6.96 (d, *J* = 8.5 Hz, 1H), 6.90 (dd, *J* = 8.8, 3.7 Hz, 4H), 6.26 (d, *J* = 9.5 Hz, 1H), 5.87 (dd, *J* = 8.3, 5.9 Hz, 1H), 5.19 (d, *J* = 4.9 Hz, 1H), 4.76 (dt, *J* = 9.4, 4.6 Hz, 1H), 3.85 (d, *J* = 5.8 Hz, 6H), 2.56 (dt, *J* = 7.5, 5.2 Hz, 1H), 2.21 (tt, *J* = 10.9, 5.5 Hz, 1H), 1.21 (d, *J* = 7.0 Hz, 3H), 0.82 (d, *J* = 7.3 Hz, 3H).

^{13}C NMR (*101 MHz, $CDCl_3$*) δ 167.2, 162.7, 162.5, 140.0, 129.3, 128.8, 128.7, 128.5, 127.1, 126.4, 126.2, 126.0, 114.0, 113.9, 79.3, 73.6, 55.6, 55.6, 50.3, 38.4, 37.8, 14.9, 12.6.

IR (ATR, ν in cm^{-1}): 3305 (w), 2964 (w), 2932 (m), 1626 (s), 1605 (s), 1578 (m), 1533 (m), 1499 (s), 1381 (m), 1306 (m), 1251 (s), 1174 (m), 1111 (w), 1029 (m), 1018 (m), 840(m), 767 (m), 743 (m), 700(m).

HRMS (EI) m/z calcd for $C_{29}H_{32}N_2O_5$ 488.2311 $[M]^+$, found 488.2328 $[M]^+$.

2.2.18 Tetrahydro-2H-pyran **3r**

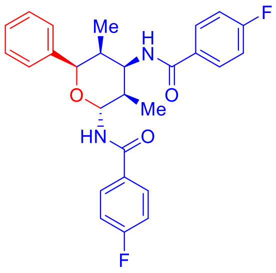

3r

Prepared according to TP1 from benzaldehyde **1a** (25 µL, 0.25 mmol, 1.0 equiv.), $BF_3{\cdot}OEt_2$ (1 M in DCM, 0.13 mL, 63 µmol, 0.25 equiv.), (*E*)-enamide **2e** (112 mg, 0.63 mmol, 2.5 equiv. in 2.5 mL DCM) in a total of 3.75 mL dichloromethane. The reaction was allowed to warm to rt overnight. Column chromatography (*n*-hexane:EtOAc = 9:1→4:1→7:3→1:1) afforded the desired tetrahydropyran **3r** as a colorless foam (82.4 mg, 71%, isolated d.r. >98: 2; d.r. of the crude mixture 95:5 as determined by ^{1}H NMR analysis of the unpurified product after aqueous workup).

R_f (*n*-hexane:EtOAc = 6:4) 0.14.

m.p. 166-174°C.

^{1}H NMR *(400 MHz, CDCl$_3$)* δ 8.10 – 7.74 (m, 2H), 7.71 – 7.55 (m, 2H), 7.46 (d, J = 7.3 Hz, 2H), 7.38 (t, J = 7.6 Hz, 3H), 7.29 (d, J = 7.3 Hz, 1H), 7.16 – 7.05 (m, 4H), 7.00 (d, J = 8.3 Hz, 1H), 6.27 (d, J = 9.5 Hz, 1H), 5.87 (dd, J = 8.3, 5.9 Hz, 1H), 5.19 (d, J = 4.9 Hz, 1H), 4.76 (dt, J = 9.4, 4.6 Hz, 1H), 2.57 (dt, J = 7.4, 5.1 Hz, 1H), 2.33 – 2.18 (m, 1H), 1.21 (d, J = 7.0 Hz, 3H), 0.85 (d, J = 7.3 Hz, 3H).

^{13}C NMR (101 MHz, *CDCl$_3$*) δ 166.8, 166.7, 139.7, 130.3 (d, *J* = 3.0 Hz), 130.1 (d, *J* = 2.9 Hz), 129.9, 129.8, 129.3, 129.2, 128.6, 127.3, 125.9, 116.0 (d, *J* = 2.7 Hz), 115.8 (d, *J* = 2.6 Hz), 79.3, 73.8, 50.6, 38.4, 37.8, 14.9, 12.7.

^{19}F NMR (376 MHz, *CDCl$_3$*) δ -106.96 – -107.18 (m), -107.44 – -107.62 (m).

IR (ATR, ν in cm^{-1}): 3306 (w), 2971 (w), 2930 (w), 1659 (m), 1630 (s), 1602 (s), 1535 (s), 1498 (s), 1381 (w), 1322 8w), 1285 (m), 1228 (s), 1185 (w), 1157 (m), 1039 (m), 1015 (m), 975 (w), 932 (w), 892 (w), 845 (s), 786 (w), 761 (s), 702 (m).

HRMS m/z calcd for $C_{27}H_{25}F_2N_2O_3$ 463.1833 [M-H]$^-$, found 463.1839 [M-H]$^-$.

2.2.19 Tetrahydro-2H-pyran **3s**

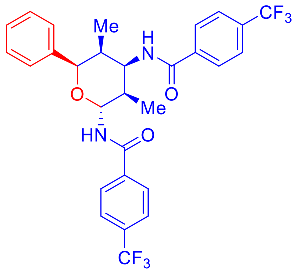

3s

Prepared according to TP1 from benzaldehyde **1a** (25 µL, 0.25 mmol, 1.0 equiv.), BF_3·OEt_2 (1 M in DCM, 0.13 mL, 63 µmol, 0.25 equiv.), (*E)*-enamide **2f** (143 mg, 0.63 mmol, 2.5 equiv. in 2.5 mL DCM) in a total of 3.75 mL dichloromethane. The reaction was allowed to warm to rt overnight. Column chromatography (*n*-hexane:EtOAc = 9:1→4:1→7:3→1:1) afforded the desired tetrahydropyran **3s** as a colorless foam (109 mg, 77%, isolated d.r. >98: 2; d.r. of the crude mixture 93:7 as determined by ^{1}H NMR analysis of the unpurified product after aqueous workup).

$\mathbf{R_f}$ (*n*-hexane:EtOAc = 6:4) 0.58.

m.p. 99-110 °C.

^{1}H NMR (*400 MHz, CDCl$_3$*) δ 7.90 (d, J = 8.2 Hz, 2H), 7.75 – 7.54 (m, 6H), 7.46 – 7.41 (m, 3H), 7.37 (t, J = 7.5 Hz, 2H), 7.29 (d, J = 7.3 Hz, 1H), 6.42 (d, J = 9.4 Hz, 1H), 5.90 (dd, J = 8.2, 5.9 Hz, 1H), 5.22 (d, J = 4.8 Hz, 1H), 4.82 (dt, J = 9.4, 4.6 Hz, 1H), 2.56 (dt, J = 7.8, 5.2 Hz, 1H), 2.29 (td, J = 6.7, 4.4 Hz, 1H), 1.22 (d, J = 7.0 Hz, 3H), 0.84 (d, J = 7.3 Hz, 3H).

^{13}C NMR (*101 MHz, CDCl$_3$*) δ 166.9, 166.6, 139.5, 137.3, 137.2, 133.9, 133.9, 133.6, 133.5, 128.7, 127.9, 127.4, 125.9, 125.9, 125.7, 125.7, 125.0, 122.3, 79.5, 73.8, 50.7, 38.3, 37.4, 14.9, 12.5.

^{19}F NMR (*376 MHz, CDCl$_3$*) δ -63.00, -63.01.

IR (ATR, ν in cm^{-1}): 3301 (w), 2975 (w), 1646 (w), 1581 (w), 1533 (m), 1502 (m), 1410 (w), 1323 (s), 1166 (m), 1123 (s), 1066 (s), 1016 (s), 856 (m), 770 (m), 743 (w), 702 (m), 671 (w).

HRMS (MALDI) m/z calcd for $C_{29}H_{26}F_6N_2O$ 564.1848 [M]$^+$, found 564.1832 [M]$^+$.

2.2.20 Tetrahydro-2H-pyran **3t**

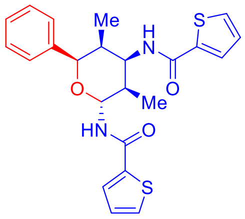

3t

Prepared according to TP1 from benzaldehyde **1a** (25 µL, 0.25 mmol, 1.0 equiv.), $BF_3{\cdot}OEt_2$ (1 M in DCM, 0.13 mL, 63 µmol, 0.25 equiv.), (*E*)-enamide **2g** (105 mg, 0.63 mmol, 2.5 equiv. in 2.5 mL DCM) in a total of 3.75 mL dichloromethane. The reaction was allowed to warm to rt overnight. Column chromatography (*n*-hexane:EtOAc = 9:1→4:1→7:3→1:1) afforded the desired tetrahydropyran **3t** as a colorless foam (77 mg, 60%, isolated d.r. >98: 2; d.r. of the crude mixture >98:2 as determined by ^{1}H NMR analysis of the unpurified product after aqueous workup).

R_f (*n*-hexane:EtOAc = 6:4) 0.15.

m.p. decomposition before melting (182.6 °C).

^{1}H NMR (*600 MHz, DMSO-d_6*) δ 9.12 (d, J = 8.3 Hz, 1H), 7.98 (ddd, J = 8.6, 3.8, 1.2 Hz, 2H), 7.79 (dd, J = 5.0, 1.1 Hz, 1H), 7.76 (dd, J = 5.0, 1.1 Hz, 1H), 7.74 (d, J = 8.8 Hz, 1H), 7.36 – 7.27 (m, 4H), 7.21 (tt, J = 6.7, 1.8 Hz, 1H), 7.19 – 7.11 (m, 2H), 5.69 (dd, J = 8.3, 5.3 Hz, 1H), 5.20 (d, J = 4.3 Hz, 1H), 4.87 (ddd, J = 8.9, 6.5, 4.7 Hz, 1H), 2.54 – 2.50 (m, 1H), 2.34 (dt, J = 7.3, 5.2 Hz, 1H), 1.12 (d, J = 7.1 Hz, 3H), 0.56 (d, J = 7.3 Hz, 3H).

^{13}C NMR (*151 MHz, DMSO-d_6*) δ 161.8, 161.5, 140.7, 139.6, 139.5, 131.7, 130.9, 129.3, 128.8, 127.9, 127.9, 127.8, 126.6, 125.8, 79.7, 71.7, 48.9, 37.0, 34.7, 15.1, 10.9.

IR (ATR, ν in cm^{-1}): 3347 (m) 3313 (m), 2969 (m), 1726 (m), 1650 (m), 1619 (s), 1531 (s), 1509 (s), 1420 (m), 1397 (w), 1356 (w), 1305 (m), 1289 (m), 1154 (m), 1126 (m), 1089 (w), 1078 (m), 1040 (s), 1017 (s), 957 (m), 924 (m), 860 (m), 819 (w), 795 (w), 774 (m), 749 (w), 737 (m), 700 (s).

HRMS (EI) m/z calcd for $C_{23}H_{24}N_2O_3S_2$ 440.1228 [M]$^+$, found 440.1237 [M]$^+$.

2.2.21 Tetrahydro-2H-pyran **3u**

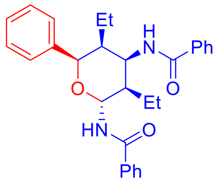

3u

Prepared according to TP1 from benzaldehyde **1a** (25 µL, 0.25 mmol, 1.0 equiv.), $BF_3{\cdot}OEt_2$ (1 M in DCM, 0.13 mL, 63 µmol, 0.25 equiv.), (*E*)-enamide **2h** (110 mg, 0.63 mmol, 2.5 equiv.) in a total of 2.5 mL dichloromethane. The reaction was allowed to warm to rt overnight. Column chromatography (*n*-hexane:EtOAc = 9:1→4:1→7:3→1:1) afforded the desired tetrahydropyran **3u** as a colorless solid (109 mg, 77%, isolated d.r. >98: 2; d.r. of the crude mixture 95:5 as determined by ^{1}H NMR analysis of the unpurified product after aqueous workup).

R$_f$ (*n*-hexane:EtOAc = 7:3) 0.33.

m.p. 166-175 °C.

^{1}H NMR (*400 MHz, CDCl$_3$*) δ 7.86 – 7.74 (m, 2H), 7.60 – 7.54 (m, 2H), 7.51 – 7.32 (m, 10H), 7.29 (d, J = 7.3 Hz, 1H), 6.98 (d, J = 8.8 Hz, 1H), 6.42 (d, J = 10.2 Hz, 1H), 5.83 (t, J = 8.8 Hz, 1H), 5.22 (d, J = 6.6 Hz, 1H), 4.92 (ddd, J = 10.2, 5.2, 2.8 Hz, 1H), 2.29 (ddd, J = 10.0, 5.0, 1.5 Hz, 1H), 1.86 (ddt, J = 8.4, 5.4, 2.9 Hz, 1H), 1.62 – 1.45 (m, 2H), 1.28 – 1.12 (m, 2H), 1.10 (t, J = 7.4 Hz, 3H), 0.79 (t, J = 7.3 Hz, 3H).

^{13}C NMR (*101 MHz, CDCl$_3$*) δ 168.0, 167.7, 139.7, 134.2, 134.0, 132.2, 131.8, 128.9, 128.8, 128.7, 128.6, 127.4, 127.3, 127.0, 126.9, 126.4, 78.1, 73.8, 46.8, 46.7, 46.5, 12.5, 12.4.

IR (ATR, ν in cm^{-1}): 3287 (w), 2960 (w), 2932 (w), 2872 (w), 1668 (m), 1623 (s), 1579 (m), 1512 (s), 1486 (s), 1416 (m), 1368 (m), 1329 (m), 1154 (m), 1134 (m), 1106 (w), 1027 (m), 932 (w), 798 (m), 690 (s), 668 (m).

HRMS (EI) m/z calcd for $C_{29}H_{32}N_2O_3$ 456.2413 [M]$^+$, found 456.2408 [M]$^+$.

2.2.22 Tetrahydro-2H-pyran **5a**

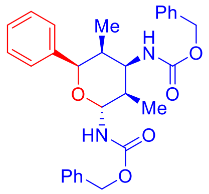

5a

Prepared according to TP1 from benzaldehyde **1a** (25 µL, 0.25 mmol, 1.0 equiv.), BF_3·OEt_2 (1 M in DCM, 0.275 mL, 0.275 mmol, 1.1 equiv.), (*E*)-enecarbamate **4a** (120 mg, 0.63 mmol, 2.5 equiv.) in a total of 2.5 mL dichloromethane. The reaction was allowed to warm to -50 ° over a period of 3h. Column chromatography (*n*-hexane:EtOAc = 9:1→4:1) afforded the desired tetrahydropyran **4a** as a low melting solid (96 mg, 79%, isolated d.r. >98: 2).

R_f (*n*-hexane:EtOAc = 7:3) 0.62.

^{1}H NMR (400 MHz, *$CDCl_3$*) δ 7.62 – 7.03 (m, 15H), 5.95 (s, 1H), 5.37 (s, 1H), 5.10 (d, J = 3.3 Hz, 4H), 5.03 (d, J = 12.1 Hz, 1H), 4.44 – 4.11 (m, 1H), 2.52 – 2.25 (m, 1H), 2.03 – 1.90 (m, 1H), 1.15 (d, J = 7.1 Hz, 3H), 0.58 (d, J = 7.3 Hz, 3H).

^{13}C NMR (*101 MHz, $CDCl_3$*) δ 156.8, 156.5, 139.8, 136.4, 128.7, 128.7, 128.6, 128.4, 128.4, 128.3, 128.2, 128.2, 127.0, 125.9, 81.9, 72.1, 67.2, 67.1, 51.2, 38.1, 36.9, 15.0, 11.2.

IR (ATR, ν in cm^{-1}): 3329 (w), 2970 (w), 2932 (w), 1692 (s), 1587 (w) 1498 (s), 1454 (m), 1399 (m), 1329 (m), 1221 (s), 1026 (s), 972 (s), 915 (m), 778 (w), 738 (m), 695 (s).

HRMS (EI) m/z calcd for $C_{29}H_{32}N_2O_5$ 488,2311 $[M]^+$, found 488.2316 $[M+H]^+$.

2.2.23 Tetrahydro-2H-pyran **5b**

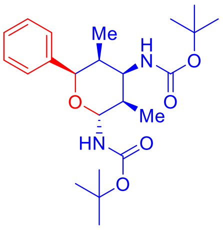

5b

Prepared according to TP1 from benzaldehyde **1a** (25 µL, 0.25 mmol, 1.0 equiv.), $BF_3{\cdot}OEt_2$ (1 M in DCM, 0.275 mL, 0.275 mmol, 1.1 equiv.), (*E*)-enecarbamate **4b** (120 mg, 0.63 mmol, 2.5 equiv.) in a total of 2.5 mL dichloromethane. The reaction was allowed to warm to -50 ° over a period of 3h. Column chromatography (*n*-hexane:EtOAc = 9:1→4:1) afforded the desired tetrahydropyran **5b** as a low melting solid (23 mg, 30%, isolated d.r. >98: 2).

$\mathbf{R_f}$ (*n*-hexane:EtOAc = 7:3) 0.62.

^{1}H NMR (*400 MHz, DMSO-d_6***)** δ 7.42 – 7.13 (m, 6H), 6.95 (d, *J* = 8.4 Hz, 1H), 5.02 (d, *J* = 10.2 Hz, 1H), 4.69 (d, *J* = 2.7 Hz, 1H), 4.10 – 3.89 (m, 1H), 2.10 – 1.94 (m, 1H), 1.93 – 1.82 (m, 1H), 1.43 – 1.38 (m, 18H), 0.95 (d, *J* = 7.2 Hz, 3H), 0.49 (d, *J* = 7.3 Hz, 3H).

^{13}C NMR (*101 MHz, DMSO-d_6*) δ 155.2, 154.9 , 140.6, 127.9, 126.6, 125.4, 82.7, 79.2, 78.6 , 77.9, 53.5, 36.7, 36.1, 28.3, 28.2, 9.7, 9.0.

IR (ATR, ν in cm^{-1}): 3362 (w), 2980 (m), 2933 (w), 1694 (s), 1510 (m), 1453 (m), 1393 (m), 1366 (s), 1244 (s), 1158 (s), 110 (m), 1055 (m), 1004 (m), 977 (m), 905 (m), 858 (w), 807 (w), 784 (m), 720 (m), 699 (m).

HRMS (EI) m/z calcd for $C_{23}H_{36}N_2O_5$ 420.2624 $[M]^+$, found 420.2627 $[M]^+$.

2.2.24 Tetrahydro-2H-pyran **6a**

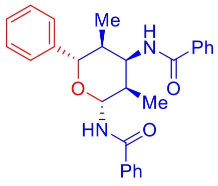

6a

Prepared according to TP1 from benzaldehyde **1a** (51 µL, 0.50 mmol, 1.0 equiv.), $BF_3{\cdot}OEt_2$ (70 µmol, 0.55 mmol, 1.1 equiv.), (*Z*)-enamide **2a** (201 mg, 1.25 mmol, 2.5 equiv.) in a total of 5 mL dichloromethane. The reaction was allowed to warm to rt overnight. Column chromatography (*n*-hexane:EtOAc = 9:1→4:1→7:3→1:1) afforded the desired Tetrahydropyran **6a** as a colorless solid (148 mg, 69 %). **6a** was isolated with minor amounts of **3a** (45 mg, 21 %). The combined yield of **6a** and **3a** was 192 mg, 90 % (isolated d.r. 77: 23 (**6a**: **3a**) as calculated from the isolated product; d.r. of the crude mixture 85: 15 (**6a**: **3a**); as determined by ^{1}H NMR analysis of the unpurified product after aqueous workup).

R_f (*n*-hexane:EtOAc = 7:3) 0.19.

m.p. 235-245 °C.

^{1}H NMR (*300 MHz, CDCl₃*) δ 8.00 – 7.81 (m, 2H), 7.77 – 7.65 (m, 2H), 7.60 – 7.36 (m, 8H), 7.30 (s, 4H), 6.66 (d, J = 10.6 Hz, 1H), 6.53 (d, J = 9.5 Hz, 1H), 5.63 (t, J = 10.1 Hz, 1H), 4.84 (dt, J = 10.6, 3.9 Hz, 1H), 4.49 (d, J = 10.9 Hz, 1H), 2.22 (ddt, J = 10.5, 6.9, 3.5 Hz, 2H), 1.09 (d, J = 6.9 Hz, 3H), 0.71 (d, J = 6.9 Hz, 3H).

^{13}C NMR (*75 MHz, $CDCl_3$*) δ 168.9, 167.4, 139.5, 134.8, 133.9, 132.2, 131.8, 128.9, 128.8, 128.6, 128.5, 127.5, 127.2, 127.2, 81.2, 79.6, 52.8, 40.9, 40.8, 13.7, 13.2.

IR (ATR, ν in cm^{-1}): 3330 (m), 2970 (m), 2934 (w), 1641 (s), 1604 (w), 1581 (m), 1525 (s), 1489 (s), 1459 (m), 1380 (w), 1351 (m), 1280 (m), 1260 (m), 1184 (w), 1156 (w) 1068 (s) 1027 (s) 962 (m), 945 (w), 914 (w), 842 (w), 821 (w) 758 (m), 681 (s), 690 (s), 669 (s).

HRMS (EI) m/z calcd for $C_{27}H_{28}N_2O_3$ 475.0657 $[M]^+$, found 428.2104 $[M]^+$.

2.2.25 Tetrahydro-2H-pyran **6c**

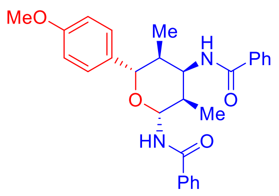

6c

Prepared according to TP1 from 4-methoxybenzaldehyde **1c** (51 µL, 0.50 mmol, 1.0 equiv.), $BF_3 \cdot OEt_2$ (70 µmol, 0.55 mmol, 1.1 equiv.), (*Z*)-enamide **2a** (201 mg, 1.25 mmol, 2.5 equiv.) in a total of 5 mL dichloromethane. The reaction was allowed to warm to rt overnight. Column chromatography (*n*-hexane:EtOAc = 9:1→4:1→7:3→1:1) afforded the desired Tetrahydropyran **6c** as a colorless solid (117 mg, 51%, isolated d.r. 67: 33; **6c** was isolated with minor amounts of **3c** (57 mg, 25 %). The combined yield of **6c** and **3c** was 175 mg, 76 % (isolated d.r. 67: 33 (**6c**: **3c**) as calculated from the isolated product; d.r. of the crude mixture 75: 25 (**6c**: **3c**); as determined by ^{1}H NMR analysis of the unpurified product after aqueous workup).

R_f (*n*-hexane:EtOAc = 6:4) 0.08.

m.p. 238-245 °C.

^{1}H NMR (*400 MHz, CDCl$_3$*) δ 7.91 – 7.76 (m, 2H), 7.76 – 7.65 (m, 2H), 7.55 – 7.42 (m, 2H), 7.38 (td, J = 7.5, 2.2 Hz, 4H), 7.24 – 7.13 (m, 2H), 6.91 (d, J = 10.5 Hz, 1H), 6.81 (d, J = 8.7 Hz, 2H), 6.56 (d, J = 9.5 Hz, 1H), 5.69 (t, J = 10.0 Hz, 1H), 4.85 (dt, J = 10.5, 4.0 Hz, 1H), 4.52 (d, J = 10.9 Hz, 1H), 3.76 (s, 3H), 2.31 – 2.17 (m, 2H), 1.08 (d, J = 6.8 Hz, 3H), 0.71 (d, J = 6.8 Hz, 3H).

^{13}C NMR (101 MHz, *CDCl$_3$*) δ 169.0, 167.4, 159.6, 134.9, 133.8, 132.1, 131.8, 131.7, 128.7, 128.6, 127.3, 127.2, 127.1, 114.0, 80.6, 79.6, 55.4, 52.8, 40.9, 40.5, 13.7, 13.2.

MS (ESI) m/z calcd for $C_{28}H_{30}N_2O_4$ 476.3 [M+H]$^+$, found 476.8 [M+H]$^+$.

IR (ATR, ν in cm^{-1}): 3283 (m), 2964 (m), 2933 (m), 1636 (s), 1580 (m), 1532 (s), 1488 (s), 1456 (w), 1381 (w), 1351 (w), 1304 (m), 1247 (s), 1174 (m), 1159 (m), 1026 (s), 967 (w), 926 (w), 848 (m), 785 (m), 711 (m).

HRMS (EI) m/z calcd for $C_{28}H_{30}N_2O_4$ 458.2206 [M+H]$^+$, found 458.2197 [M]$^+$.

2.2.26 Tetrahydro-2H-pyran **6l**

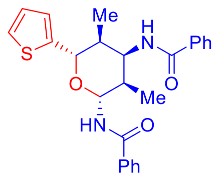

6l

Prepared according to TP1 from Aldehyde **1l** (47 µL, 0.50 mmol, 1.0 equiv.), $BF_3{\cdot}OEt_2$ (70 µmol, 0.55 mmol, 1.1 equiv.), (*Z)*-enamide **2a** (201 mg, 1.25 mmol, 2.5 equiv.) in a total of 5 mL dichloromethane. The reaction was allowed to warm to rt overnight: Column chromatography (*n*-hexane:EtOAc = 9:1→4:1→7:3→1:1) afforded the desired tetrahydropyran **6l** as a colorless solid (131 mg, 60 %). **6l** was isolated with minor amounts of **3l** (27 mg, 13 %). The combined yield of **6l** and **3l** was 158 mg, 73 % (isolated d.r. 85: 15 (**6l**: **3l**) as calculated from the isolated product; d.r. of the crude mixture 80: 20 (**6l**: **3l**); as determined by ^{1}H NMR analysis of the unpurified product after aqueous workup).

R_f (*n*-hexane:EtOAc = 1:1) 0.16.

m.p. *decomposition before melting*.

^{1}H NMR (*400 MHz, DMSO-d_6)* δ 8.95 (d, J = 9.0 Hz, 1H), 8.32 (d, J = 10.3 Hz, 1H), 7.89 (ddd, J = 8.6, 4.4, 1.4 Hz, 4H), 7.69 – 7.28 (m, 8H), 7.11 – 6.82 (m, 2H), 5.66 (dd, J = 10.6, 9.0 Hz, 1H), 5.20 (d, J = 10.8 Hz, 1H), 4.69 (dt, J = 9.4, 4.2 Hz, 1H), 2.39 – 2.26 (m, 1H), 2.11 – 1.95 (m, 1H), 0.84 (d, J = 6.8 Hz, 3H), 0.61 (d, J = 6.8 Hz, 3H).

^{13}C NMR (*101 MHz, DMSO-d_6)* δ 168.3, 166.6, 144.0, 135.1, 134.0, 131.6, 131.0, 128.3, 128.1, 127.8, 127.6, 126.3, 125.5, 125.4, 78.9, 74.2, 51.8, 41.3, 38.0, 13.5, 13.0.

IR (ATR, ν in cm^{-1}): 3061 (w), 2971 (w), 1638 (s), 1603 (w), 1580 (w), 1529 (s), 1489 (s), 1380 (w), 1350 (w), 1293 (m), 1185 (w), 1160 (w), 1098 (w), 1066 (s), 1027 (m), 1011 (m), 848 (m), 814 (m), 807 (m), 693 (s), 670 (m).

HRMS (EI) m/z calcd for $C_{25}H_{26}N_2O_3S$ 434.1664 $[M]^+$, found 434.1668 $[M]^+$.

3 NMR Data

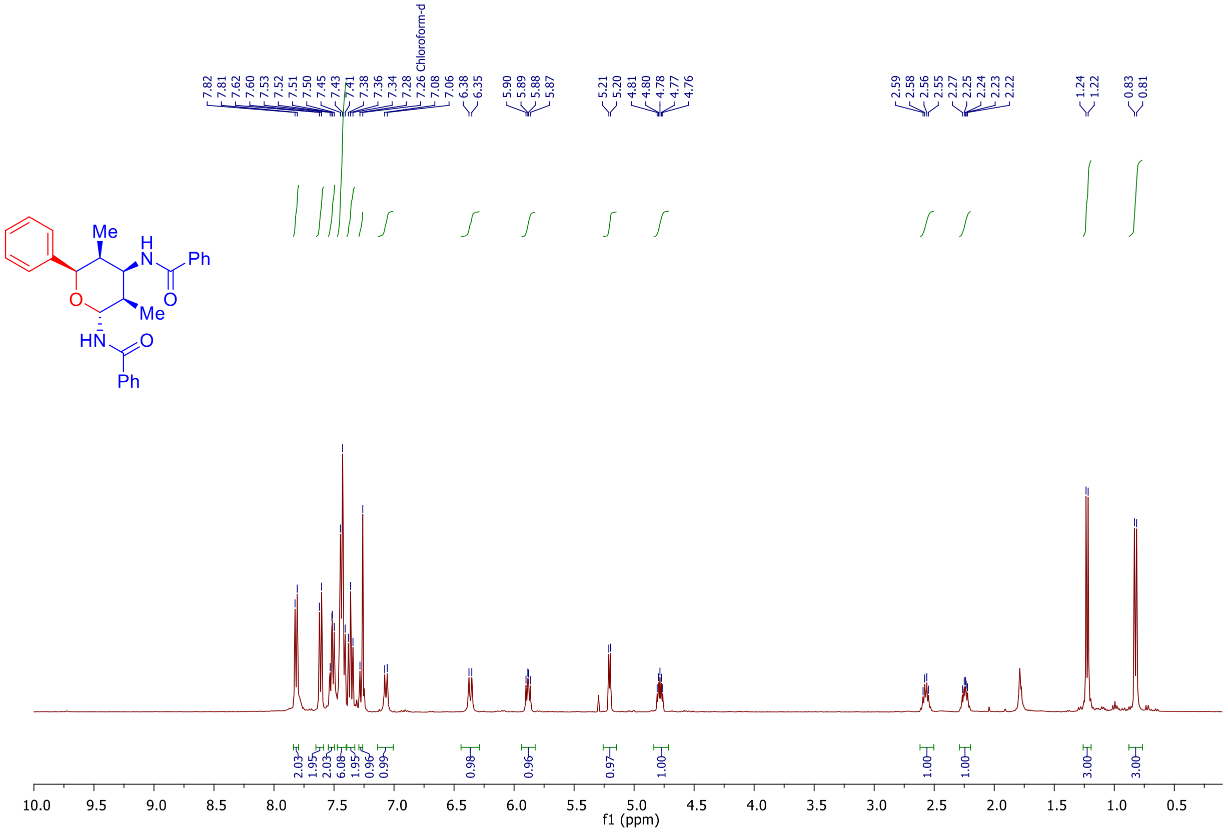

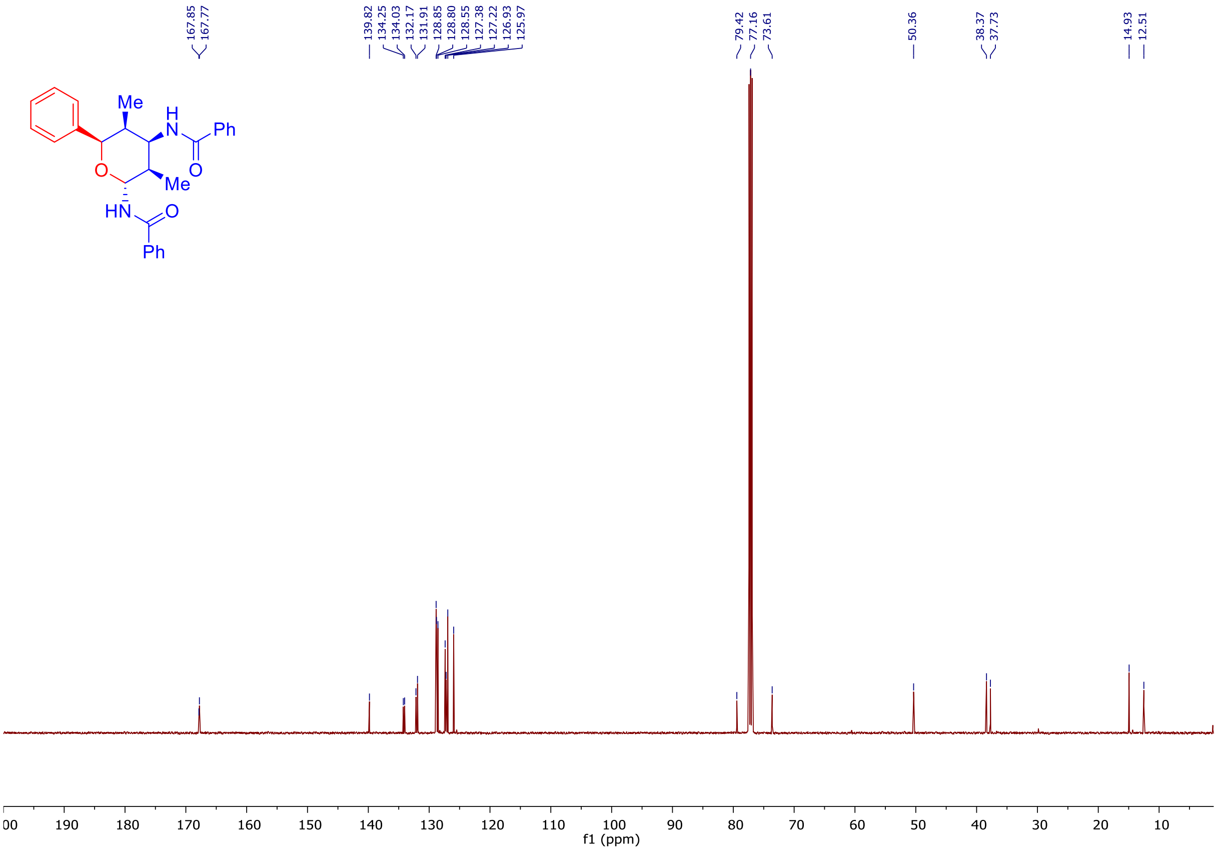

*Figure 1 ^{1}H (400 MHz) and ^{13}C (126 MHz) NMR spectra of **3a** in $CDCl_3$.*

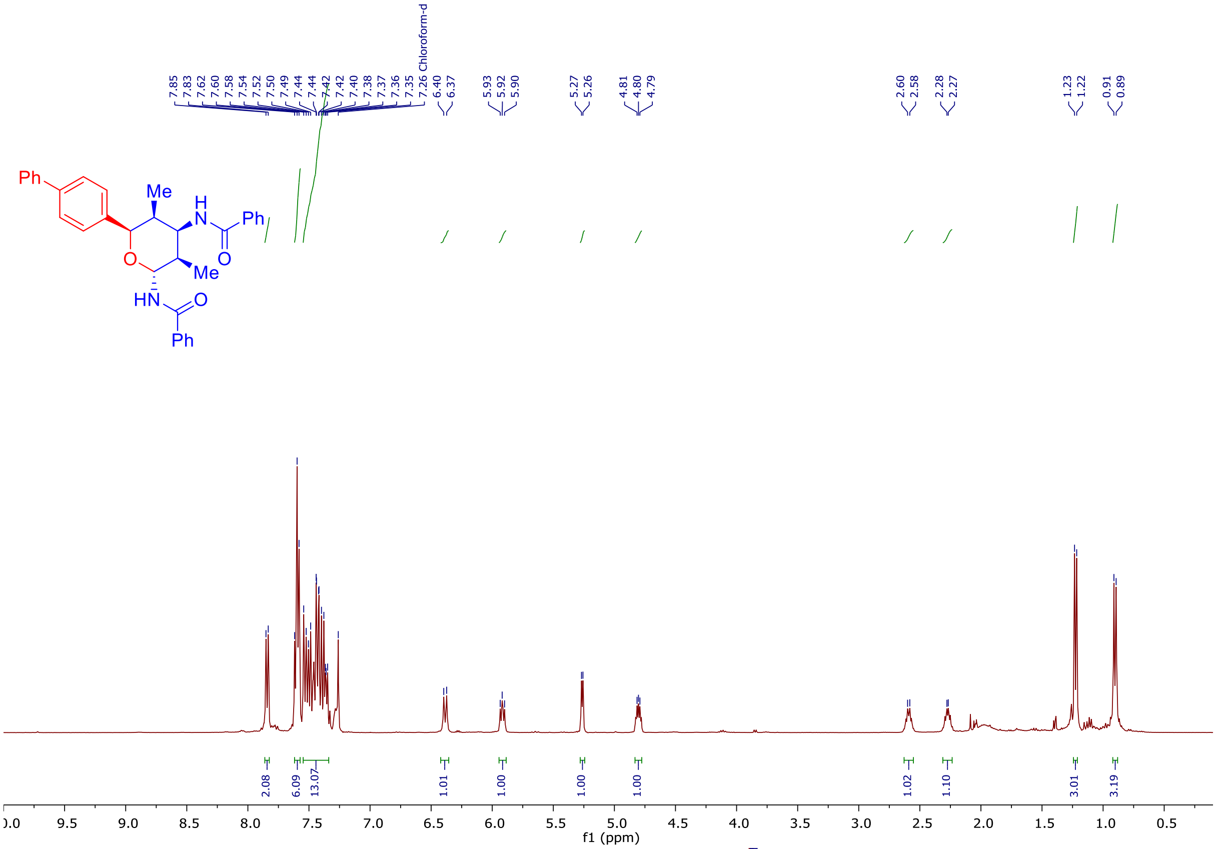

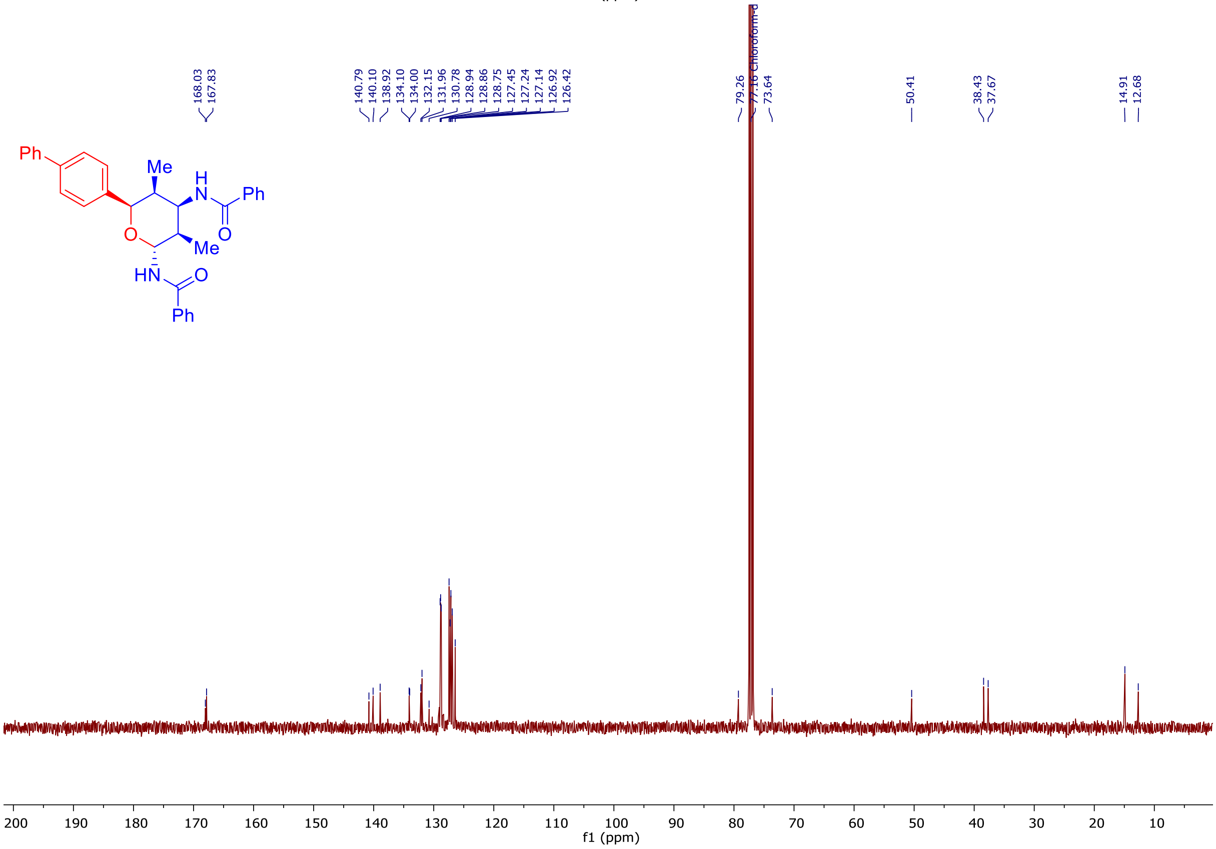

*Figure 2 ^{1}H (400 MHz) and ^{13}C (126 MHz) NMR spectra of **3b** in $CDCl_3$.*

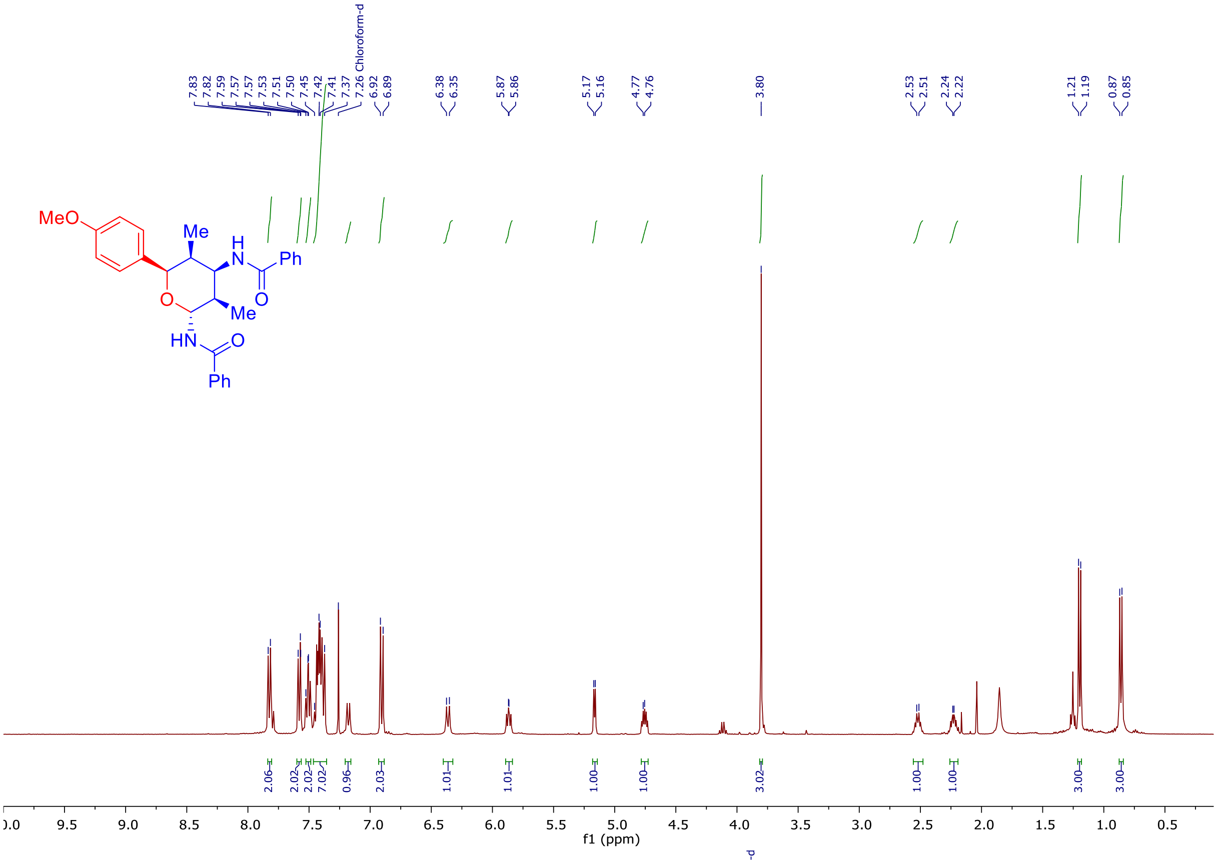

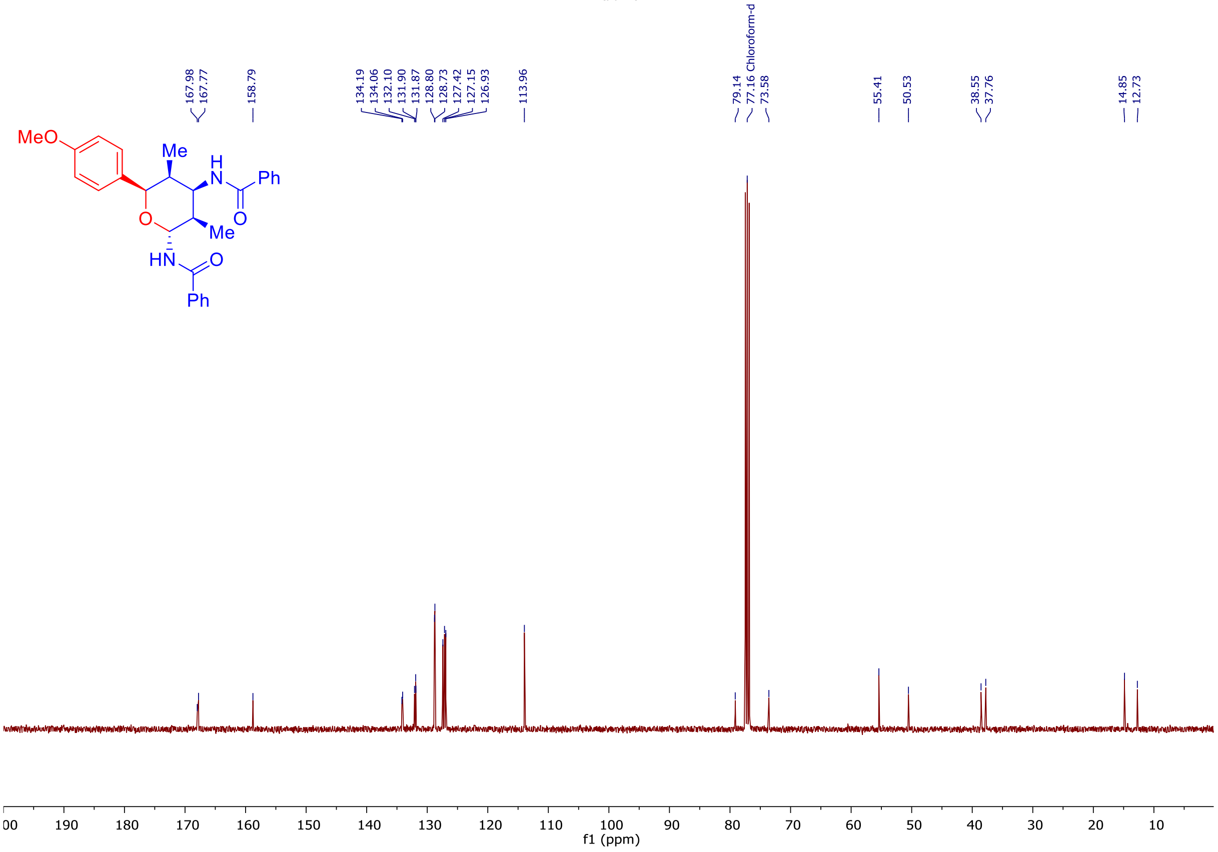

Figure 3: ^{1}H (500 MHz) and ^{13}C (126 MHz) NMR spectra of **3c** in $CDCl_3$.

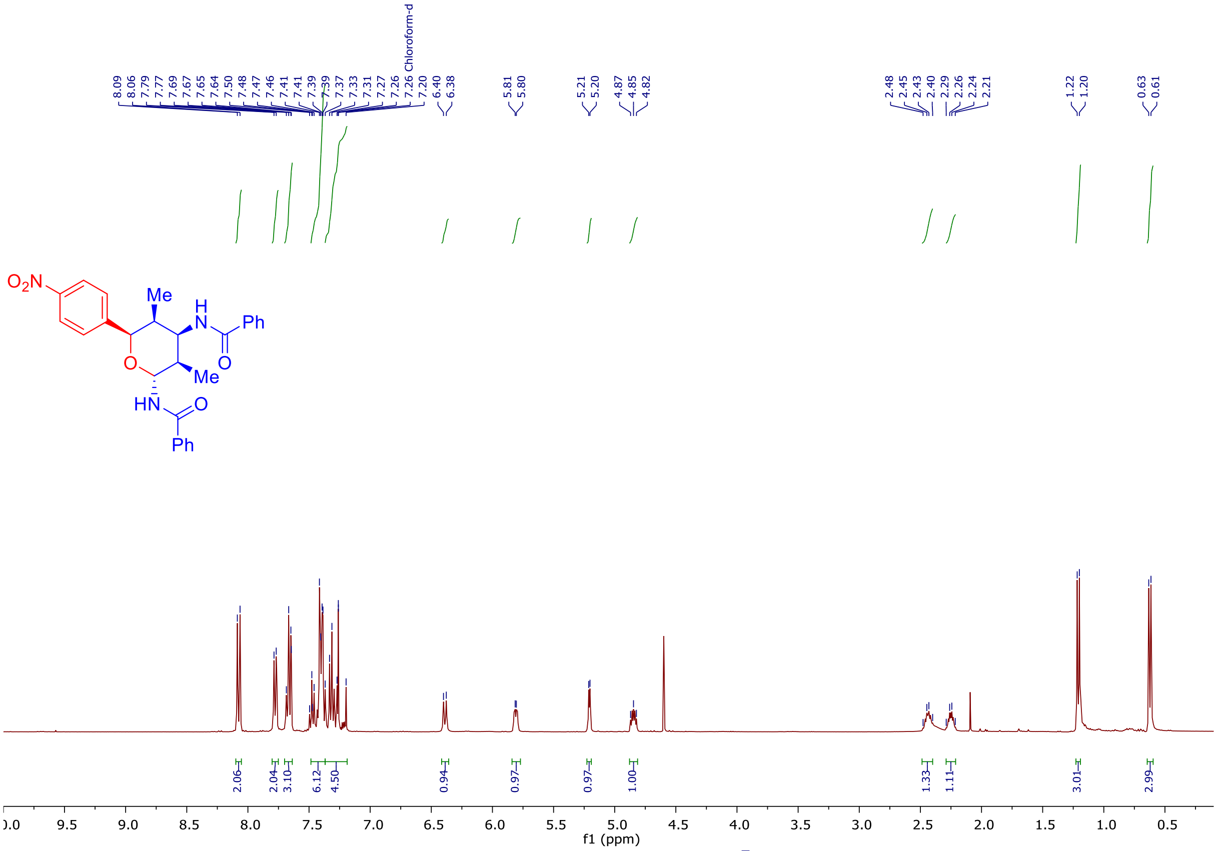

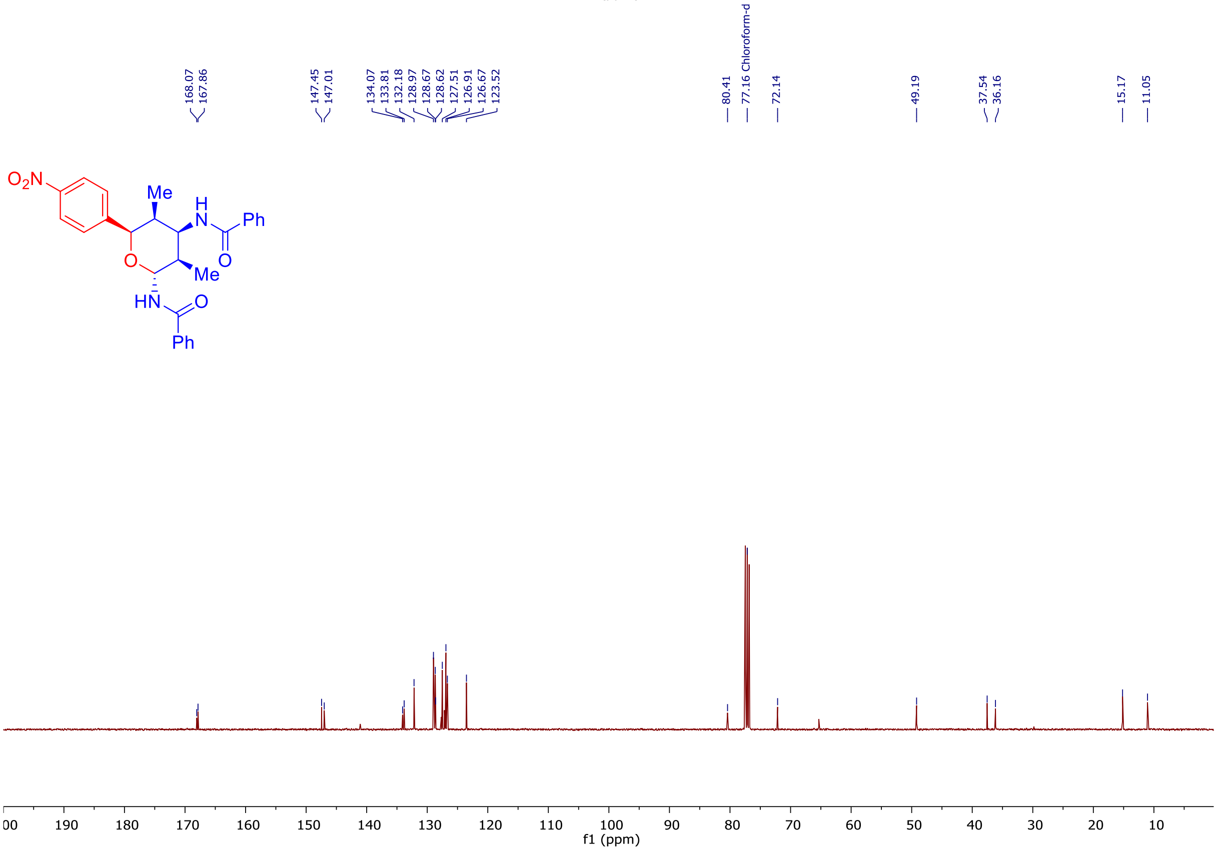

*Figure 4 ^{1}H (500 MHz) and ^{13}C (126 MHz) NMR spectra of **3d** in $CDCl_3$.*

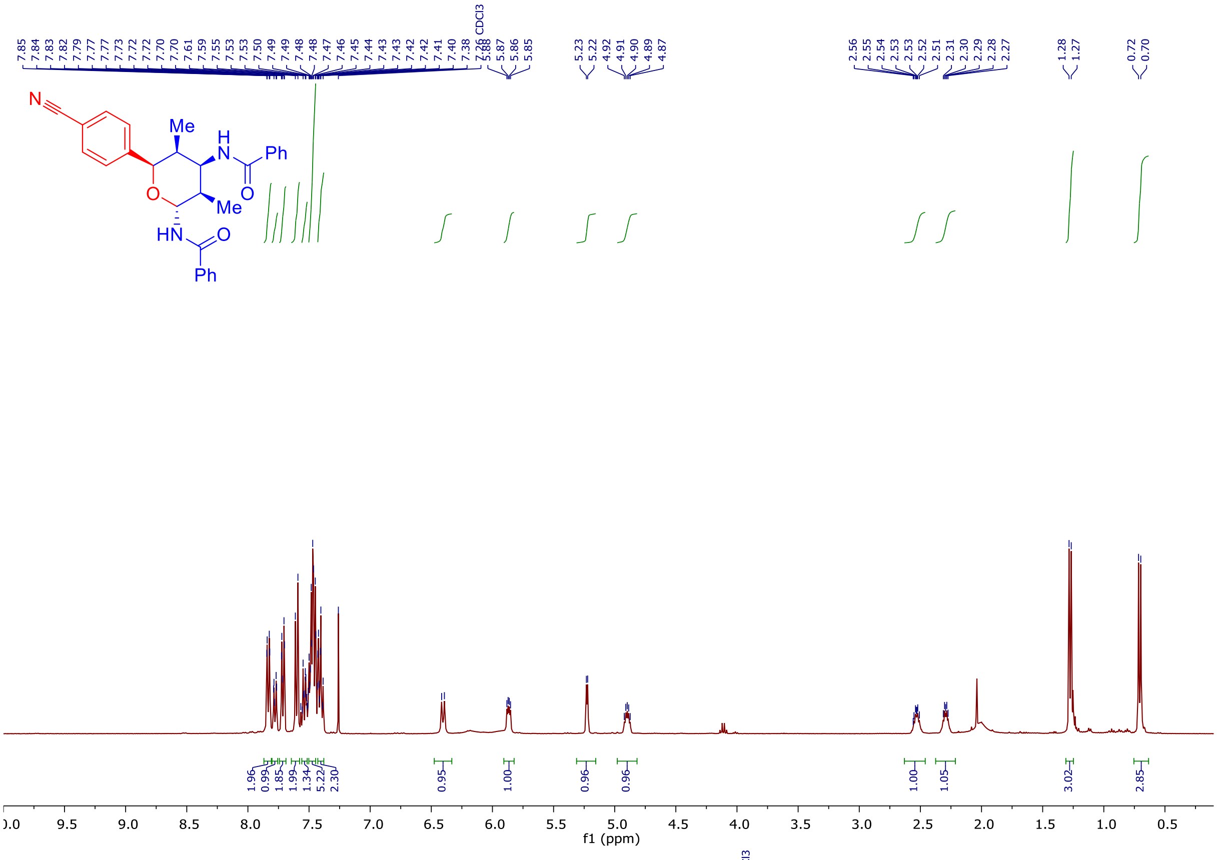

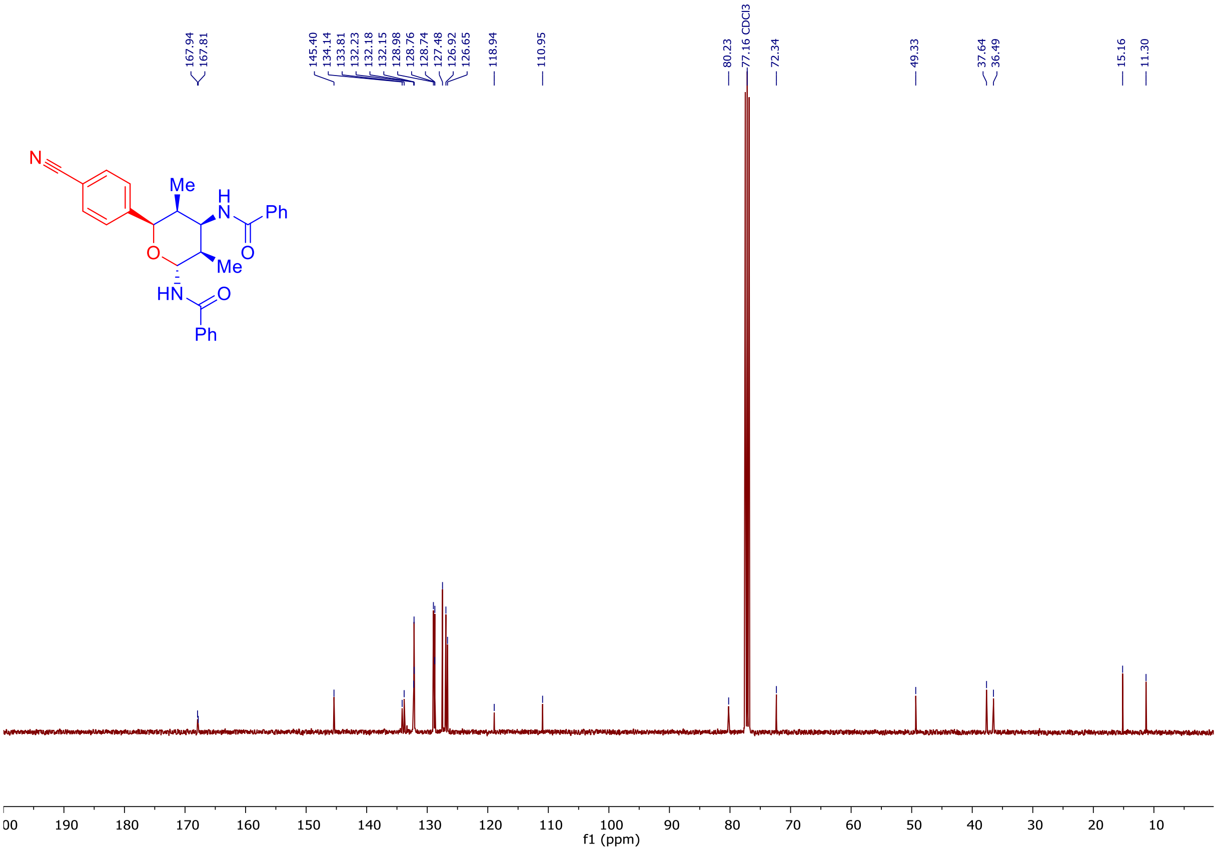

*Figure 5 ^{1}H (400 MHz) and ^{13}C (101 MHz) NMR spectra of **3e** in $CDCl_3$.*

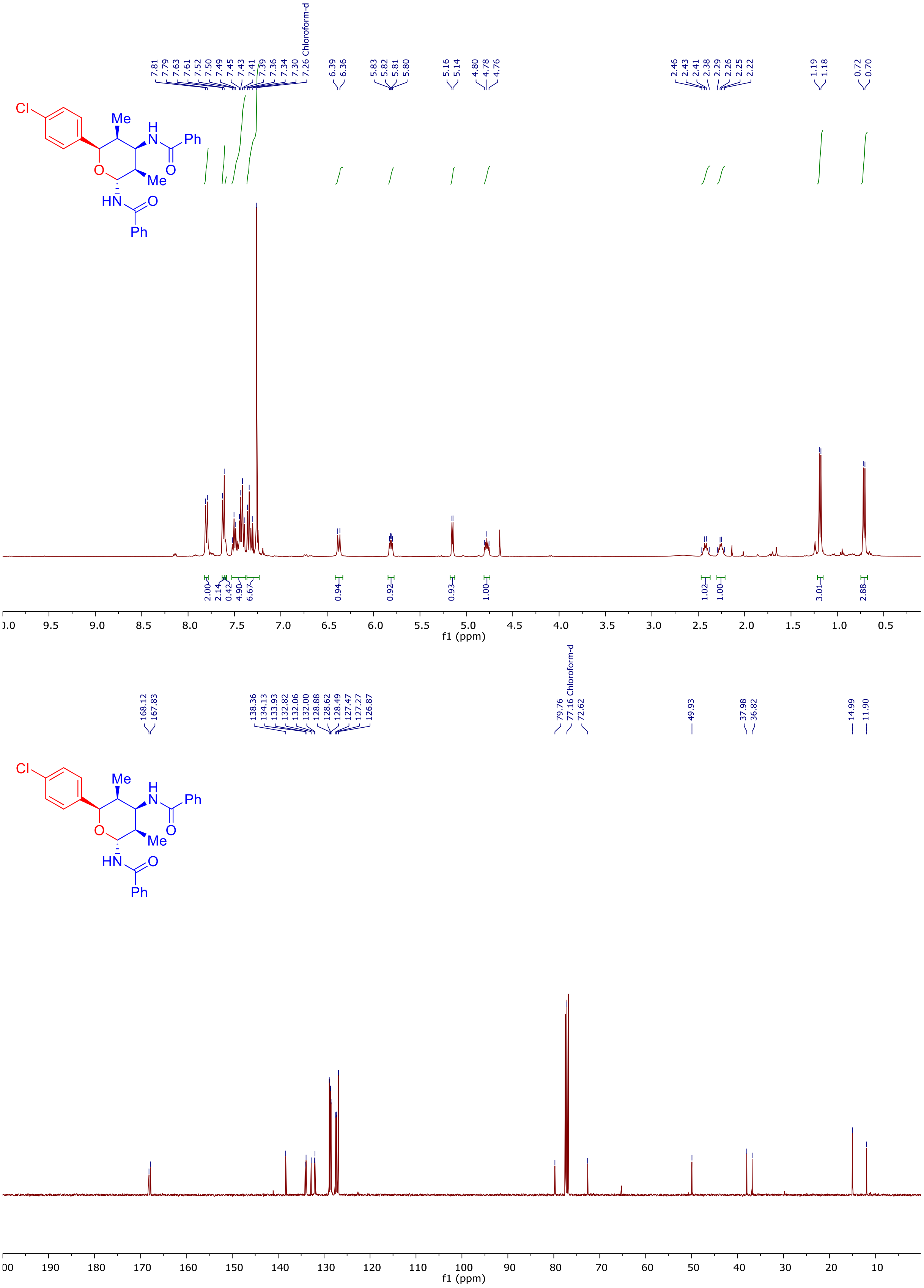

Figure 6 ^{1}*H (400 MHz) and* ^{13}*C (126 MHz) NMR spectra of* ***3f*** *in* $CDCl_3$.

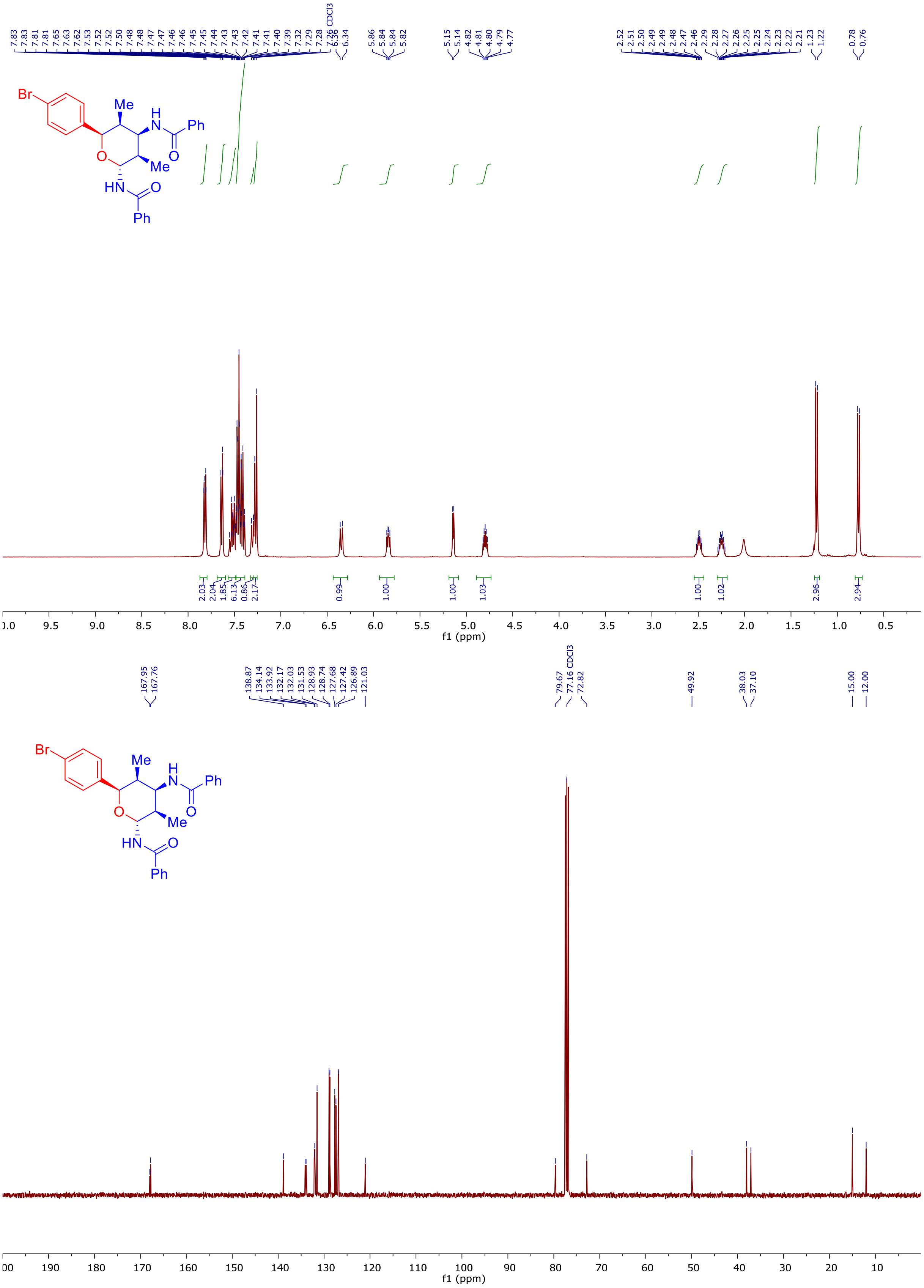

Figure 7 ^{1}H (400 MHz) and ^{13}C (101 MHz) NMR spectra of **3g** in $CDCl_3$.

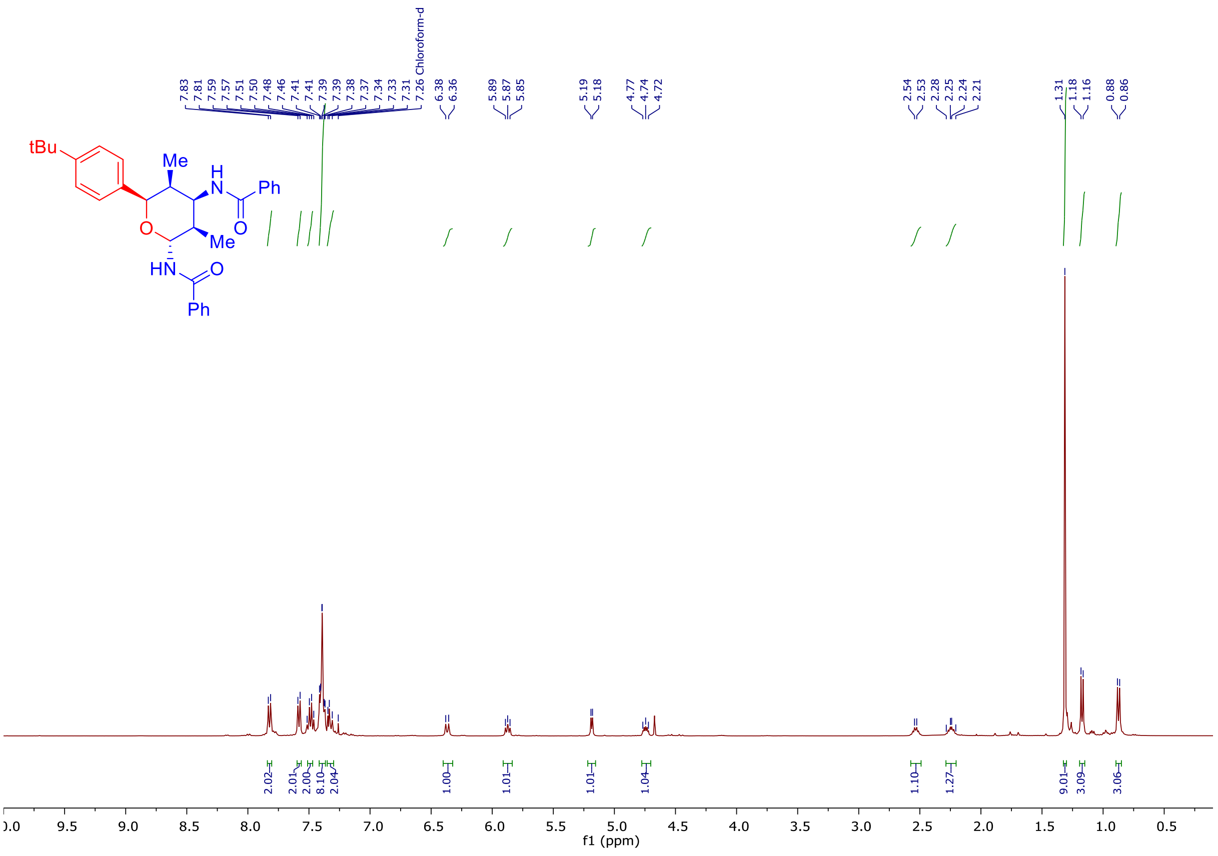

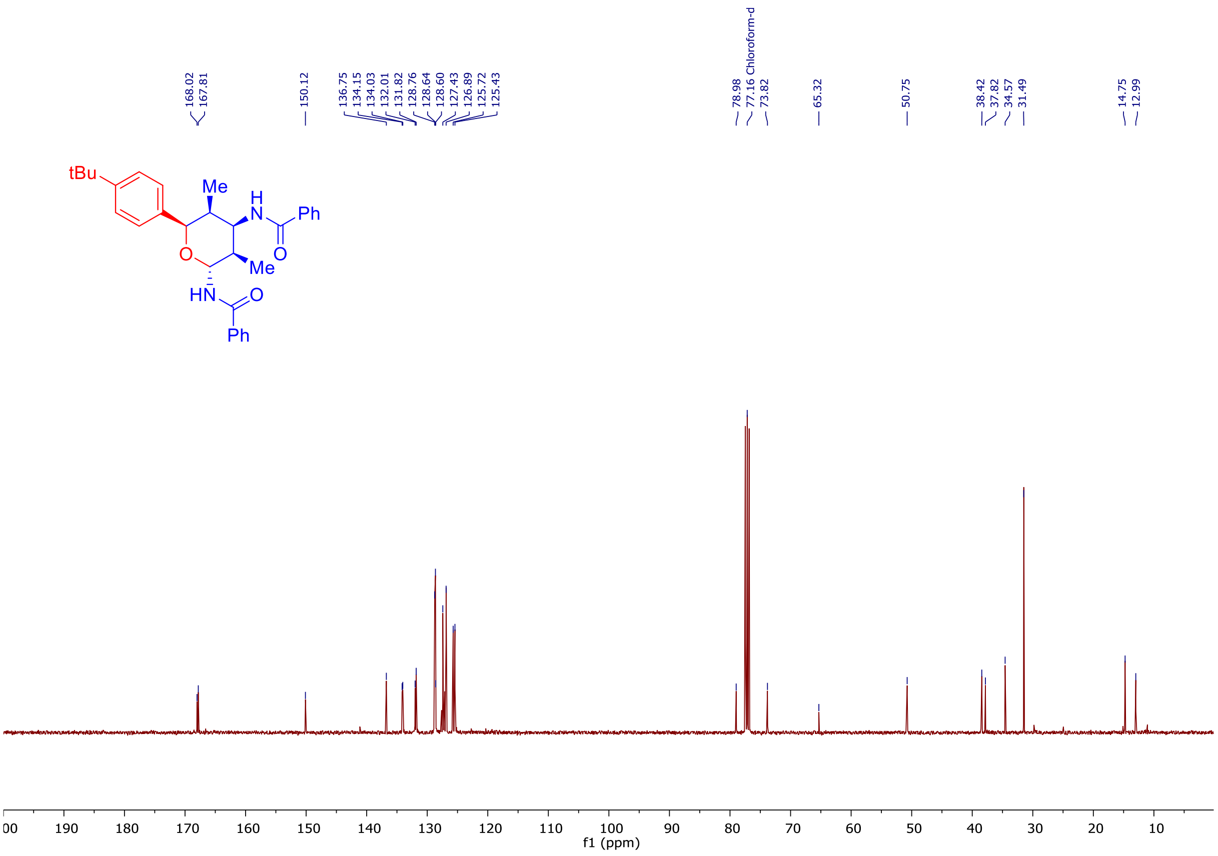

Figure 8 ^{1}H *(400 MHz) and* ^{13}C *(126 MHz) NMR spectra of* ***3h*** *in* $CDCl_3$.

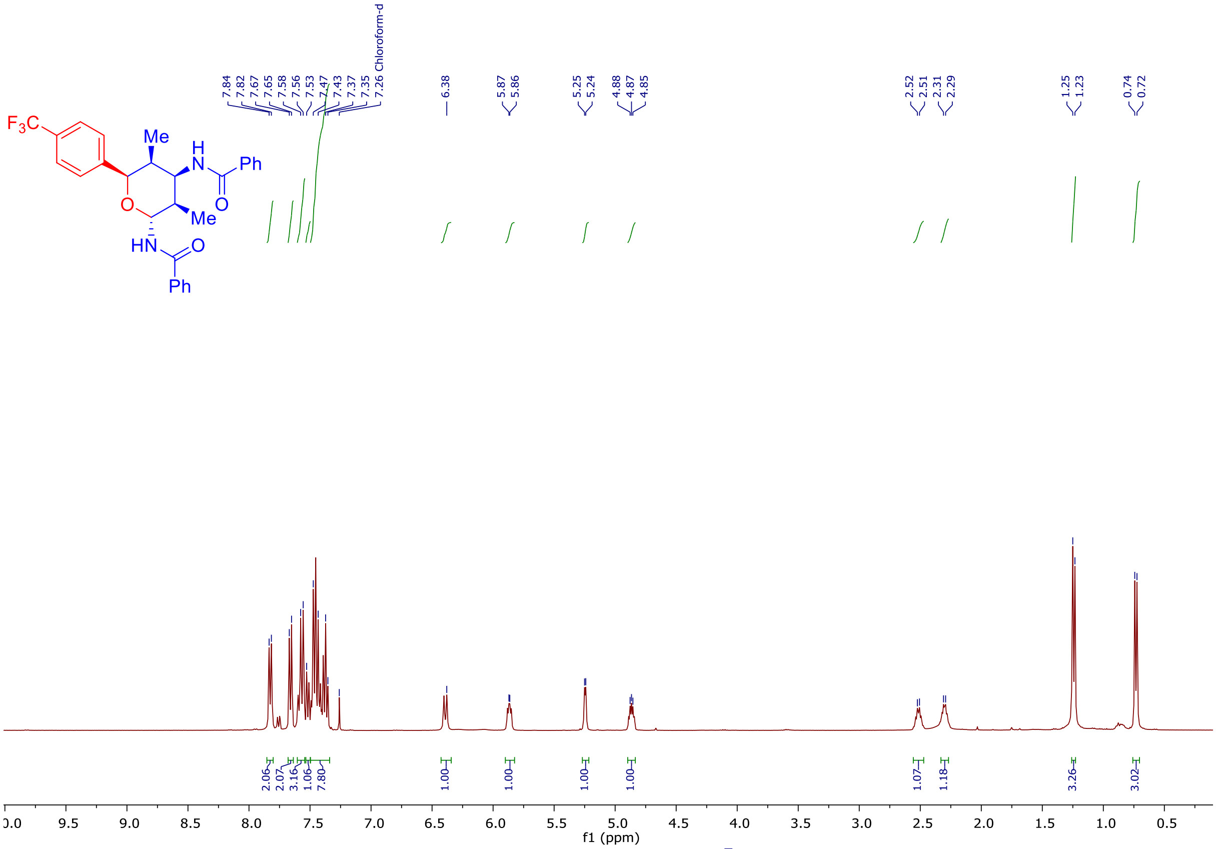

7.84 7.82 7.67 7.65 7.58 7.56 7.53 7.47 7.43 7.37 7.35 7.26 Chloroform-d
6.38
5.87 5.86
5.25 5.24
4.88 4.87 4.85
2.52 2.51 2.31 2.29
1.25 1.23
0.74 0.72
F_3C
Me
H
N
Ph
O
O
Me
HN
O
Ph
2.06 2.07 3.16 1.06 7.80
1.00
1.00
1.00
1.00
1.07
1.18
3.26
3.02
f1 (ppm)

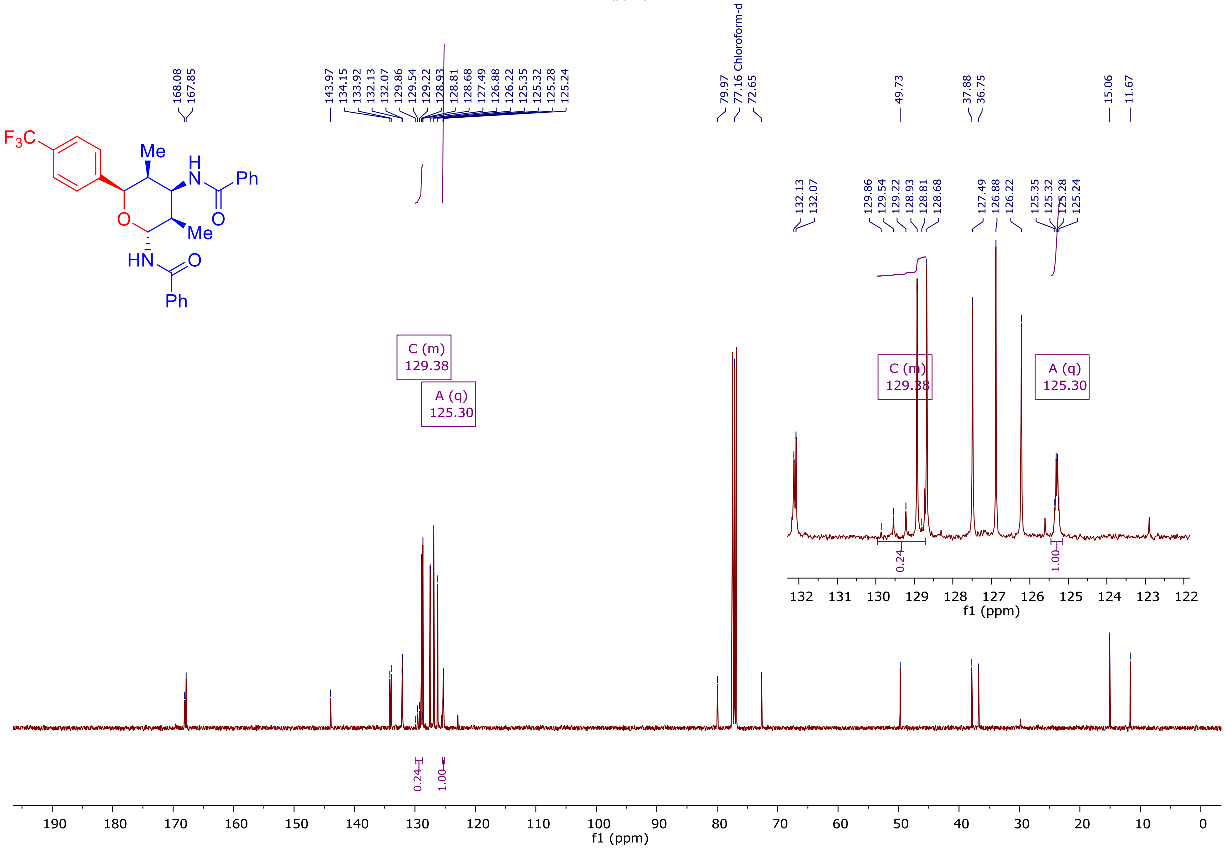

168.08 167.85
143.97 134.15 133.92 132.13 132.07 129.86 129.54 129.22 128.93 128.81 128.68 127.49 126.88 126.22 125.35 125.32 125.28 125.24
79.97 77.16 Chloroform-d 72.65
49.73
37.88 36.75
15.06 11.67
F_3C
Me
H
N
Ph
O
O
Me
HN
O
Ph
C (m)
129.38
A (q)
125.30
0.24
1.00
132.13 132.07
129.86 129.54 129.22 128.93 128.81 128.68
127.49 126.88 126.22
125.35 125.32 125.28 125.24
C (m)
129.38
A (q)
125.30
0.24
1.00
f1 (ppm)
f1 (ppm)

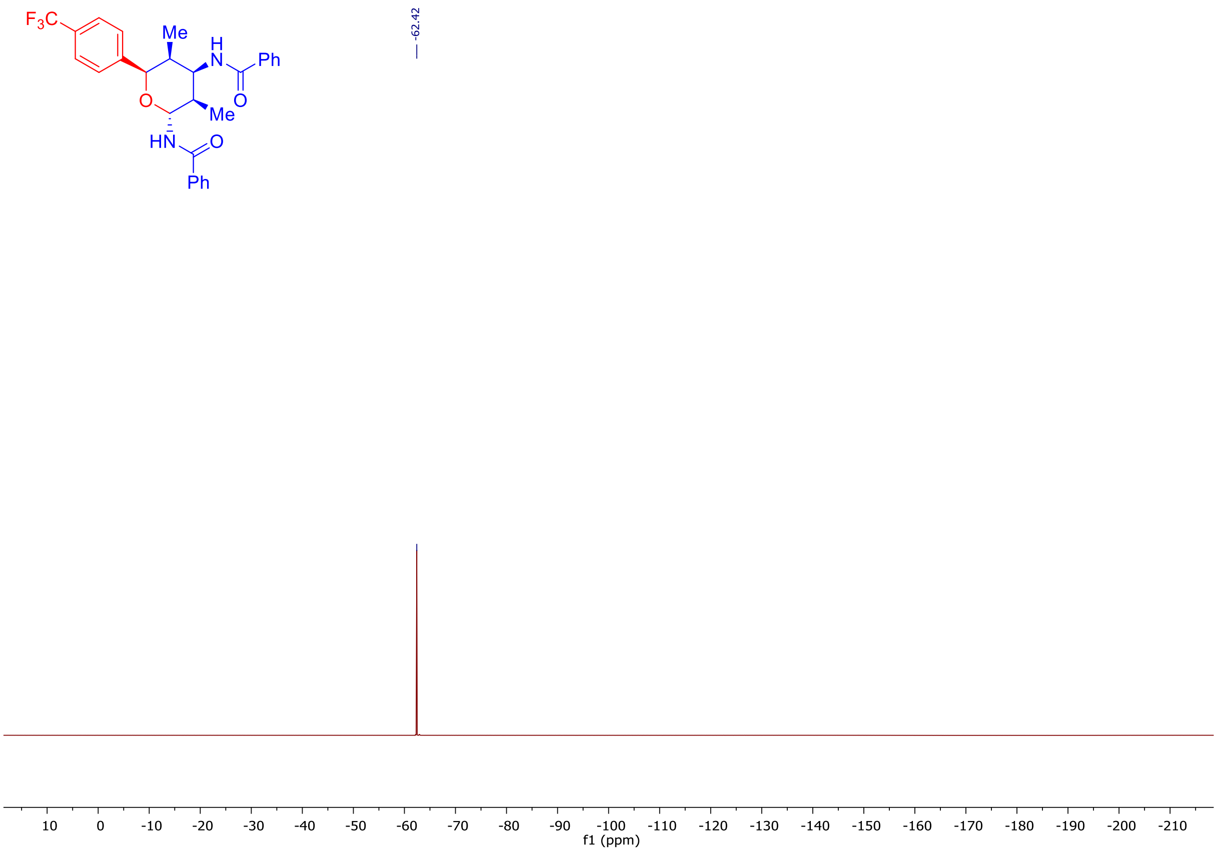

Figure 9: *^{1}H (400 MHz), ^{13}C (126 MHz) and ^{19}F (376 MHz) NMR spectra of* ***3i*** *in $CDCl_3$.*

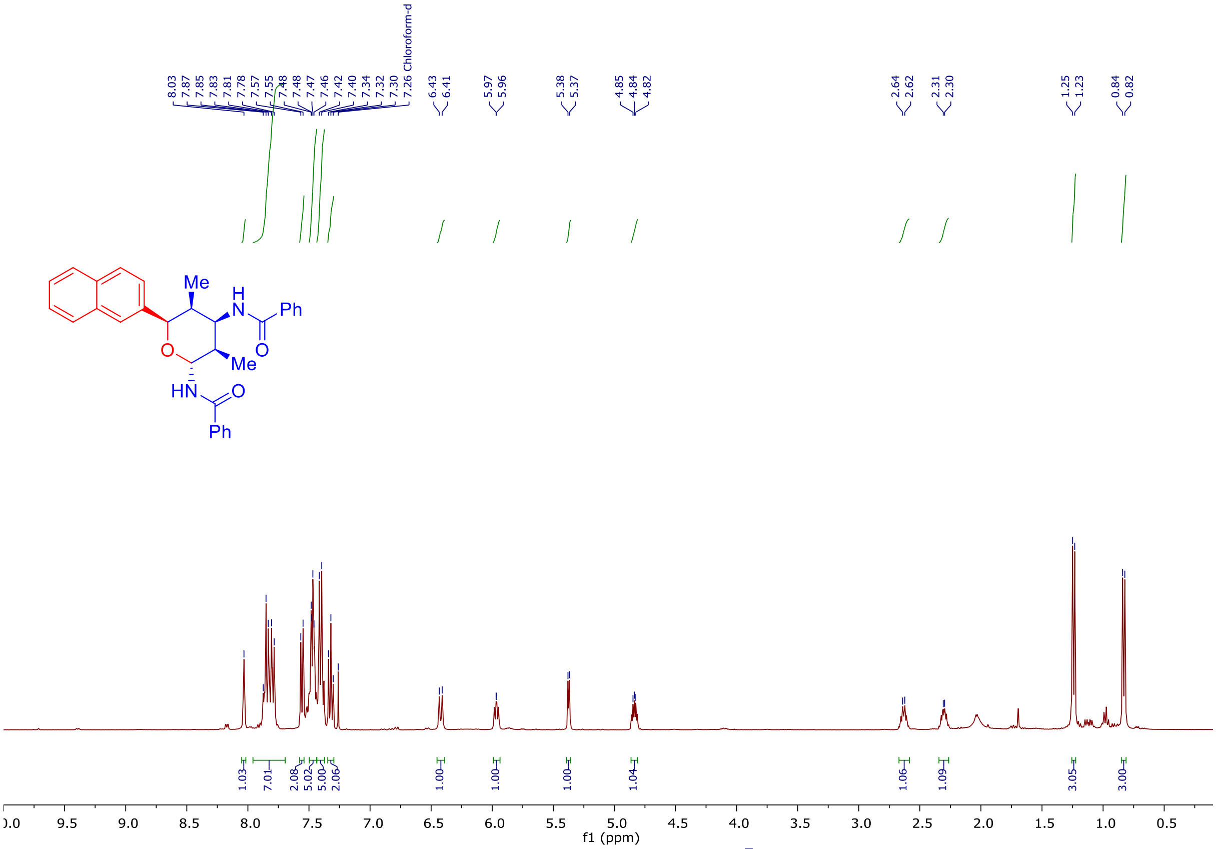

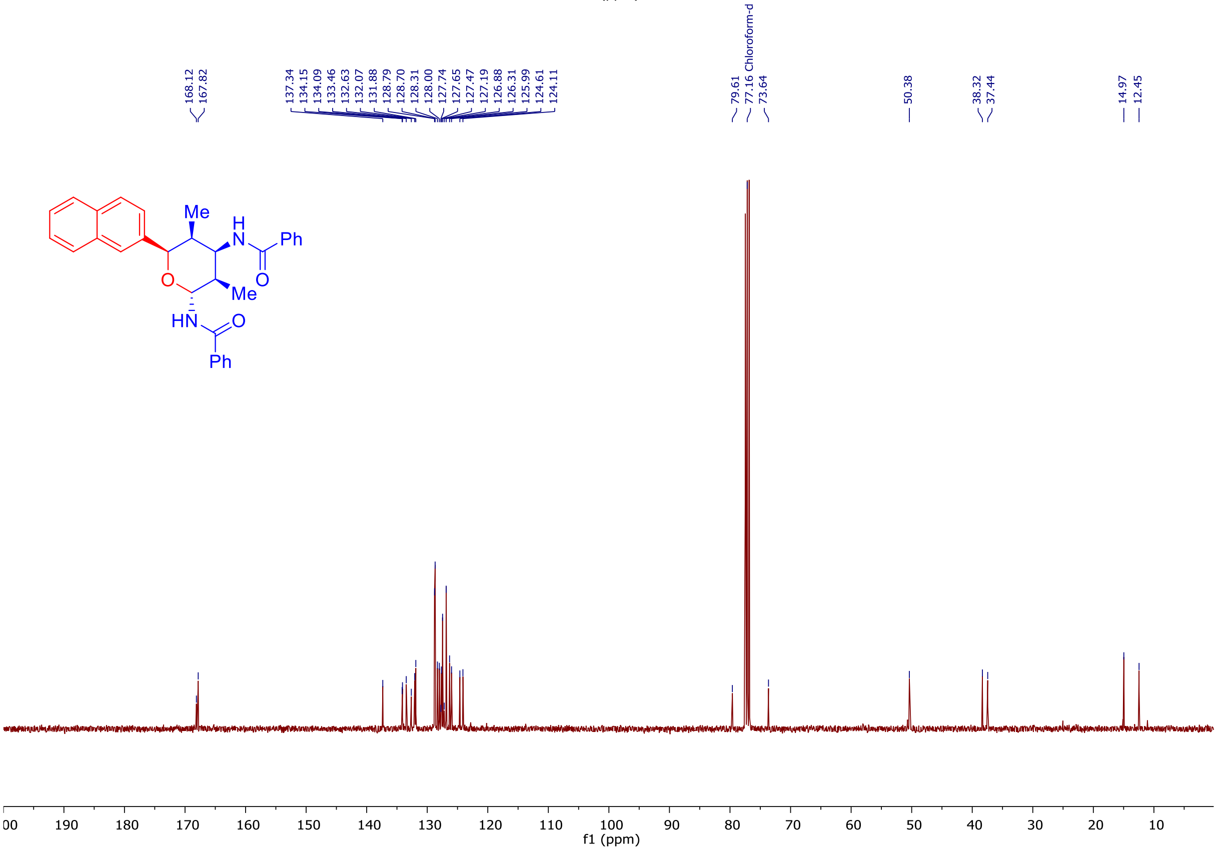

*Figure 10 ^{1}H (400 MHz) and ^{13}C (126 MHz) NMR spectra of **3j** in $CDCl_3$.*

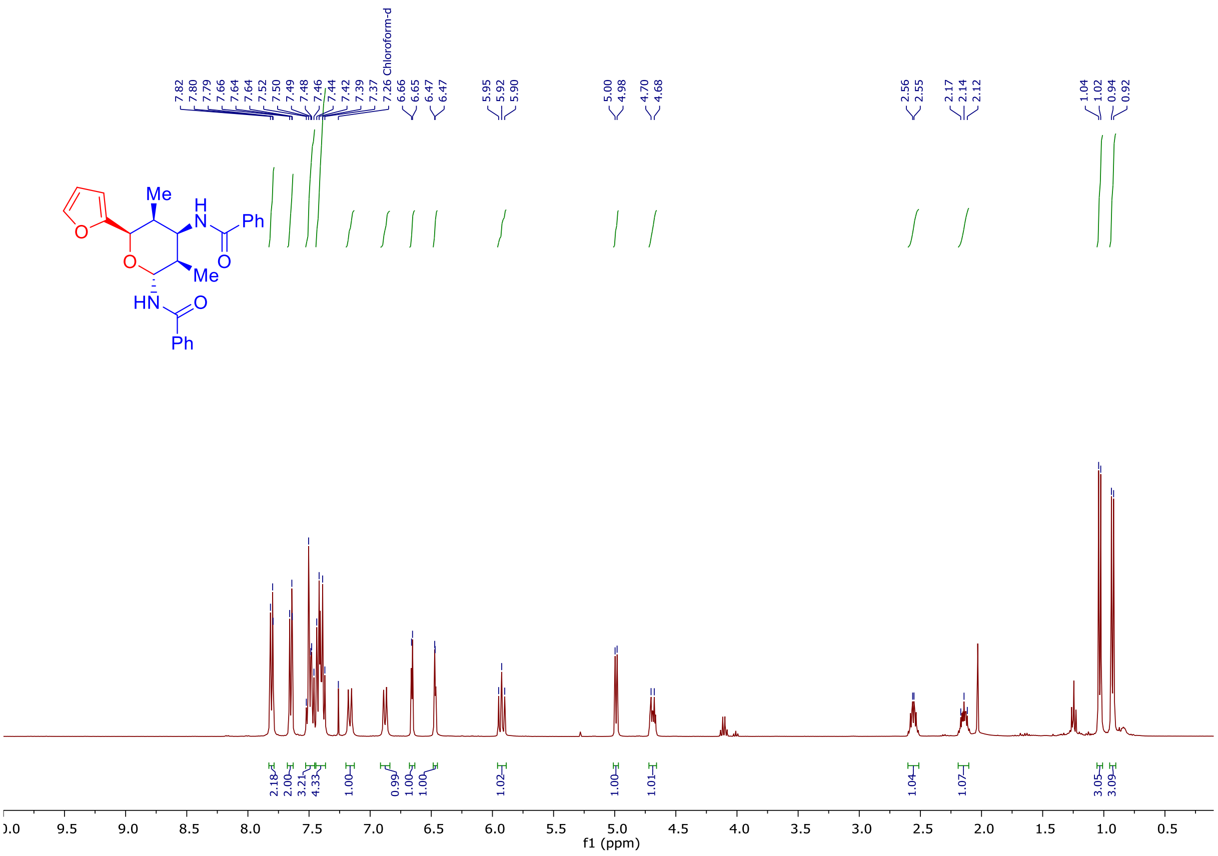

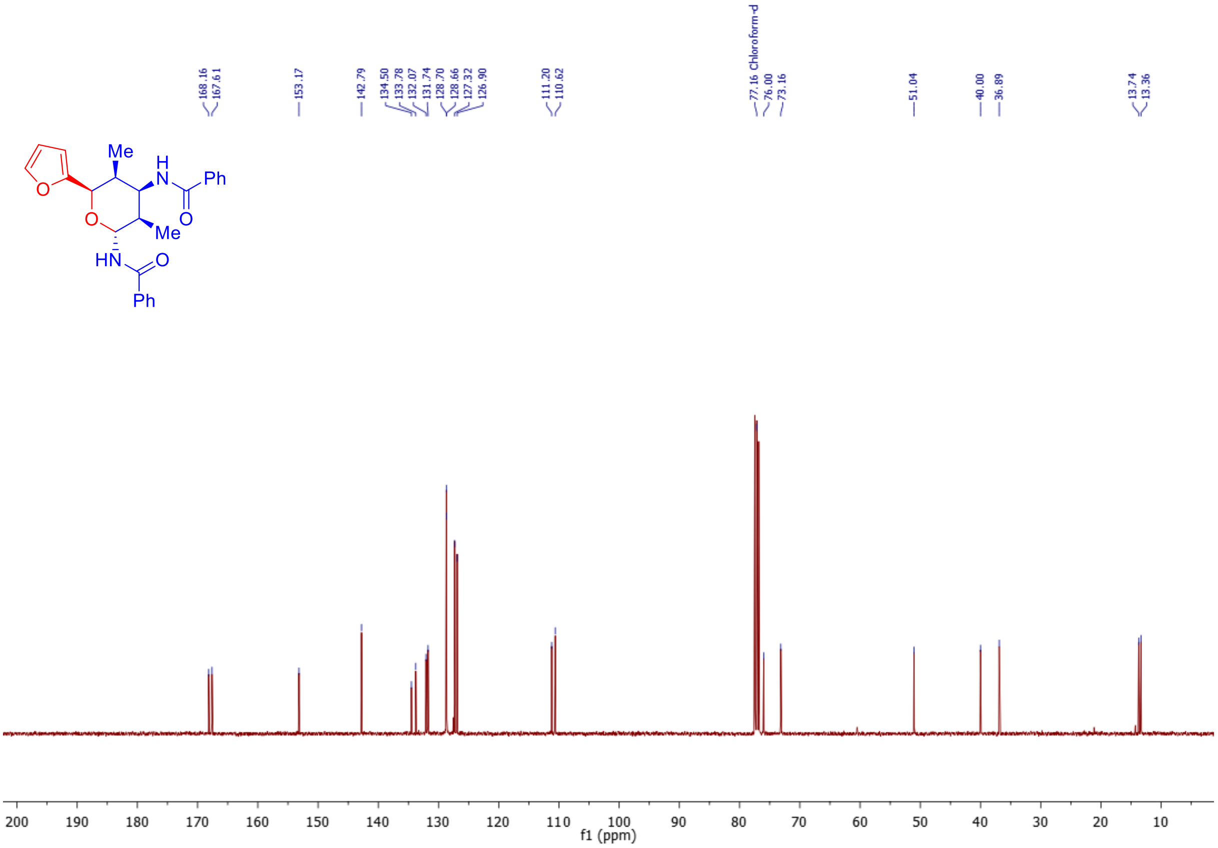

Figure 11: ^{1}H (400 MHz) and ^{13}C (126 MHz) NMR spectra of **3k** in $CDCl_3$.

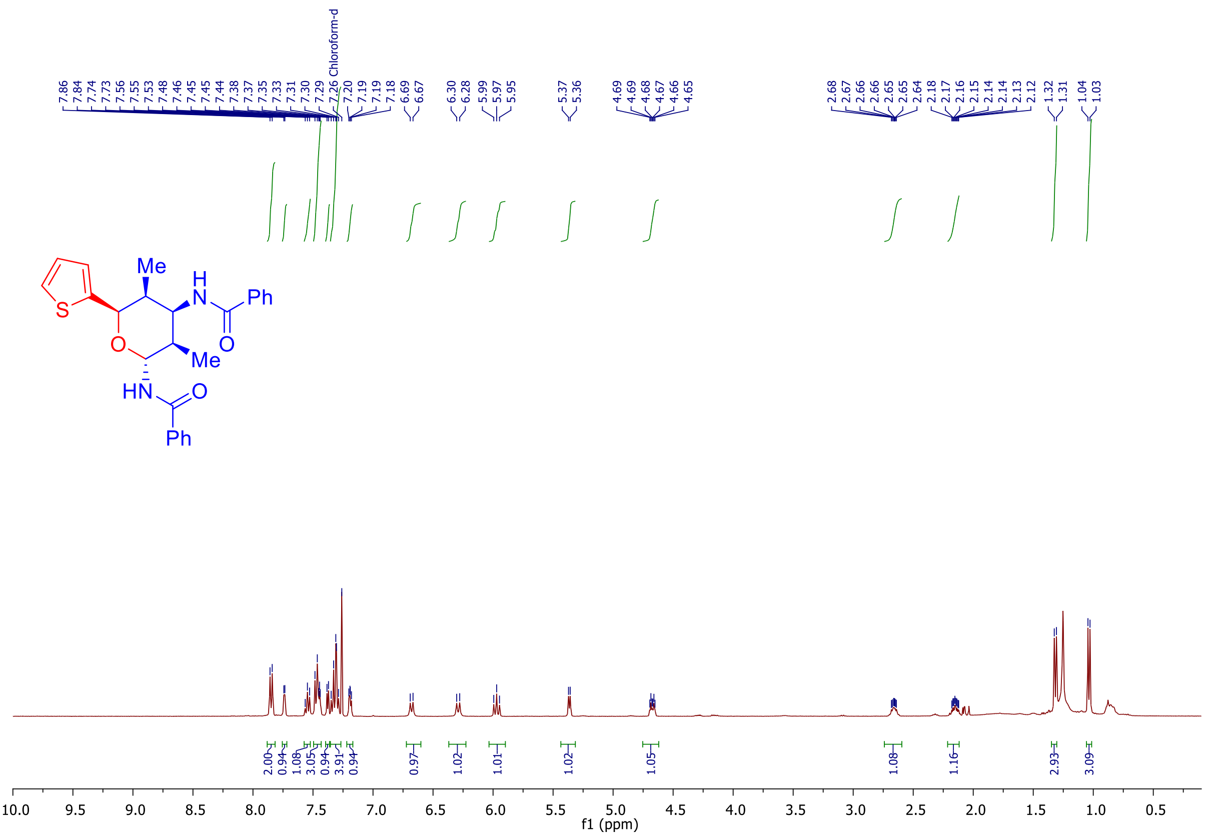

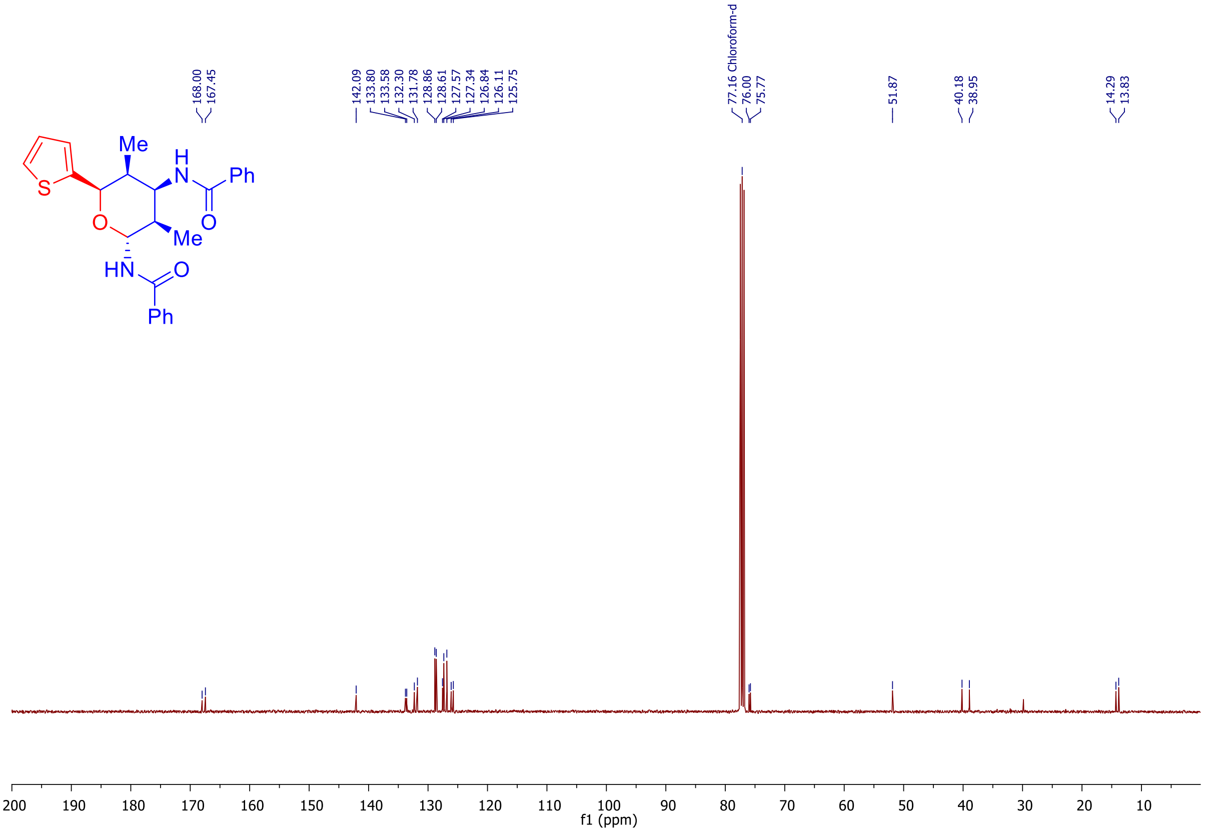

*Figure 12: ^{1}H (400 MHz) and ^{13}C (101 MHz) NMR spectra of **3l** in $CDCl_3$.*

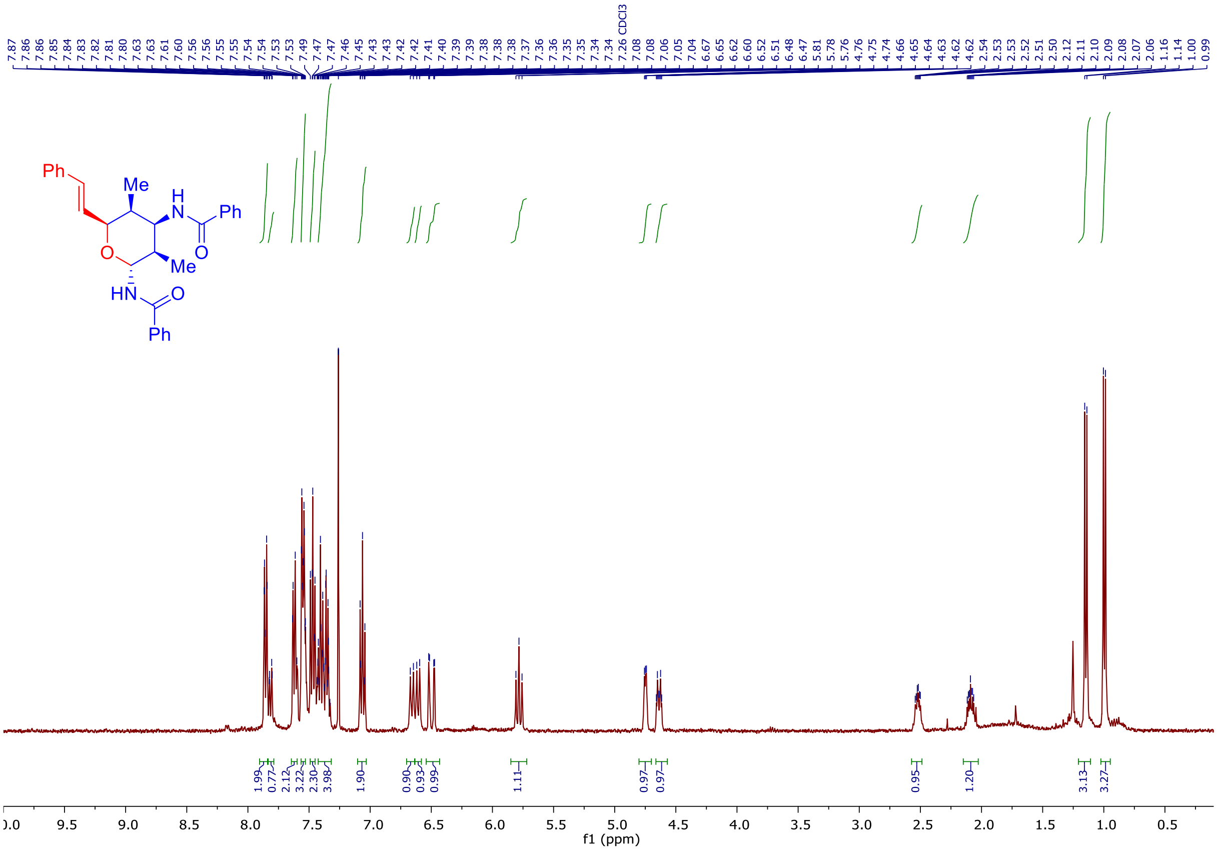

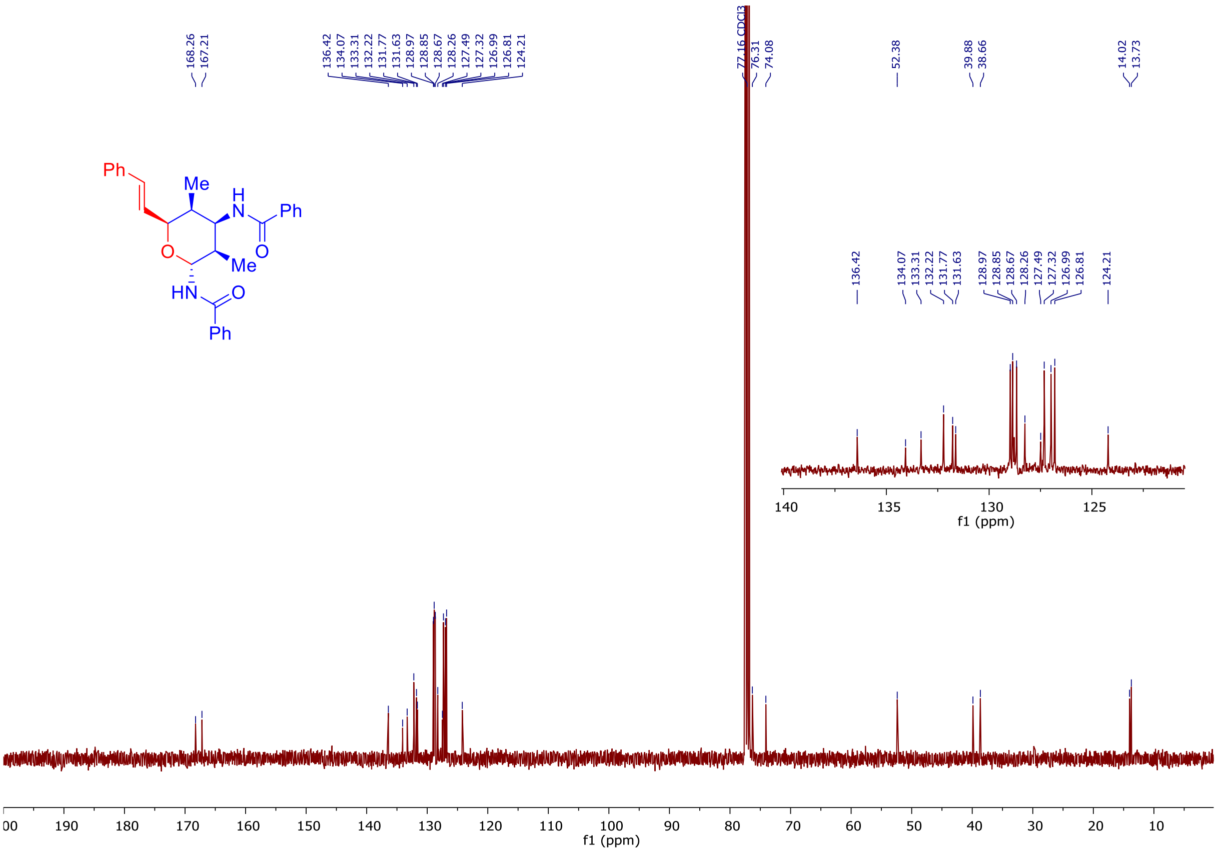

Figure 13: ^{1}H (400 MHz) and ^{13}C (101 MHz) NMR spectra of **3m** in $CDCl_3$.

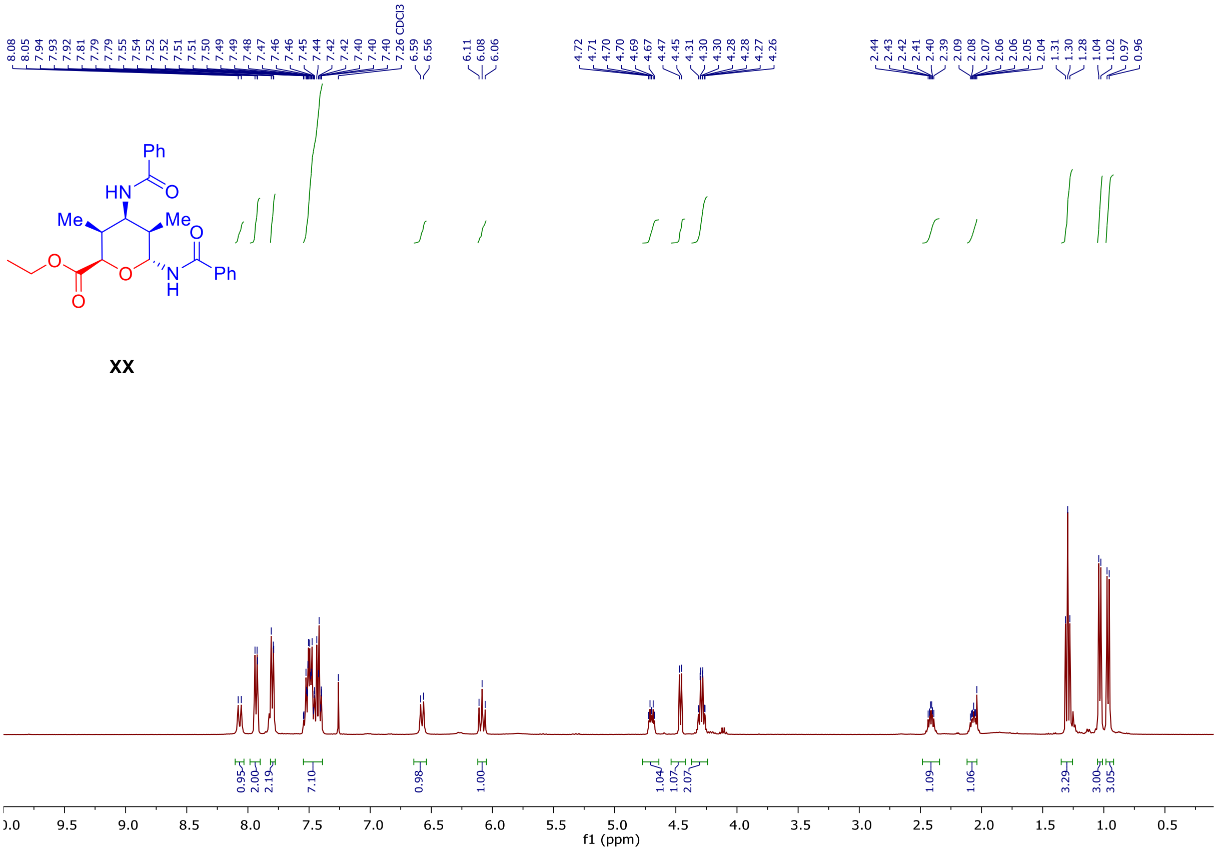

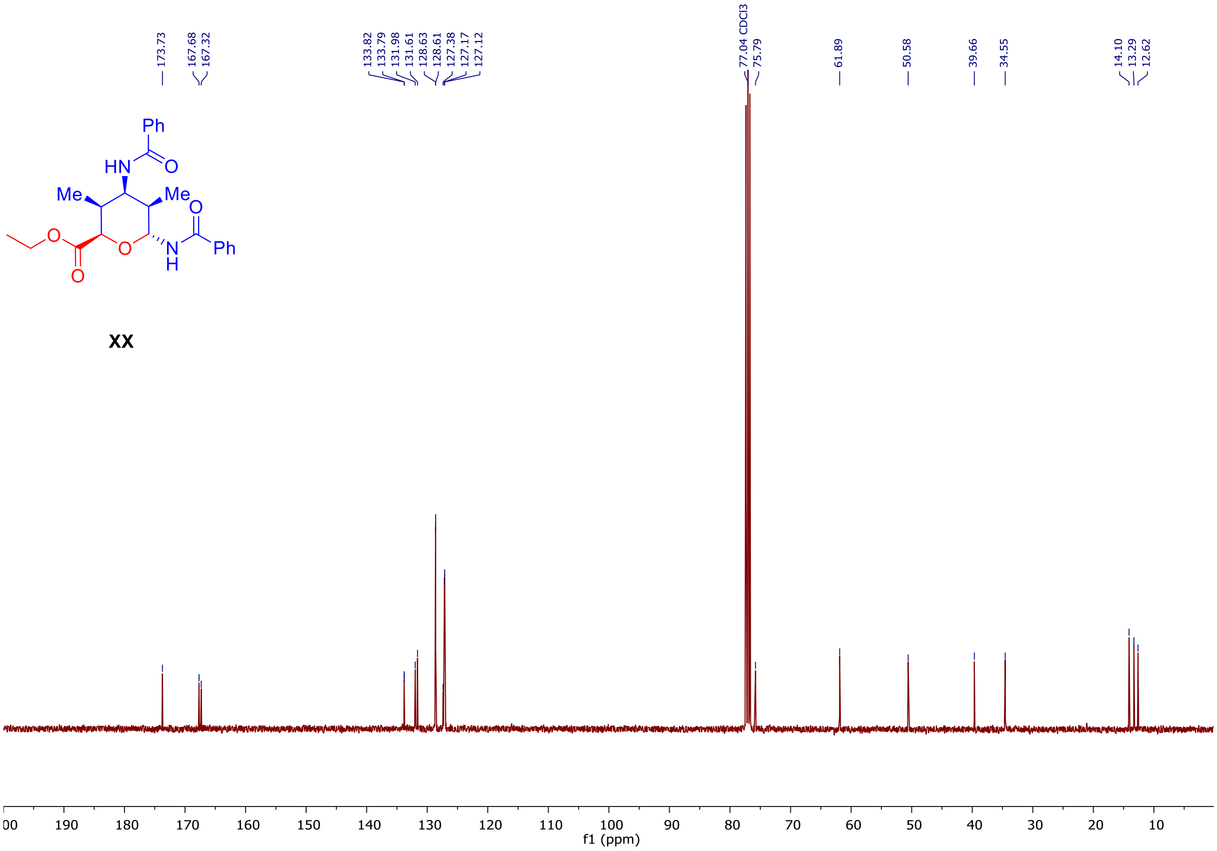

Figure 14 *^{1}H (400 MHz) and ^{13}C (101 MHz) NMR spectra of* ***3o*** *in $CDCl_3$.*

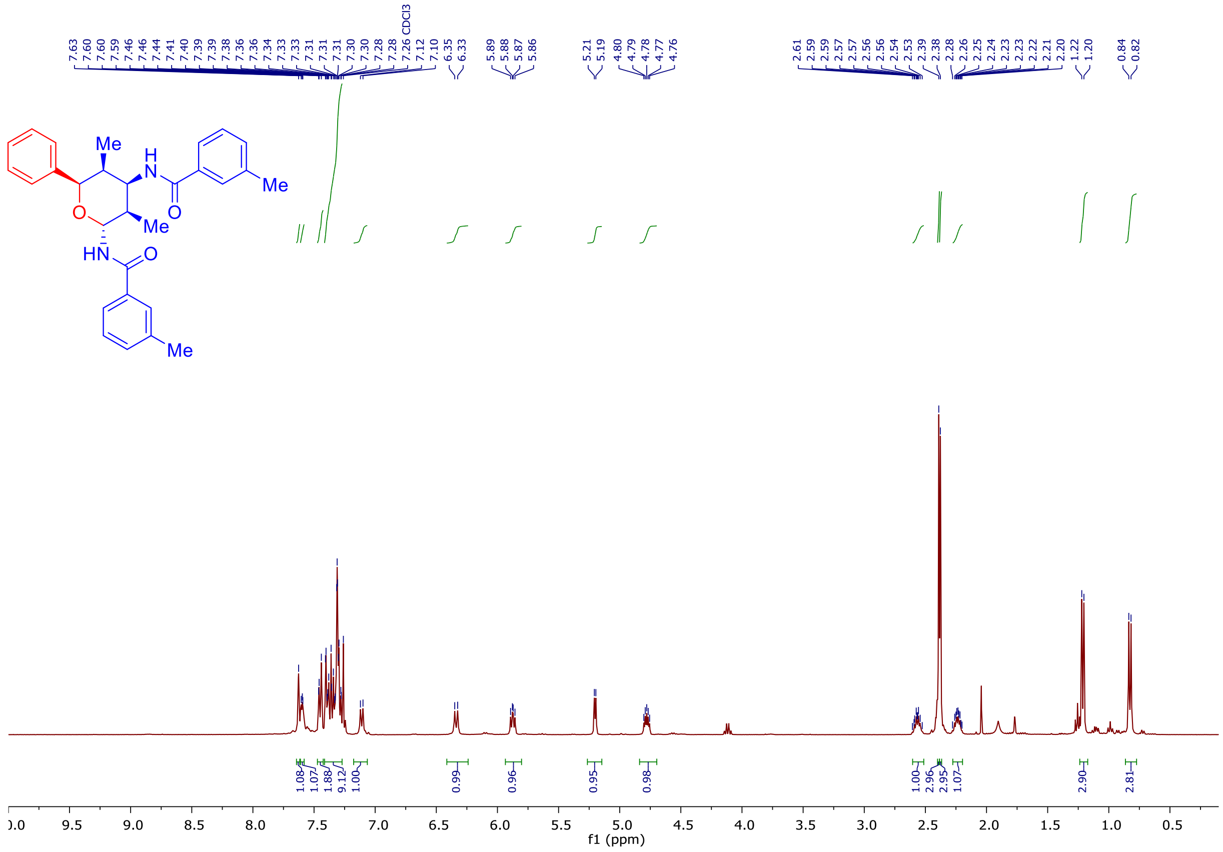

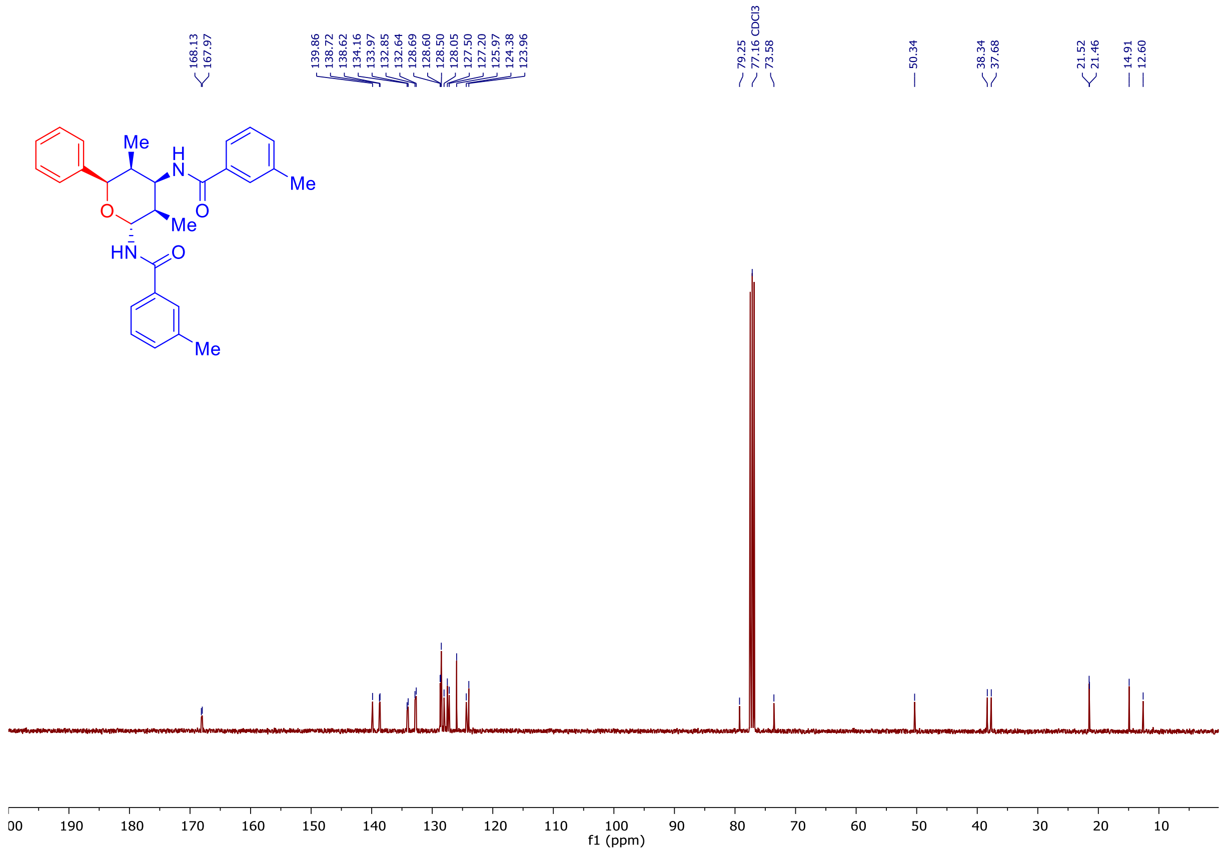

Figure 15: *^{1}H (400 MHz) and ^{13}C (101 MHz) NMR spectra of* ***3o*** *in* $CDCl_3$.

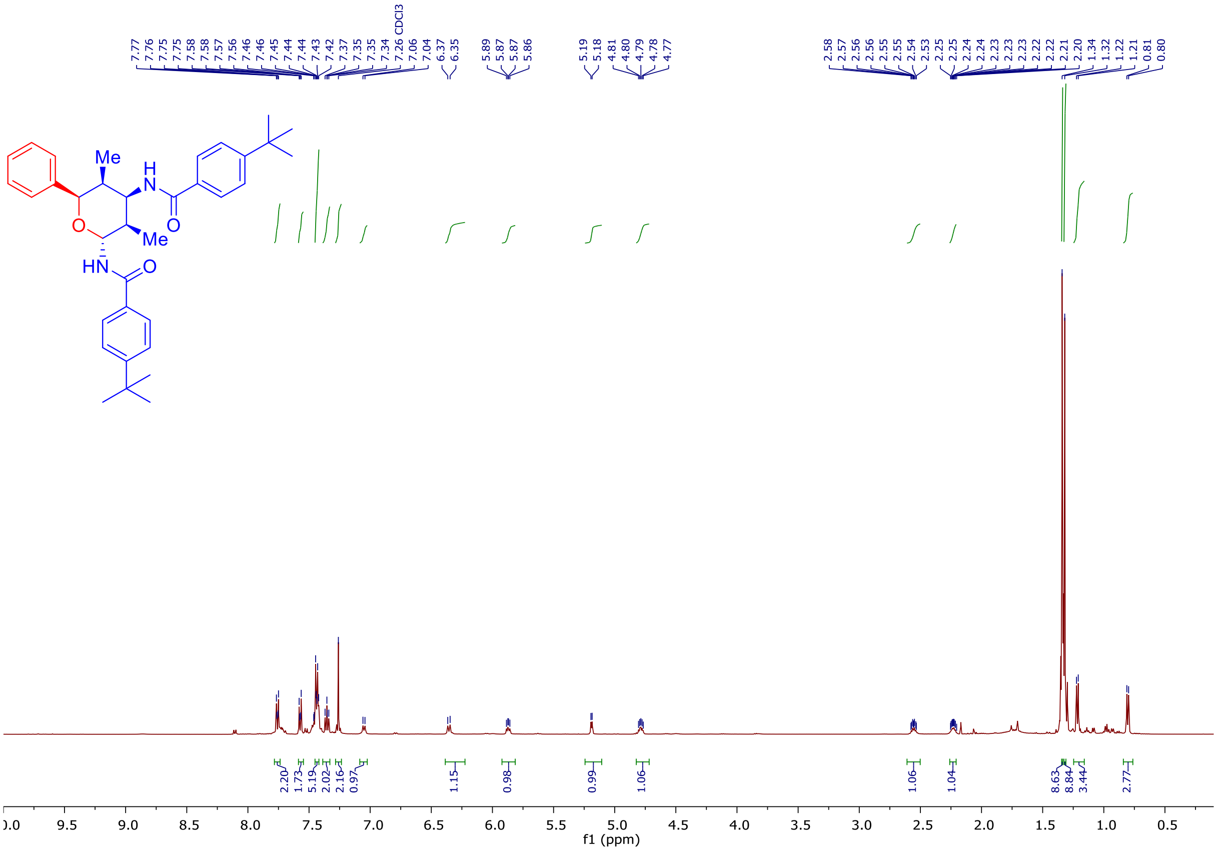

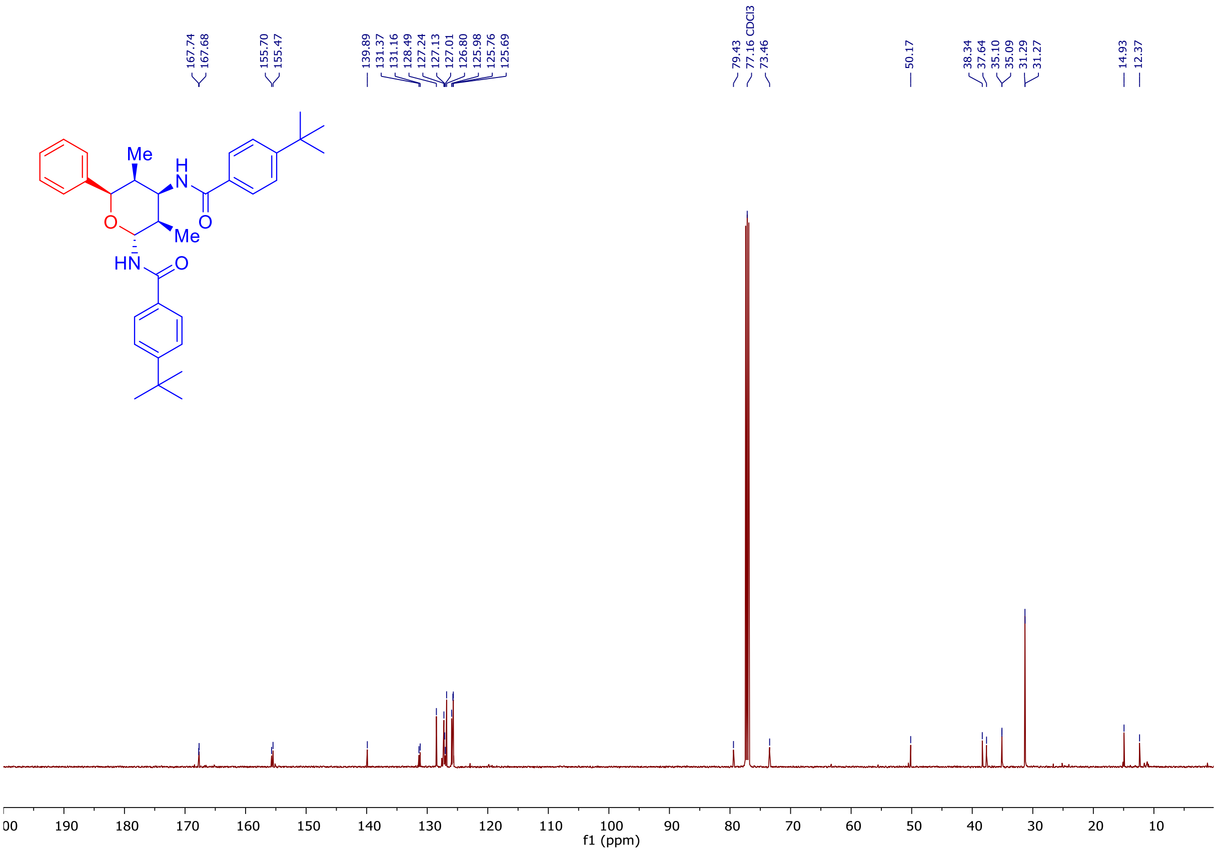

Figure 16: ^{1}H (500 MHz) and ^{13}C (126 MHz) NMR spectra of **3p** in $CDCl_3$.

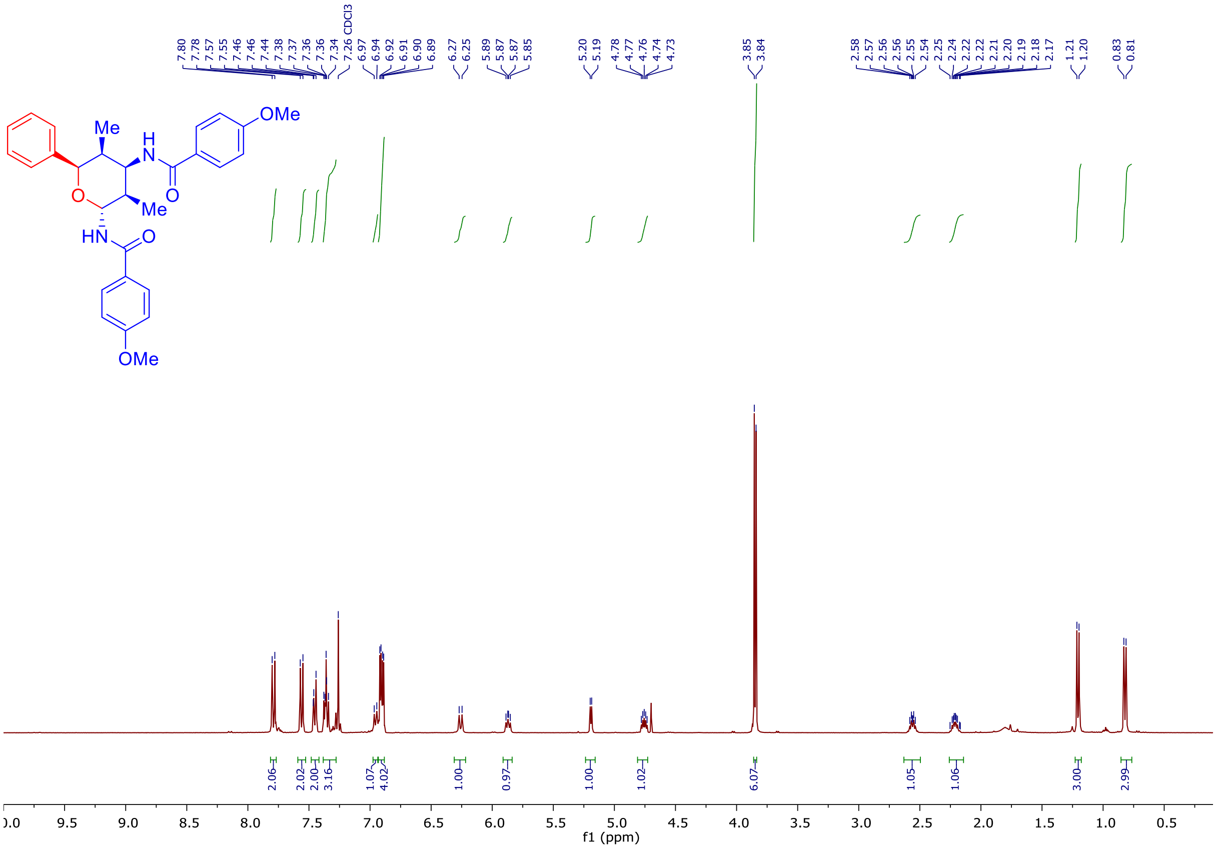

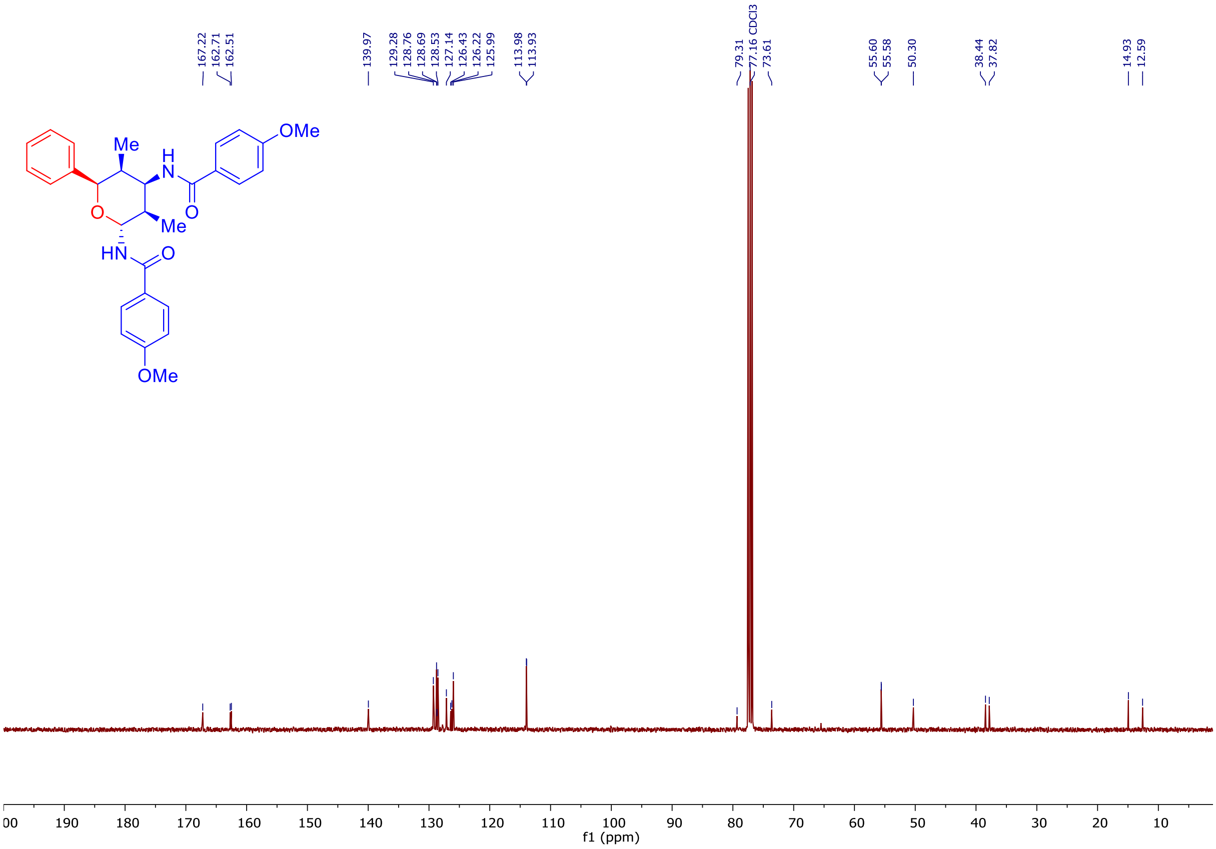

Figure 17: *^{1}H (400 MHz) and ^{13}C (101 MHz) NMR spectra of* ***3q*** *in $CDCl_3$.*

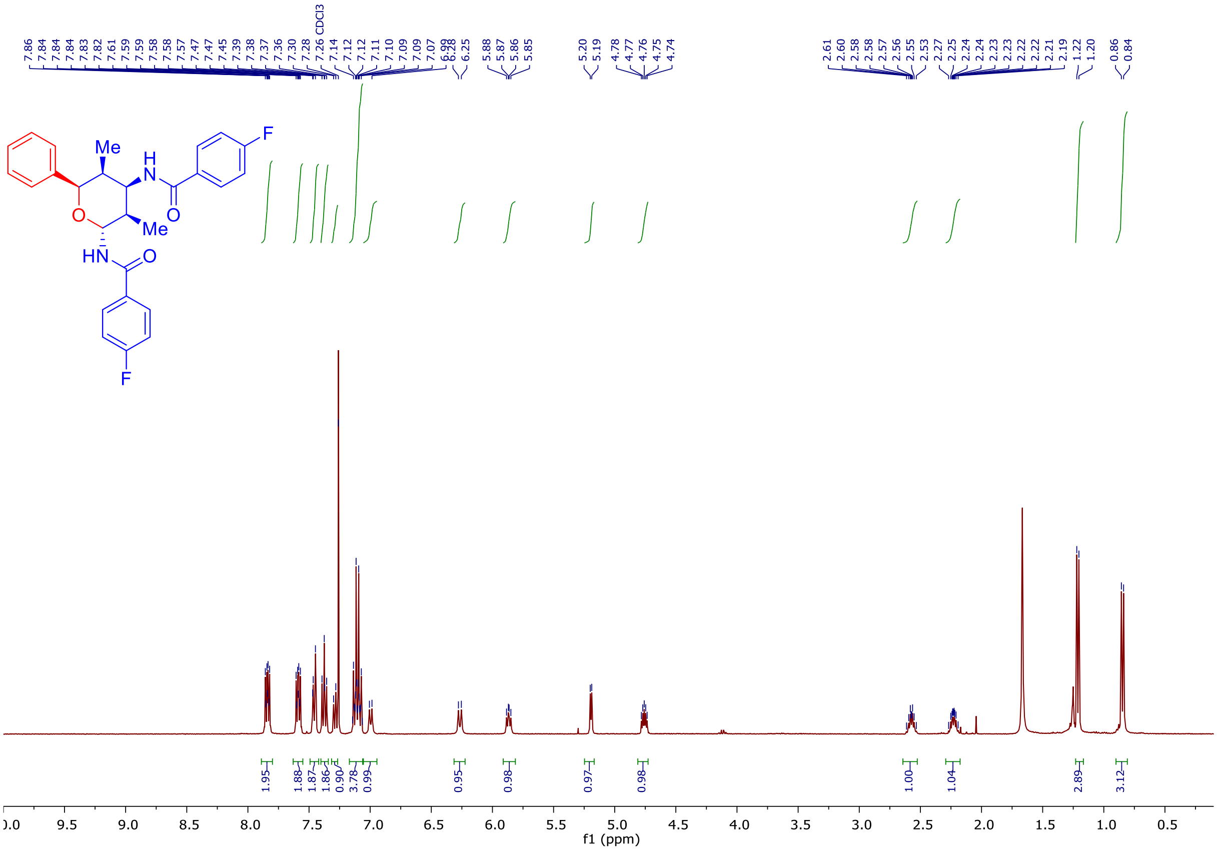
CDCl3
Me
H
N
F
O
O
Me
HN
O
F
f1 (ppm)

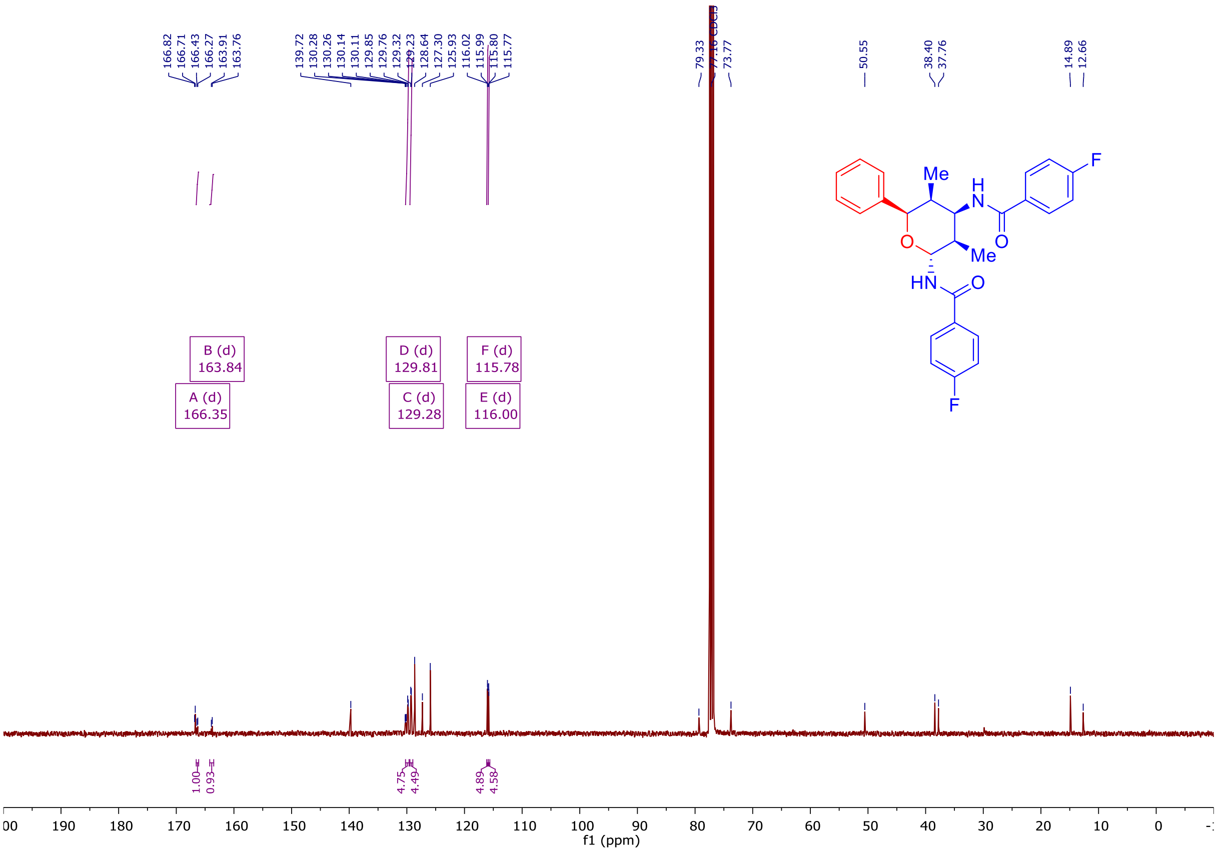
CDCl3
B (d)
163.84
A (d)
166.35
D (d)
129.81
C (d)
129.28
F (d)
115.78
E (d)
116.00
f1 (ppm)

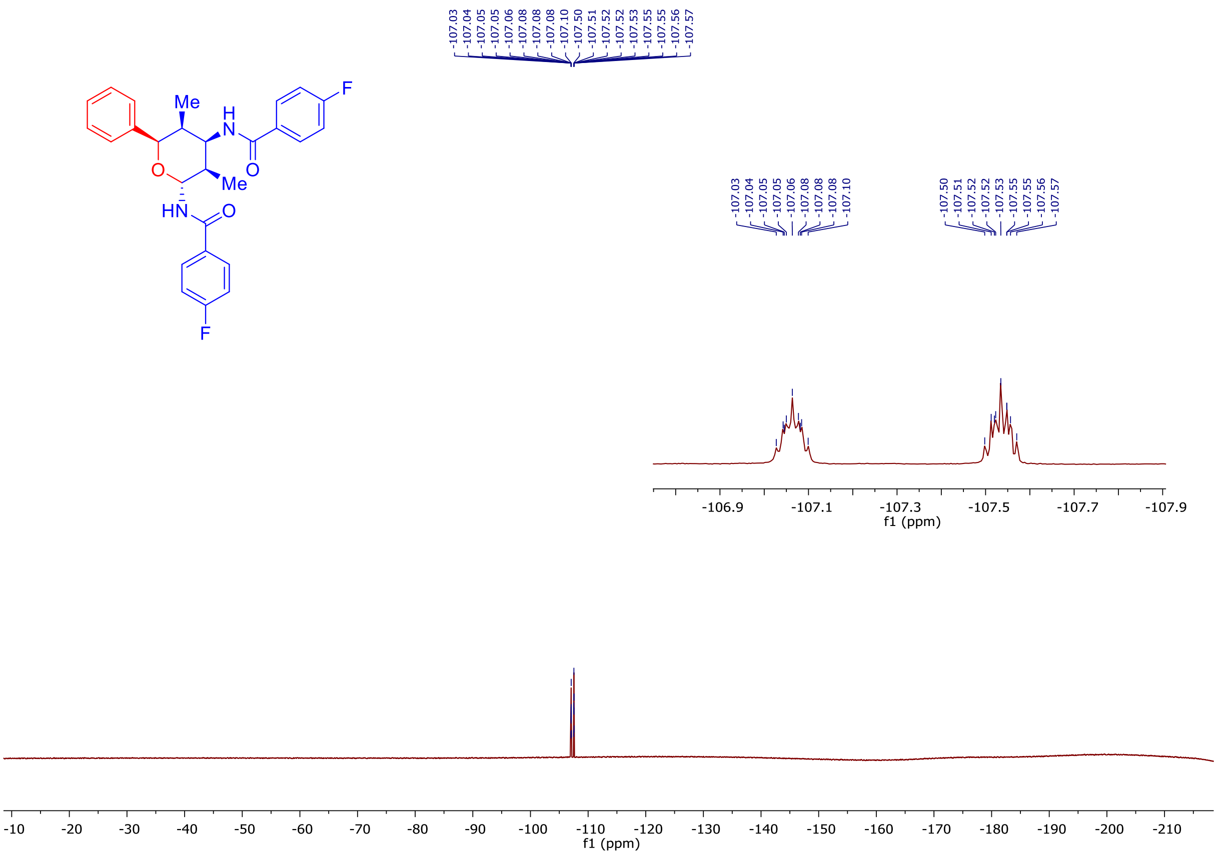

Figure 18: *^{1}H (400 MHz), ^{13}C (101 MHz) and ^{19}F (376 MHz) NMR spectra of **3r** in $CDCl_3$.*

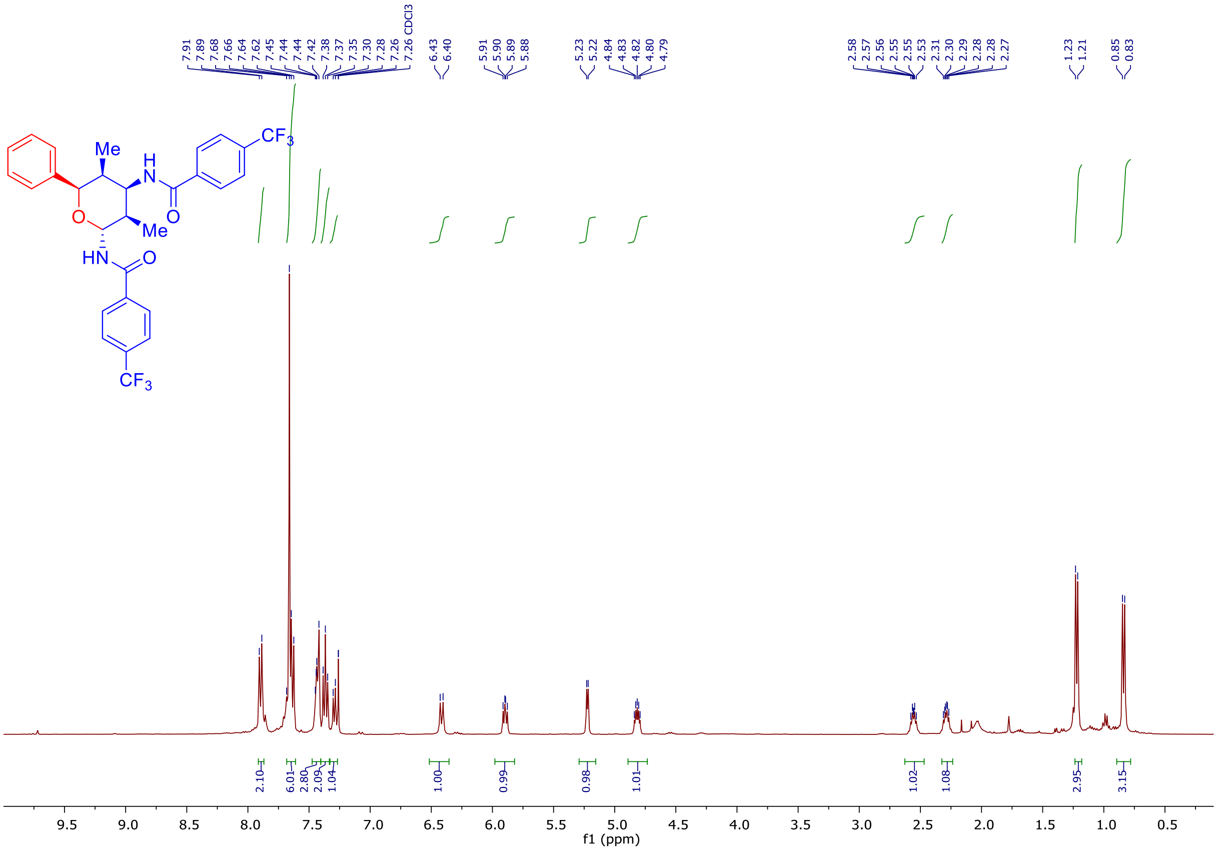

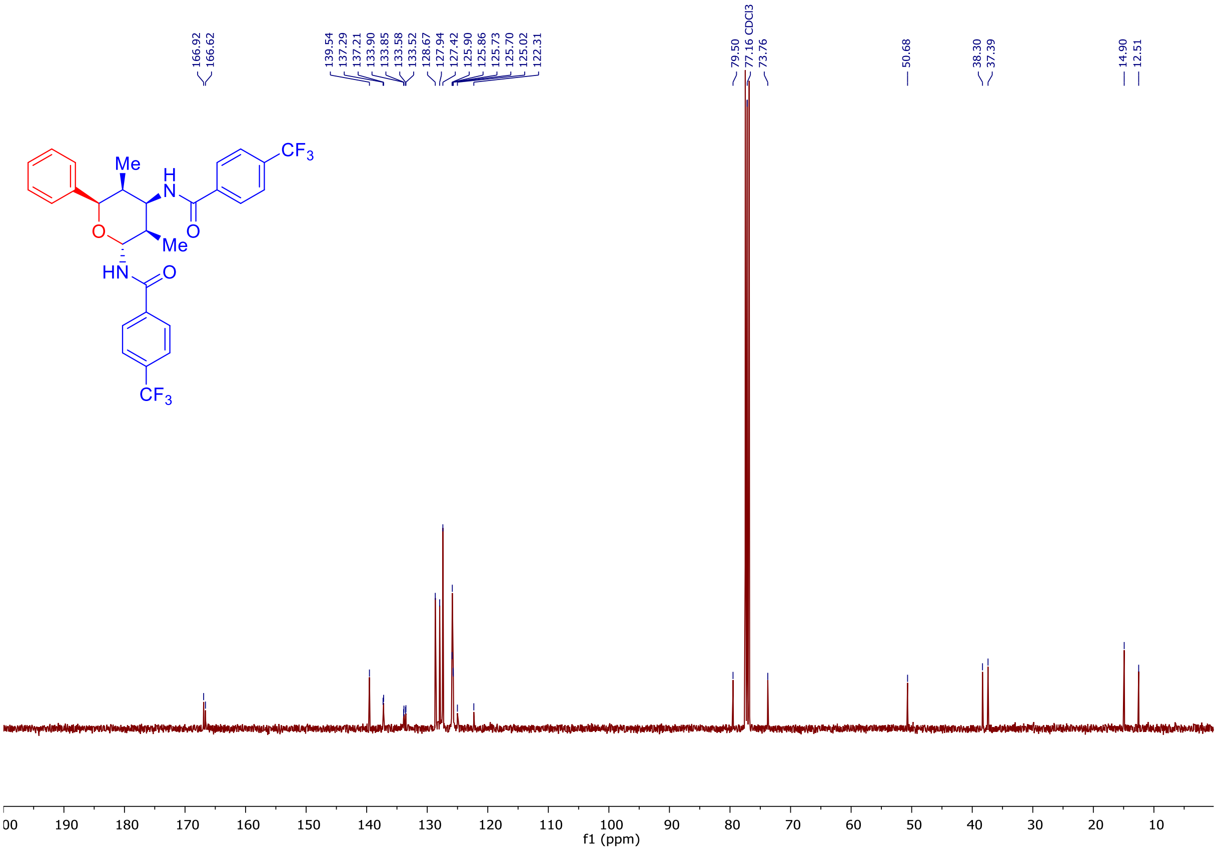

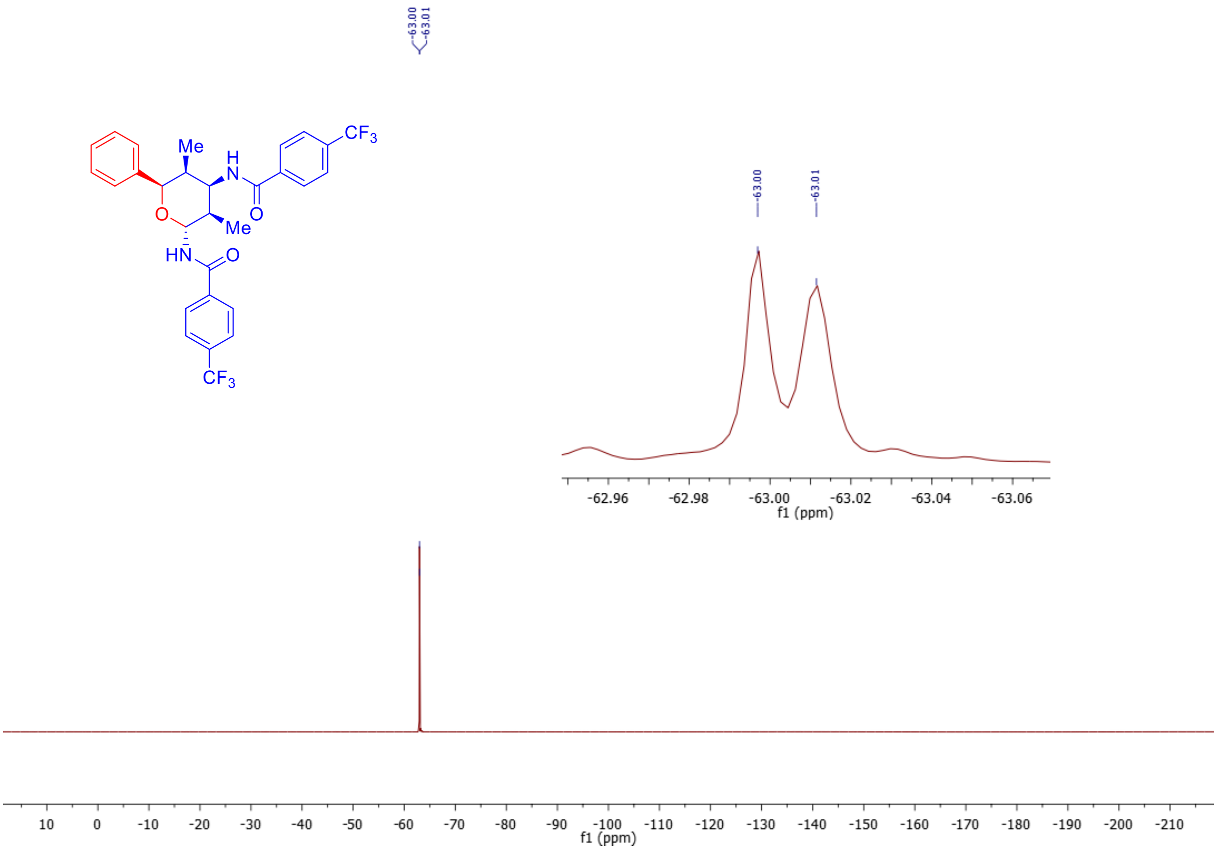

Figure 19 ^{1}H (400 MHz), ^{13}C (101 MHz) and ^{19}F (376 MHz) NMR spectra of **3s** in $CDCl_3$.

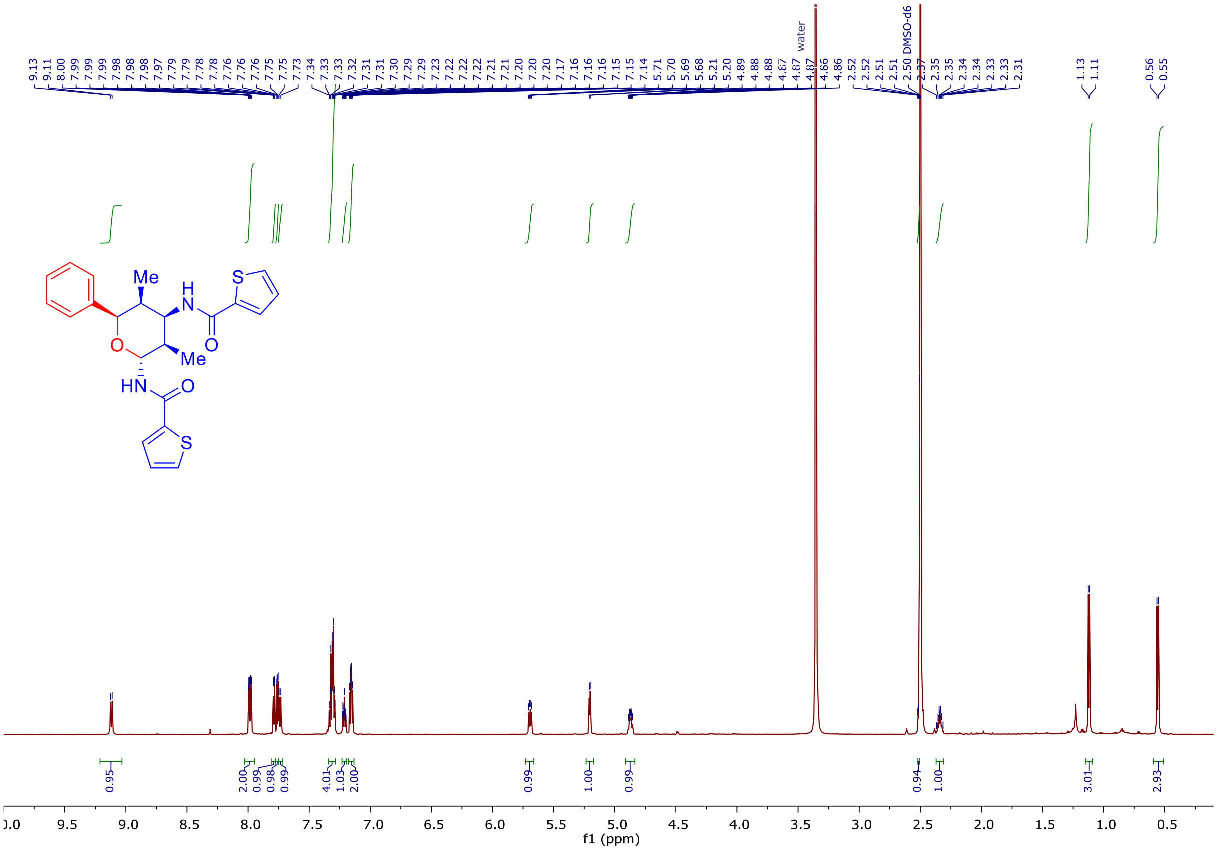

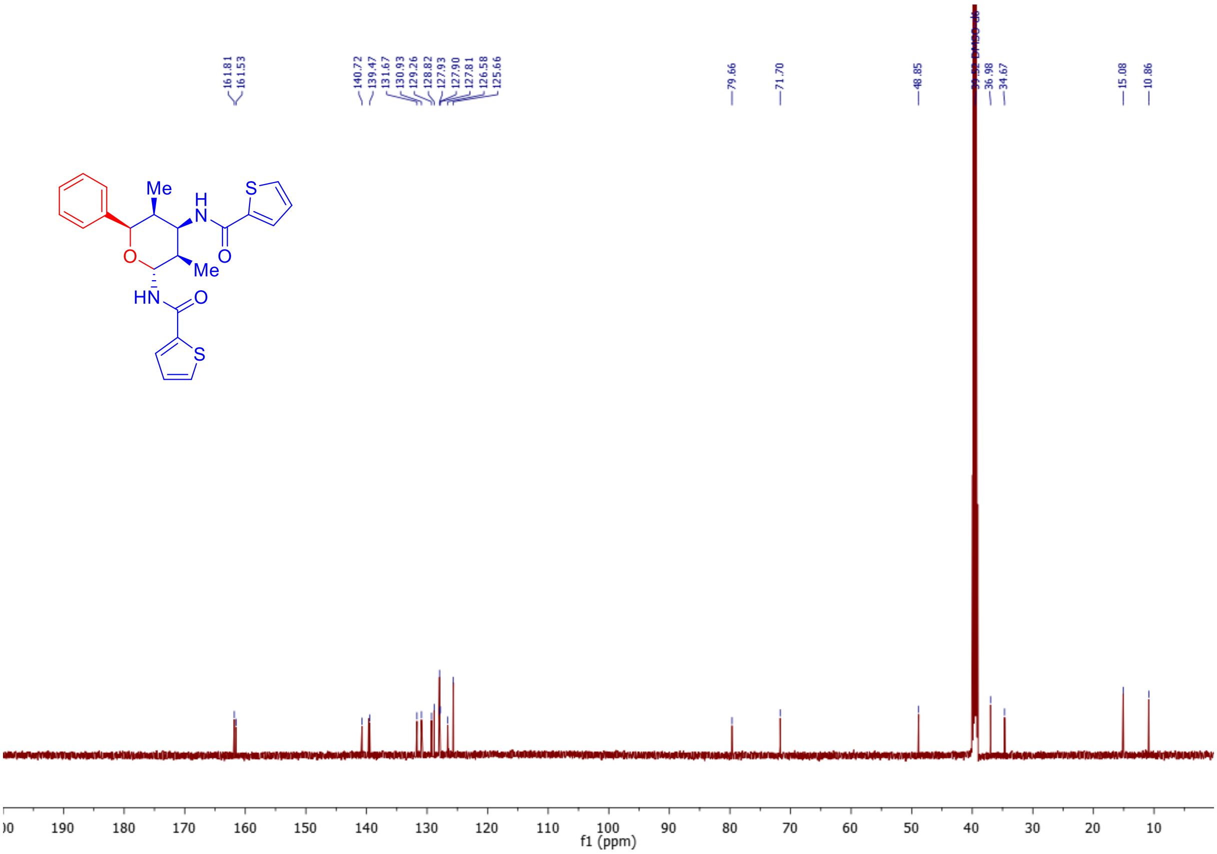

*Figure 20 ¹H (400 MHz) in $CDCl_3$ and ¹H (600 MHz), ¹³C (151 MHz) NMR spectra of **3t** in DMSO-d_6.*

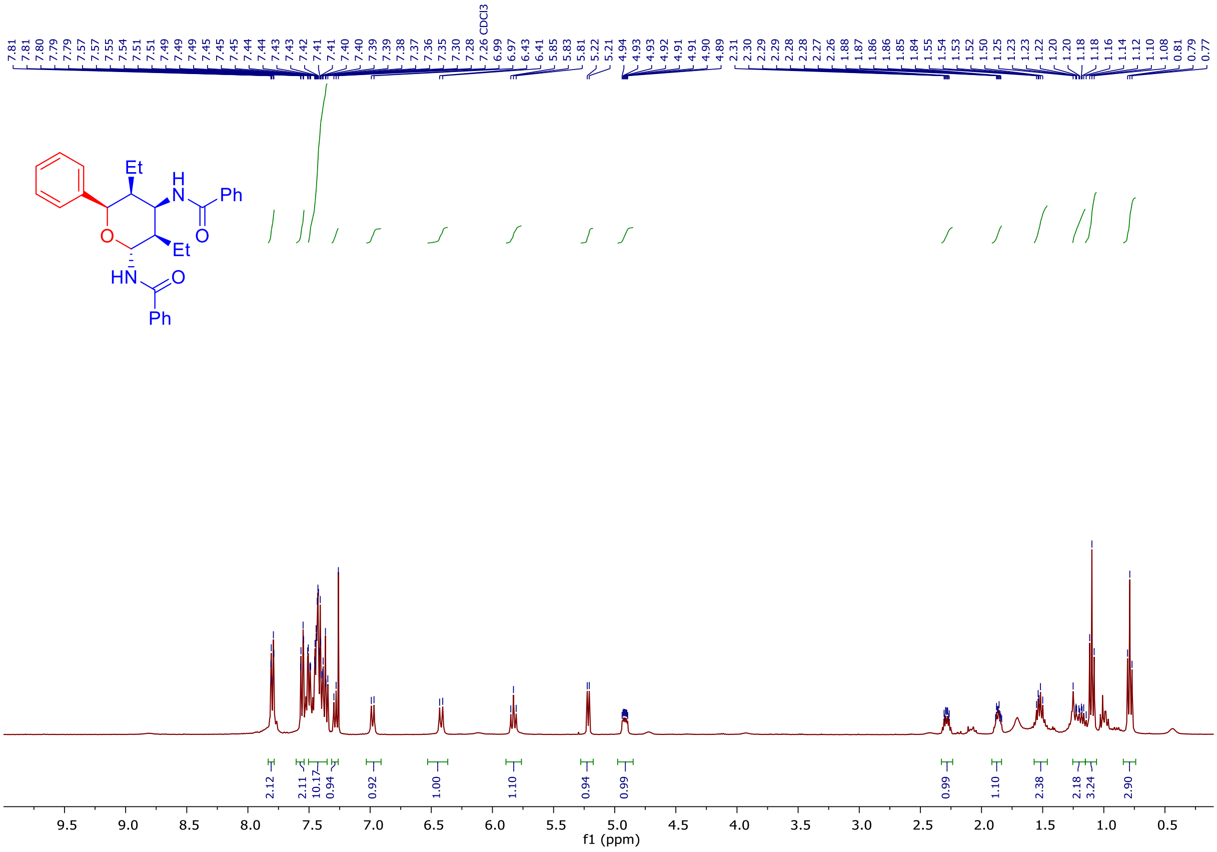

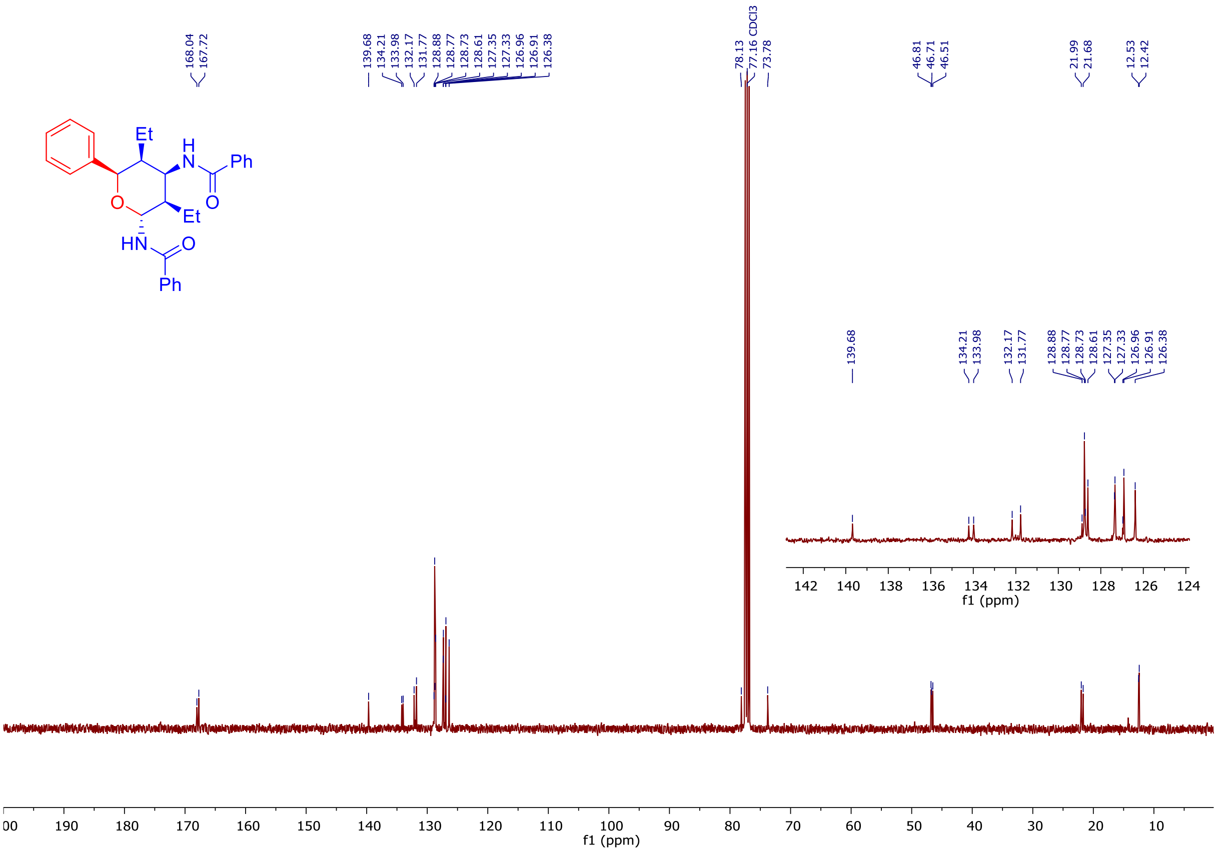

*Figure 21: ^{1}H (400 MHz) and ^{13}C (101 MHz) NMR spectra of **3u** in $CDCl_3$.*

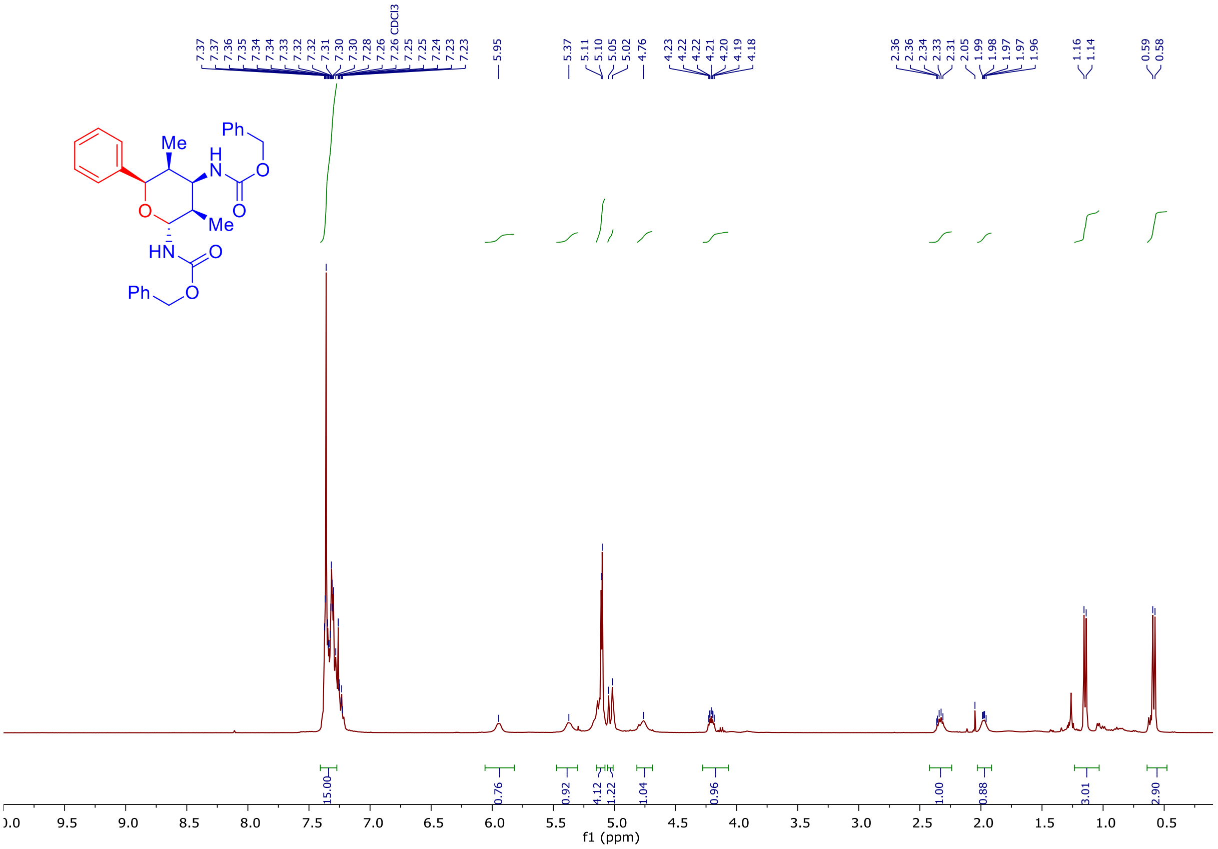

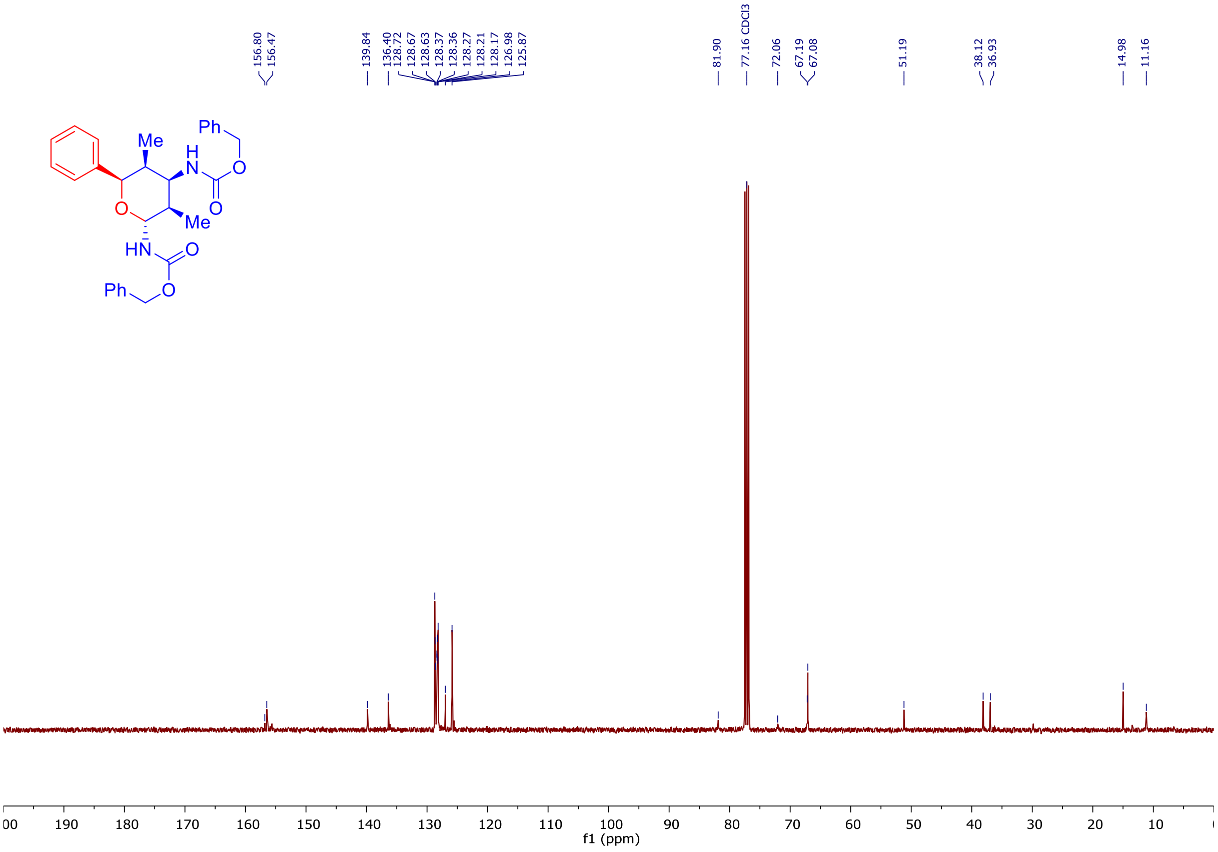

*Figure 22: ^{1}H (400 MHz) and ^{13}C (101 MHz) NMR spectra of **5a** in $CDCl_3$.*

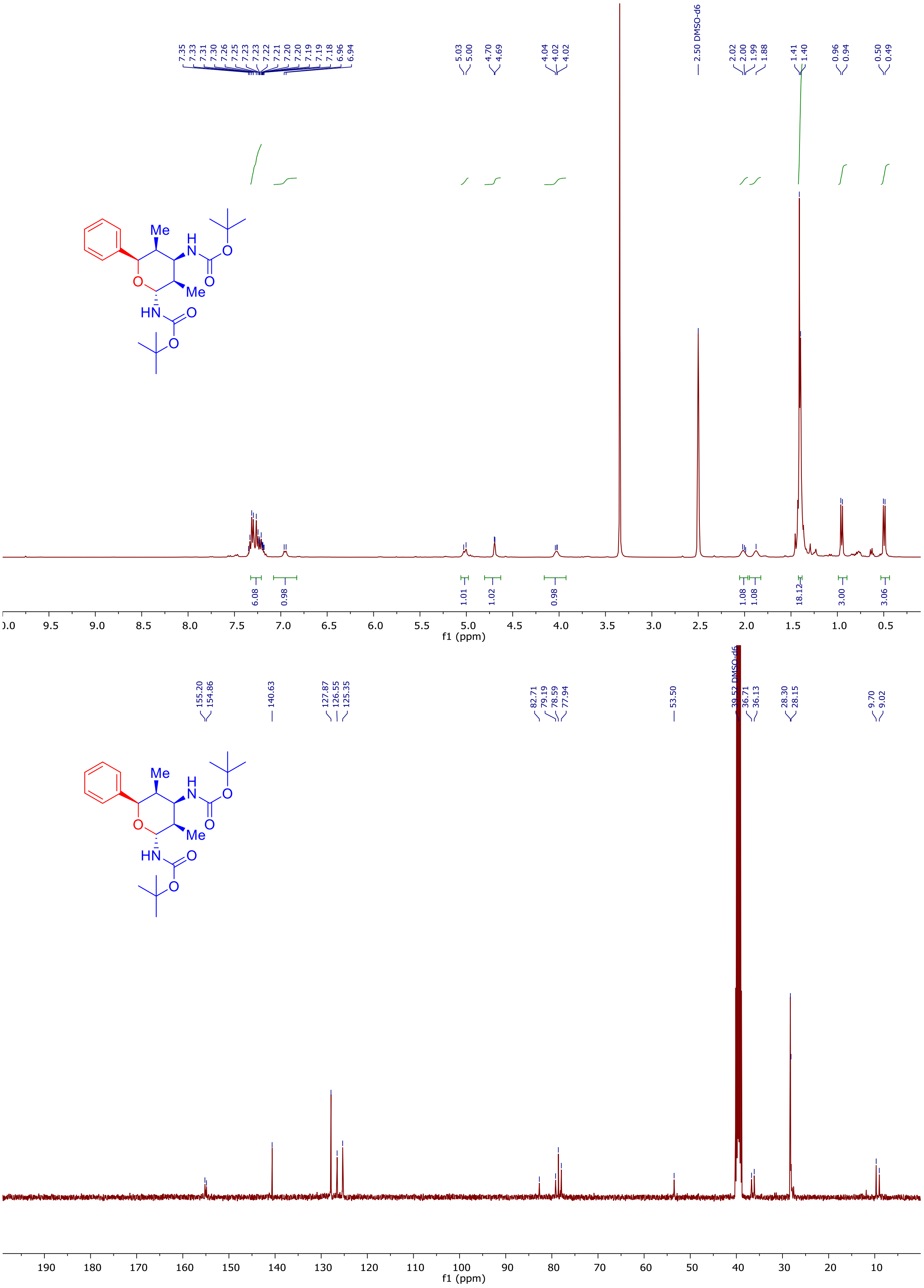

Figure 23: *^{1}H (400 MHz) and ^{13}C (101 MHz), ^{19}F NMR spectra of* ***5b*** *in DMSO-d6.*

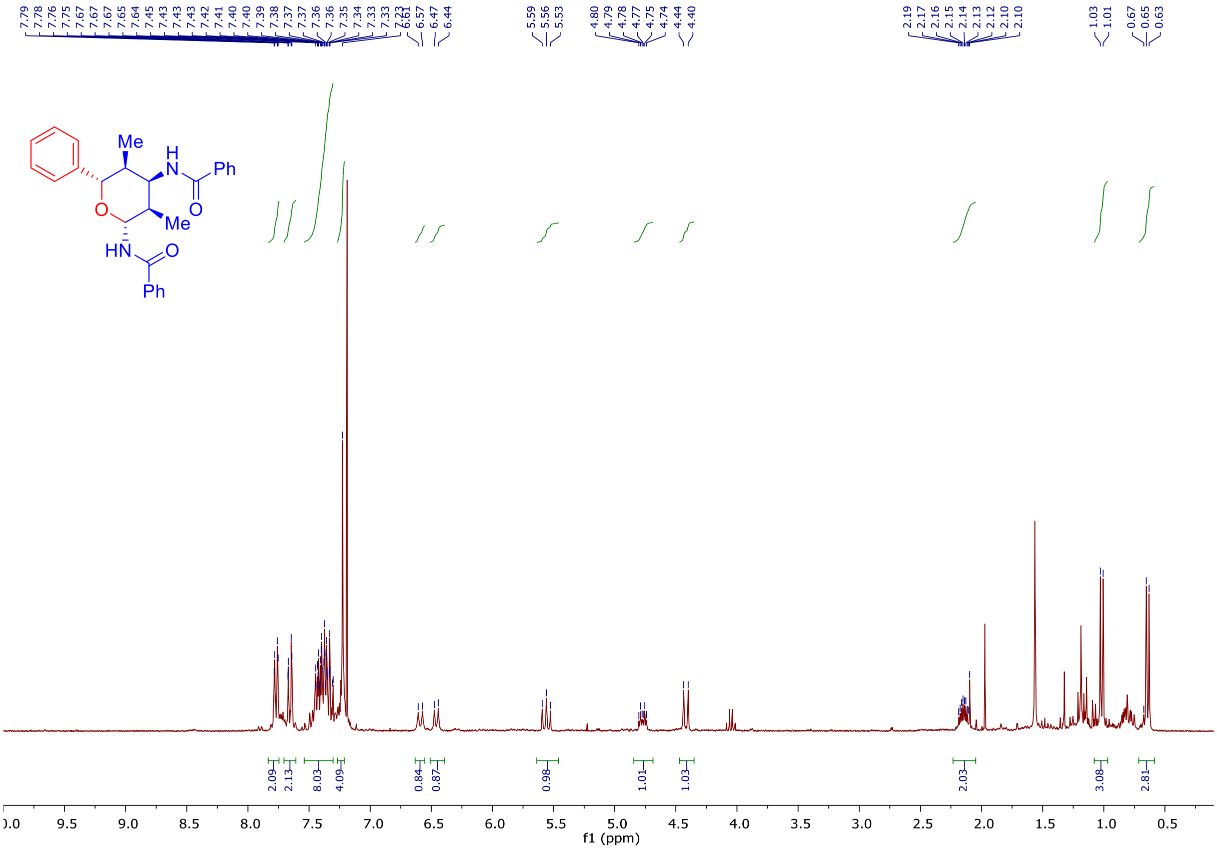

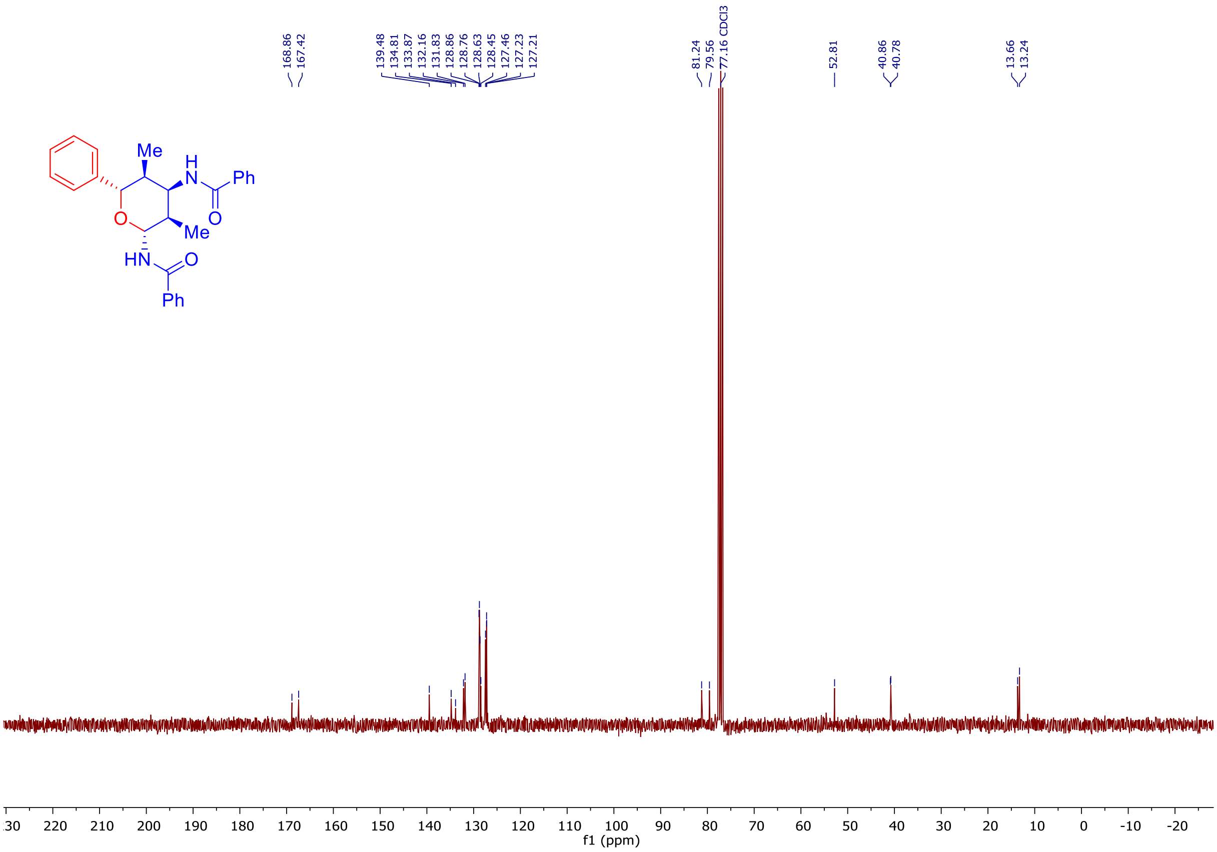

Figure 24: *^{1}H (300 MHz) and ^{13}C (75 MHz) NMR spectra of* ***6a*** *in* *$CDCl_3$.*

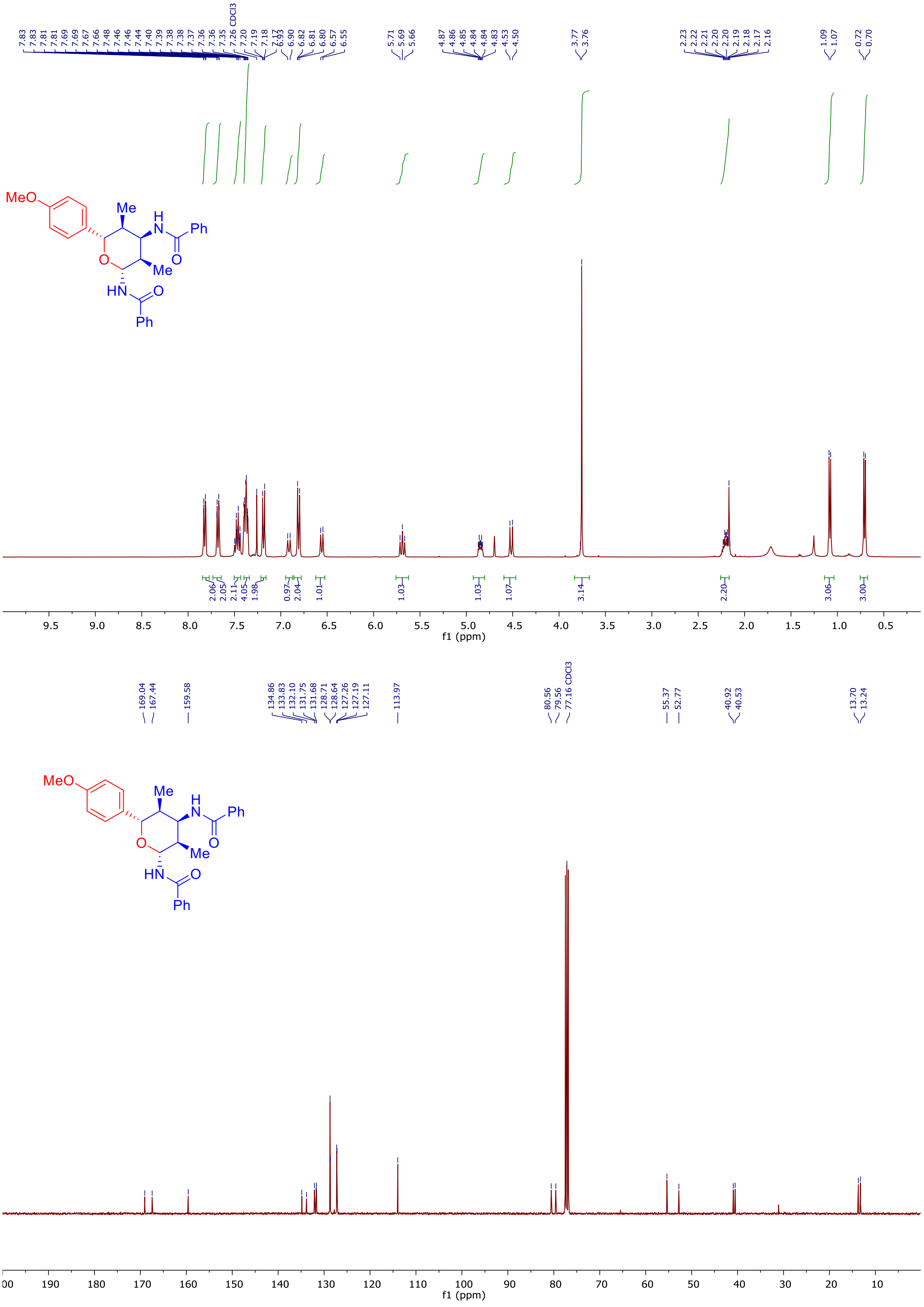

*Figure 25: ^{1}H (400 MHz) and ^{13}C (101 MHz) NMR spectra of **6c** in $CDCl_3$.*

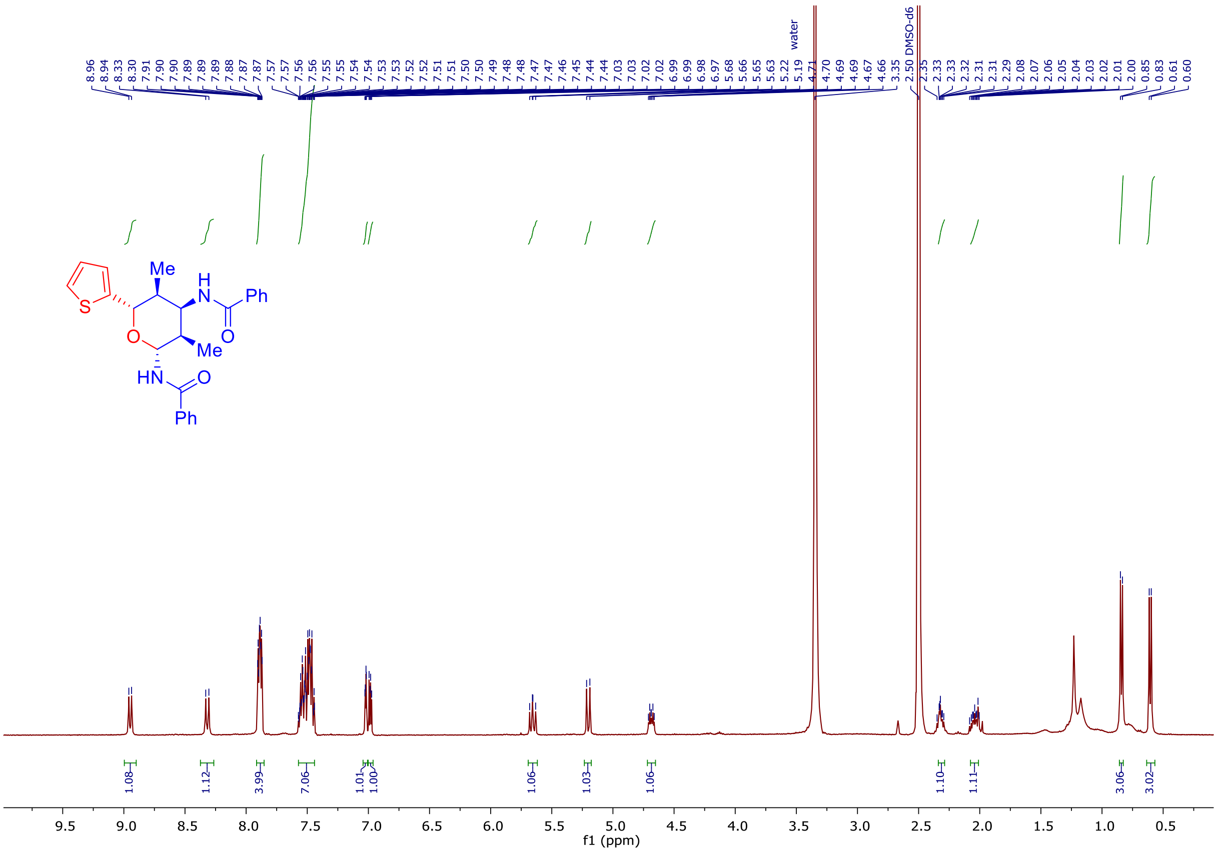

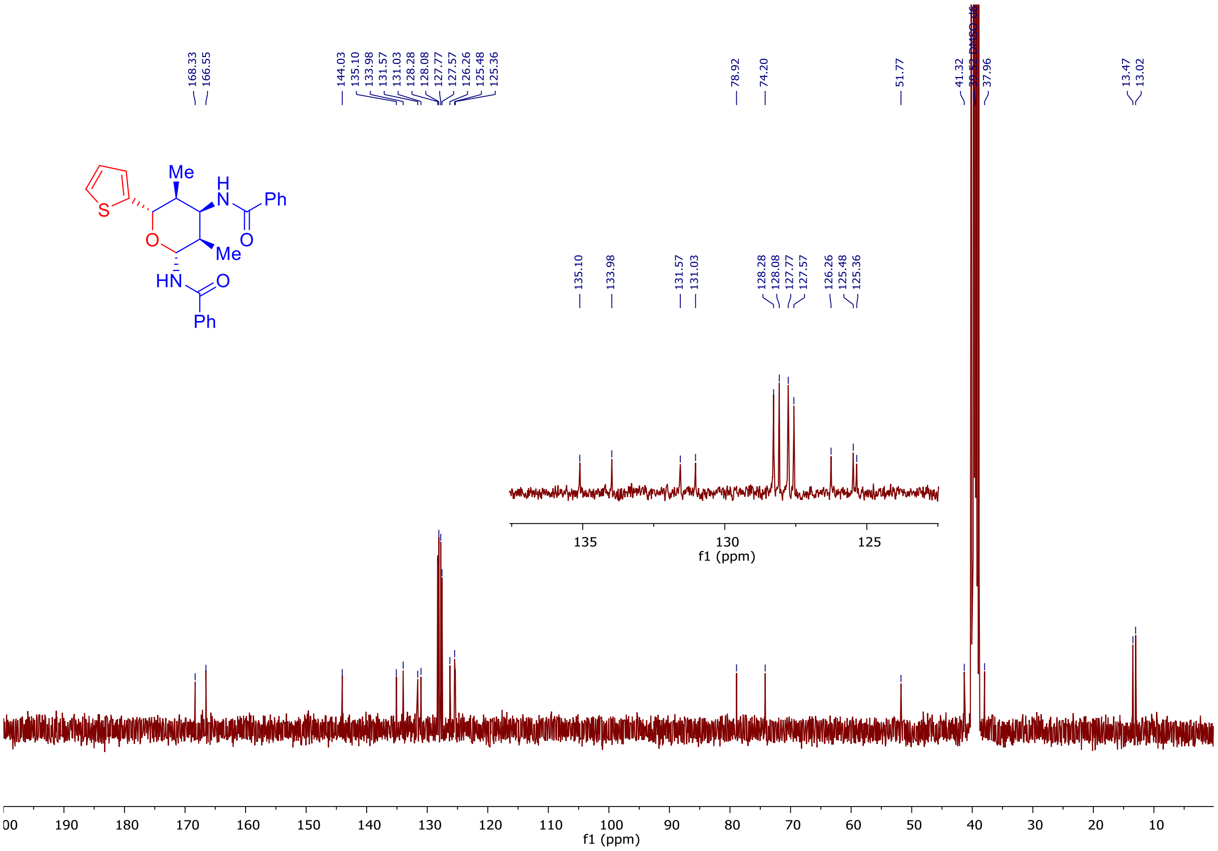

Figure 26 ^{1}H (400 MHz) and ^{13}C (101 MHz), NMR spectra of **6l** in DMSO-d_6.

4 X-ray Data

In **3a**, the H atoms bonded to N and O were freely refined. H1 forms an intramolecular N-H...π bond to the aromatic ring containing C81.

In **3e**, the coordinates of the H atoms bonded to N were refined employing a distance restraint of N-H = 0.88(1) Å. Their U values were set to $1.2U_{eq}$(N).

In **3g**, the H atoms bonded to N were freely refined. The contribution of the solvent to the scattering was subtracted using the *SQUEEZE* procedure in *PLATON* (Spek, 2009).

In **3m**, the H atoms bonded to N were freely refined. H2 forms an intramolecular N-H...π bond to the double bond C81=C82..

In **6a**, the H atoms bonded to N were freely refined. One of the chloroform molecules is disordered about an inversion centre of two equally occupied positions. The displacement parameters of the disordered atoms were restrained to an isotropic behaviour.

CCDC files 1922970- 1922974 contain the supplementary crystallographic data or this paper and can be obtained free of charge from The Cambridge Crystallographic Data Centre via www.ccdc.cam.ac.uk/data_request/cif

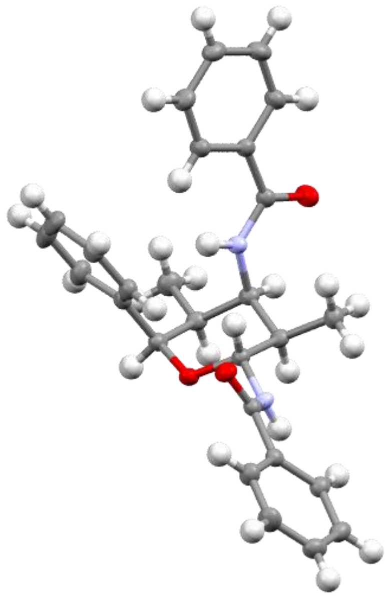

Table 1. Crystal data and structure refinement for **3a**. Displacement ellipsoids are drawn at the 50% probability level.

Identification code	CCDC 1922970	
Empirical formula	$C_{27} H_{30} N_2 O_4$	
Formula weight	446.53	
Temperature	173(2) K	
Wavelength	0.71073 Å	
Crystal system	Triclinic	
Space group	*P* -1	
Unit cell dimensions	a = 9.5626(7) Å	α= 72.725(5)°.
	b = 10.3929(7) Å	β= 84.615(6)°.
	c = 12.2195(9) Å	γ = 89.449(6)°.
Volume	1154.30(15) $Å^3$	
Z	2	
Density (calculated)	1.285 Mg/m^3	
Absorption coefficient	0.086 mm^{-1}	
F(000)	476	
Crystal size	0.260 x 0.260 x 0.210 mm^3	
Theta range for data collection	3.498 to 27.170°.	
Index ranges	-12<=h<=12, -13<=k<=13, -15<=l<=15	
Reflections collected	17254	
Independent reflections	5066 [R(int) = 0.0239]	
Completeness to theta = 25.000°	99.7 %	
Absorption correction	Semi-empirical from equivalents	
Max. and min. transmission	1.000 and 0.670	
Refinement method	Full-matrix least-squares on F^2	
Data / restraints / parameters	5066 / 0 / 314	
Goodness-of-fit on F^2	1.035	
Final R indices [I>2sigma(I)]	R1 = 0.0417, wR2 = 0.0933	
R indices (all data)	R1 = 0.0589, wR2 = 0.0997	
Largest diff. peak and hole	0.234 and -0.182 e.$Å^{-3}$	

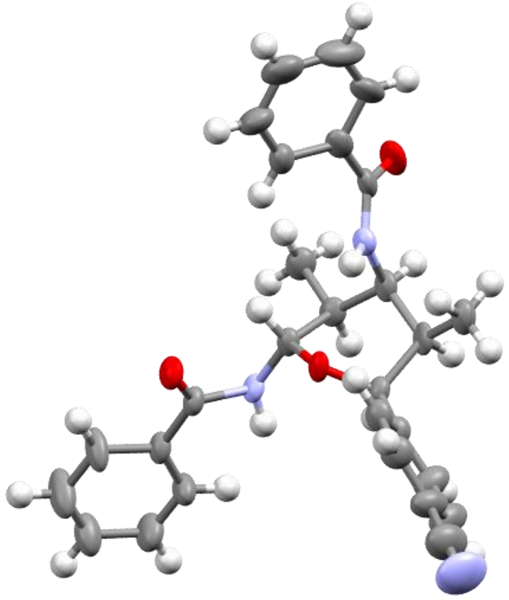

Table 2. Crystal data and structure refinement for pyrane **3e**. Displacement ellipsoids are drawn at the 50% probability level.

Identification code	CCDC 1922971	
Empirical formula	$C_{28} H_{27} N_3 O_3$	
Formula weight	453.52	
Temperature	173(2) K	
Wavelength	0.71073 Å	
Crystal system	Monoclinic	
Space group	*C* 2/*c*	
Unit cell dimensions	a = 24.416(3) Å	α= 90°.
	b = 13.6344(18) Å	β= 116.117(7)°.
	c = 16.8544(16) Å	γ = 90°.
Volume	5037.9(11) $Å^3$	
Z	8	
Density (calculated)	1.196 Mg/m^3	
Absorption coefficient	0.079 mm^{-1}	
F(000)	1920	
Crystal size	0.190 x 0.170 x 0.090 mm^3	
Theta range for data collection	3.463 to 25.025°.	
Index ranges	-29<=h<=24, -16<=k<=16, -20<=l<=20	
Reflections collected	21933	
Independent reflections	4440 [R(int) = 0.1076]	
Completeness to theta = 25.000°	99.6 %	
Absorption correction	Semi-empirical from equivalents	
Max. and min. transmission	1.000 and 0.662	
Refinement method	Full-matrix least-squares on F^2	
Data / restraints / parameters	4440 / 2 / 313	
Goodness-of-fit on F^2	1.837	
Final R indices [I>2sigma(I)]	R1 = 0.1644, wR2 = 0.3486	
R indices (all data)	R1 = 0.2045, wR2 = 0.3631	
Largest diff. peak and hole	0.961 and -0.748 e.$Å^{-3}$	

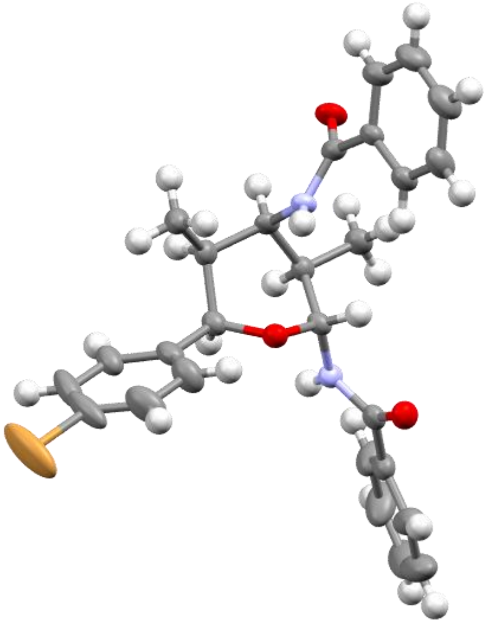

Table 3. Crystal data and structure refinement for **3g**. Displacement ellipsoids are drawn at the 50% probability level.

Identification code	CCDC 1922972	
Empirical formula	$C_{27} H_{27} Br N_2 O_3$	
Formula weight	507.41	
Temperature	173(2) K	
Wavelength	0.71073 Å	
Crystal system	Monoclinic	
Space group	*P* 21/*n*	
Unit cell dimensions	a = 14.2053(9) Å	α = 90°.
	b = 13.4090(11) Å	β = 114.138(5)°.
	c = 15.9188(11) Å	γ = 90°.
Volume	2767.1(4) $Å^3$	
Z	4	
Density (calculated)	1.218 Mg/m^3	
Absorption coefficient	1.513 mm^{-1}	
F(000)	1048	
Crystal size	0.240 x 0.140 x 0.130 mm^3	
Theta range for data collection	3.347 to 25.024°.	
Index ranges	-16<=h<=16, -15<=k<=15, -18<=l<=18	
Reflections collected	28945	
Independent reflections	4863 [R(int) = 0.0572]	
Completeness to theta = 25.000°	99.7 %	
Absorption correction	Semi-empirical from equivalents	
Max. and min. transmission	1.000 and 0.403	
Refinement method	Full-matrix least-squares on F^2	
Data / restraints / parameters	4863 / 0 / 306	
Goodness-of-fit on F^2	1.079	
Final R indices [I>2sigma(I)]	R1 = 0.0693, wR2 = 0.1728	
R indices (all data)	R1 = 0.1031, wR2 = 0.1904	
Largest diff. peak and hole	0.646 and -0.918 e.$Å^{-3}$	

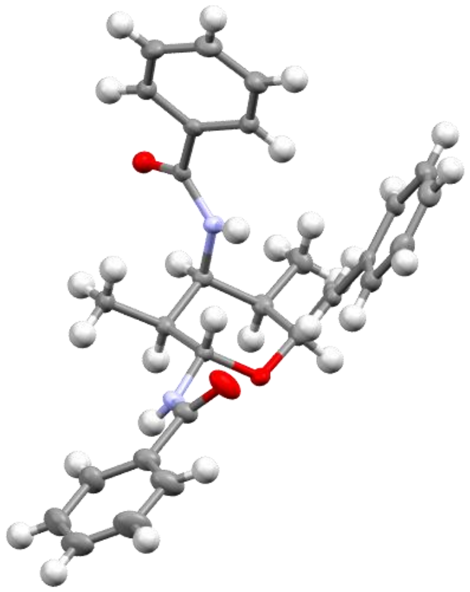

Table 4. Crystal data and structure refinement for pyrane **3m**. Displacement ellipsoids are drawn at the 50% probability level.

Identification code	CCDC 1922973	
Empirical formula	$C_{30} H_{32} Cl_2 N_2 O_3$	
Formula weight	539.47	
Temperature	173(2) K	
Wavelength	0.71073 Å	
Crystal system	Monoclinic	
Space group	*P* 2_1/*n*	
Unit cell dimensions	a = 11.7004(10) Å	α= 90°.
	b = 10.1618(9) Å	β= 101.425(7)°.
	c = 24.408(2) Å	γ = 90°.
Volume	2844.5(4) $Å^3$	
Z	4	
Density (calculated)	1.260 Mg/m^3	
Absorption coefficient	0.261 mm^{-1}	
F(000)	1136	
Crystal size	0.260 x 0.240 x 0.120 mm^3	
Theta range for data collection	3.358 to 25.670°.	
Index ranges	-14<=h<=13, -12<=k<=12, -29<=l<=29	
Reflections collected	15328	
Independent reflections	5333 [R(int) = 0.0428]	
Completeness to theta = 25.000°	99.7 %	
Absorption correction	Semi-empirical from equivalents	
Max. and min. transmission	1.000 and 0.378	
Refinement method	Full-matrix least-squares on F^2	
Data / restraints / parameters	5333 / 0 / 342	
Goodness-of-fit on F^2	0.958	
Final R indices [I>2sigma(I)]	R1 = 0.0490, wR2 = 0.1057	
R indices (all data)	R1 = 0.0862, wR2 = 0.1182	
Largest diff. peak and hole	0.765 and -0.577 e.$Å^{-3}$	

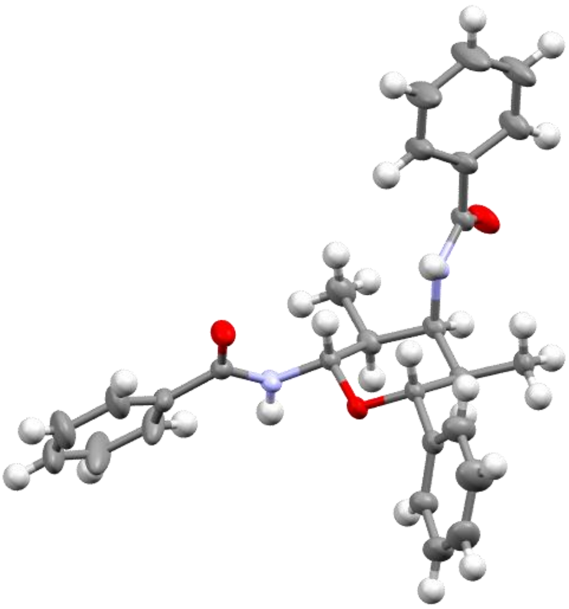

Table 5. Crystal data and structure refinement for pyrane **6a**. Displacement ellipsoids are drawn at the 50% probability level.

Identification code	CCDC 1922974	
Empirical formula	$C_{27.75} H_{28.75} Cl_{2.25} N_2 O_3$	
Formula weight	518.04	
Temperature	173(2) K	
Wavelength	0.71073 Å	
Crystal system	Triclinic	
Space group	*P* -1	
Unit cell dimensions	a = 13.4267(6) Å	α= 70.881(3)°.
	b = 15.3648(6) Å	β= 68.240(3)°.
	c = 15.8197(6) Å	γ = 64.721(3)°.
Volume	2684.6(2) $Å^3$	
Z	4	
Density (calculated)	1.282 Mg/m^3	
Absorption coefficient	0.298 mm^{-1}	
F(000)	1086	
Crystal size	0.190 x 0.160 x 0.120 mm^3	
Theta range for data collection	3.280 to 26.372°.	
Index ranges	-16<=h<=16, -19<=k<=19, -19<=l<=19	
Reflections collected	101995	
Independent reflections	10970 [R(int) = 0.0629]	
Completeness to theta = 25.000°	99.8 %	
Absorption correction	Semi-empirical from equivalents	
Max. and min. transmission	1.000 and 0.566	
Refinement method	Full-matrix least-squares on F^2	
Data / restraints / parameters	10970 / 24 / 665	
Goodness-of-fit on F^2	1.240	
Final R indices [I>2sigma(I)]	R1 = 0.0789, wR2 = 0.1735	
R indices (all data)	R1 = 0.0981, wR2 = 0.1822	
Largest diff. peak and hole	1.017 and -0.630 e.$Å^{-3}$	

5 References

[1] J. Halli, P. Kramer, M. Bechthold, G. Manolikakes, *Adv. Synth. Catal.* **2015**, *357*, 3321.

[2] Stoe & Cie, *X-AREA*. Diffractometer control program system. Stoe & Cie, Darmstadt, Germany, 2002.

[3] G. M. Sheldrick, *Acta Crystallogr. Sect. A*, 2008, **64**, 112−122.
